# Microbiota Succession and Chemical Composition Involved in the Radish Fermentation Process in Different Containers

**DOI:** 10.3389/fmicb.2020.00445

**Published:** 2020-04-03

**Authors:** Lei Liu, Xiao She, Xing Chen, Yang Qian, Yufei Tao, Yalin Li, Shuyu Guo, Wenliang Xiang, Guorong Liu, Yu Rao

**Affiliations:** ^1^School of Food Science and Bioengineering, Xihua University, Chengdu, China; ^2^Department of Wine and Food Engineering, Sichuan Technology and Business College, Dujiangyan, China; ^3^Beijing Engineering and Technology Research Center of Food Additives, Beijing Technology and Business University, Beijing, China

**Keywords:** fermented pickle, chemical composition, bacterial diversity, correlation analysis, different containers

## Abstract

Traditional Chinese fermented vegetables are a type of brine-salted fermented vegetable product. During the spontaneous fermentation, various compounds are produced, degraded, and converted, influencing the quality of the fermented pickle. To ascertain the effect of different containers on the fermentation process of the pickles, this study investigated the bacterial diversity and the chemical composition characteristics of the pickle (radish) fermented in commonly used containers including glass jars (GL), porcelain jars (PO), and plastic jars (PL). The correlation between chemical compounds and microbial community was further analyzed. The changes in pH values suggested that PL may facilitate the quickest fermentation of the pickles, while the process in PO progressed at the lowest rate. The PL brine samples contained higher levels of lactic acid and threonine, while more abundant volatile chemical compounds were evident in PO. The container materials had no significant influence on the microbial structure, wherein *Lactobacillus* was the absolute dominant genus in all containers. But container material did have an effect on the abundance of specific genus, such as *Lactococcus* and *Pediococcus*. The correlation between these major genera was also analyzed and gene function prediction indicated that the top three pathways were: carbohydrate metabolism, amino acid metabolism, and energy metabolism. *Lactobacillus* negatively correlated with methionine, tyrosine, lysine, and arginine, but positively correlated with ammonia, and lactic acid and acetic acid both just correlated with *Pediococcus*. This study provides new insights into the microbiota succession and chemical compounds involved in the vegetable fermentation.

## Introduction

Fermented pickle is a typical food type representative of traditional Chinese fermented food, which dates back to 3,000 years ago (Xiong et al., 2012). It is always served as a side dish accompanying an entree or alone as an appetizer (Rao et al., 2018). The fermented vegetable has attracted increasing attention in recent years due to the pleasant taste, nutritional value, and health benefits (Zhang et al., 2016). The manufacturing of pickle is a major industry in China, and in 2018 annual production reached 5 million tons in Sichuan province, with an annual value of over 42 billion RMB (6.2 billion dollars). Unlike sauerkraut and kimchi, this particular pickle is a type of brine-salted fermented vegetable product. Vegetables are typically immersed in 6–8% salt solution, and allowed to undergo spontaneous lactic acid fermentation for several days (Yan et al., 2008). The fermentation techniques may vary from place to place, but an essential element influencing the quality of the fermented product is the fermentation vessel. However, to date, whether the fermentation vessels contribute to the characteristics of the pickle has little been assessed.

Plastic is a polymer organic material that has been widely used in the food industry. The advantages of plastic containers are that they are light, cheap, and display excellent sealability (Westerhoff et al., 2008; Welle and Franz, 2011). Porcelain is a non-metallic inorganic material which exhibits properties such as porosity and good stability. Porcelain containers have a long history of being used as traditional fermentation containers, and are popular vessels utilized in both the homemade and industrial production of pickles. The industry has recently welcomed the addition of glass jars as fermentation containers due to their stability, as well as their excellent ability to facilitate the transmission of light (Schaut and Weeks, 2017). However, it remains unclear whether the types of containers can influence the fermentation process of pickles.

During spontaneous fermentation, various compounds are produced, degraded, and converted, influencing the quality of the fermented pickle. When the raw materials are exposed to lactic acid bacteria (LAB), the fermentation process is initialized, and lactic acid and acetic acid are produced, lowing the pH value (Xiong et al., 2012). The organic acid and free amino acid (FAA), as well as volatile chemical substances, influence the pickle quality (Zhang et al., 2016). The fermented pickle product relies on a delicate microbial balance. The interaction between the microbial community and chemical compounds promotes the fermentation. However, the correlation between them during the fermentation process is yet unclear.

Radish (*Raphanus sativus* L.), used in this study, is one of the major crops cultivated in Sichuan Basin in China, and has been fermented for over 1,500 years. Tons of this kind of pickle are annually produced for domestic consumption and for export. Different containers are used including glass jars (GL), porcelain jars (PO), and plastic jars (PL), which are most commonly applicable in the pickle fermentation. The physicochemical and microbiological characteristics are investigated during the fermentation. Furthermore, the correlation between the microbial community and chemical compounds is analyzed in an attempt to clarify the fermentation mechanism. The study could provide further insight into the fermentation mechanism of vegetables.

## Materials and Methods

### The Sampling of Fermented Radishes

Fresh radishes (*Raphanus sativus* L.) were purchased from local supermarkets in Chengdu, Sichuan Province, China. The radishes were washed using boiled water, drained, and then placed into 10 L GL, PO, and PL. Every jar contained about 3 kg radishes, of which the length and the maximum diameter were about 8 cm and 4 cm. Each treatment was repeated three times and 30 mL of old brine (containing 6% salt) was added to the respective jars together with cold boiled water containing 6% salt NaCl (w/v). The jars were covered, sealed with water, and left to ferment at room temperature (about 22 ~ 25°C). The brine samples were collected by sterile operation and stored in sterile tubes at −20°C.

### Determining the PH Value and Nitrite Content

The pH values of the brine samples were determined daily for the first 12 days using a pH meter (PHS-3C, Fangzhou Technology, China), calibrated using standard buffer solutions at pH 4.00, pH 6.86, and pH 9.18. Hydrochloride naphthodiamide was used to determine the concentration of nitrite per kg, according to GB 5009.33-2016 (Mao et al., 2018).

### Determining the Texture of the Radishes

The fermented radishes were divided into four parts and were cut into cuboids measuring 3 cm wide, 3 cm long, and 2 cm thick. The texture was examined using a TA.XT Express Texture Analyzer (Stable Micro Systems, Surrey, UK) equipped with a P/5 probe, via a compression test that simulates the test done by the consumer when evaluating the product. The rate before testing was 30 mm/min, the test rate was 20 mm/min, and the rate after testing was 30 mm/min, while the compression strength was 5 g, and the compression amount was 50%.

### Determining the Concentrations of Organic Acids and Free Amino Acids

The organic acid concentrations (oxalic acid, lactic acid, acetic acid, butyric acid, and ketoglutaric acid) of brine samples were determined using high-performance liquid chromatography (HPLC, Waters 2695, USA). The organic acid samples were detected with a UV detector at 210 nm, and were separated with an Aminex HPX-87H Column (300^*^7.8 mm, Bio-Rad) which was maintained at 60°C, using 0.005 M sulfuric acid as a mobile phase at a flow rate of 0.6 mL/min. The brine samples (2 mL) were defrosted and centrifuged (8,000 rpm, 10 min), after which the supernatant was filtered through a 0.22 μm filter, and 10 μL was injected into the autosampler vials. The organic acids were identified and quantified by comparing their retention times and peak areas with standards.

To quantify the content of free amino acids (FAA), the brine samples during the fermentation (0, 6, and 12 days) were collected. Aspartic acid, threonine, serine, glutamic acid, glycine, alanine, cystine, proline, methionine, isoleucine, leucine, tyrosine, phenylalanine, lysine, histine, argine, and ammonia were included. 0.22 μm membrane filters were used to filter 1 mL of the sample (Toyo Roshi Kaisha Ltd, Tokyo, Japan). The FAA content was analyzed using Hitachi L-8900 Amino Acid Analyzer (Hitachi High-Technologies Co. Ltd., Tokyo, Japan) equipped with a column (Hitachi, 4.6 × 60 mm) packed with custom ion exchange resin (#2630, anionic) and a UV detector (570 and 440 nm). The column was operated at 37°C with a flow rate of 0.3 mL/min. The composition of each peak was determined based on a standard curve prepared from external standards.

### Determining the Concentrations of Volatile Compounds

The volatile compounds of the brine samples during different fermented stages (0 d, 6 d, and 12 d) were determined using gas chromatography-mass spectrometry (GCMS-QP2010 Plus, Shimadzu, Japan) according to previous research, with modifications (Xiao et al., 2018). In brief, 5.0 g samples were placed in 25 mL headspace vials (CNW, Germany) with 10 μL 2-methyl-3-heptanone (170 μg/L) as the internal standard. Following an equilibration time of 15 min at 40°C using an MP-501A super constant temperature circulation tank (Shanghai, China), the fiber (A SUPELCO 75 μm Carboxen, Sigma-Aldrich, USA) was exposed to the headspace for 30 min at 40°C. Desorption was performed within 5 min in splitless mode at 250°C. A GC (gas chromatography) (GC-2010 plus, Shimadzu, Japan) fitted with a quadrupole MS (mass spectrometry) (GCMS-QP2010 Plus, Shimadzu, Japan) utilizing a RET-5 capillary column (30 m, 0.32 mm, 0.25 μm thickness; Restek, Bellefonte, PA, USA) was used. Helium was employed as the carrier gas at a constant linear velocity of 1.20 mL/min. The temperature program included the following: 36°C (3 min), from 36°C to 160°C at 8°C/min, maintained for 2 min at 160°C, from 160 to 250°C at 13°C/min, and maintained for 7 min at 250°C. The MS analysis was conducted in the electron ionization mode: ionization energy of 70 eV, ion source temperature of 230°C, interface temperature of 280°C, and a scan range of 30–450 a/m. All samples were run in triplicate. The NIST 2011 standard mass spectral database combined with Chemical Book website (https://mip.chemicalbook.com/) was used to identify the volatile organic compounds based on the retention times and mass-spectral similarity matches (more than 85%).

### Determining the Microbial Diversity

#### Target DNA Fragment Amplification and Illumina MiSeq Sequencing

Total genomic DNA was extracted from the samples using a DNeasy PowerSoil Kit (QIAGEN, DE), according to the instructions. The DNA concentration and quality were reviewed using a NanoDrop Spectrophotometer and 16S rRNA genes were amplified using a specific primer with a 12 nt unique barcode. Three PCR reaction replicates for each sample were combined, and samples displaying a bright main strip between 200 and 400 bp were selected for further experiments.

The electrophoresis band was purified using an OMEGA Gel Extraction Kit (Omega Bio-Tek, USA), while DNA was quantified with a Qubit@ 2.0 Fluorometer (Thermo Scientific, USA). PCR products from different samples were pooled with an equal molar amount. Sequencing libraries were generated using a TruSeq DNA PCR-Free Sample Prep Kit following the manufacturer's recommendations, and index codes were added. The library quality was assessed on the Qubit@ 2.0 Fluorometer (Thermo Scientific, USA) and the Agilent Bioanalyzer 2100 system. Finally, the library was applied to paired-end sequencing (2 × 250 bp) with the Illumina HiSeq apparatus at Rhonin Biosciences Co., Ltd.

#### Sequencing Data Analysis

Sequences were assigned to each sample according to the unique barcode. Low-quality reads (length < 200 bp, more than two ambiguous bases “N,” or average base quality score < 30) and truncated sequences where the quality scores decayed (score < 11), were filtered. Sequences were clustered into operational taxonomic units (OTUs) with a 97% identity threshold using UPARSE algorithms (Edgar, 2013). The Uchime algorithm was used to select representative sequences, as well as potential chimeras that were removed (Edgar et al., 2011).

Taxonomy was assigned using the Silva database (Quast et al., 2013) and Uclust classifier in QIIME software (Version 2-2017.8). Regarding the influence of sequencing depth on community diversity, the OTU table was rarified to ensure that all samples were holding the same sequence number. Beta-diversity metric including Principle Component Analysis (PCA) was calculated in Vegan packages in R software (V2.15.3) (Kembel et al., 2010). Partial Least Square Discriminant Analysis (PLS-DA) was performed using mixOmics package in R software (http://fiehnlab.ucdavis.edu/staff/kind/Statistics/Concepts/OPLS-PLSDA). Heatmap showed the microbial distribution at genus level and was constructed using the Vegan in R software. Circos was presented using Circos software (Version 0.67-7, http://circos.ca/), which is used to visualize the correspondence between samples and species. The rarefaction analysis and Beta diversity were analyzed through platform of Majorbio Cloud Platform (www.majorbio.com).

Phylogenetic Investigation of Communities by Reconstruction of Unobserved States (PICRUSt) is used to predict the functional content of microbial communities based on the sequencing data (Langille et al., 2013). The COG (Clusters of Orthologous Groups) and KEGG pathways (Kyoto Encyclopedia of Genes and Genomes) were predicted on the free online platform of Majorbio Cloud Platform (www.majorbio.com). The correlation intra genus was conducted using Spearman Rank Correlation Coefficient and presented by heatmap on Majorbio Cloud Platform as well.

#### Nucleotide Sequence Accession Numbers

The sequence of 16S rRNA genes hypervariable region were deposited in the Sequence Read Archive (SRA) of the NCBI database, and the accession number is SRP225177.

#### Statistical Analysis

Three repetitions were performed for the pickle fermentation process in each container. All data were shown as means for at least three independent experiments, and P-values less than 0.05 were considered statistically significant. The graph presentations were generated using the SPSS 13.0, Origin Software 8.0, and the GraphPad Prism 7. Relationships between the different genera and chemical compositions were calculated using the Pearson Rank Correlation Coefficient using the OmicShare tools, a free online platform for data analysis (http://www.omicshare.com/tools). Cytoscape (Version 3.7.1) was employed to visualize the interaction networks between bacterial communities and metabolites during the fermentation process in different containers.

## Results

### Changes in PH Value and Nitrite Content

**Figure 1:**
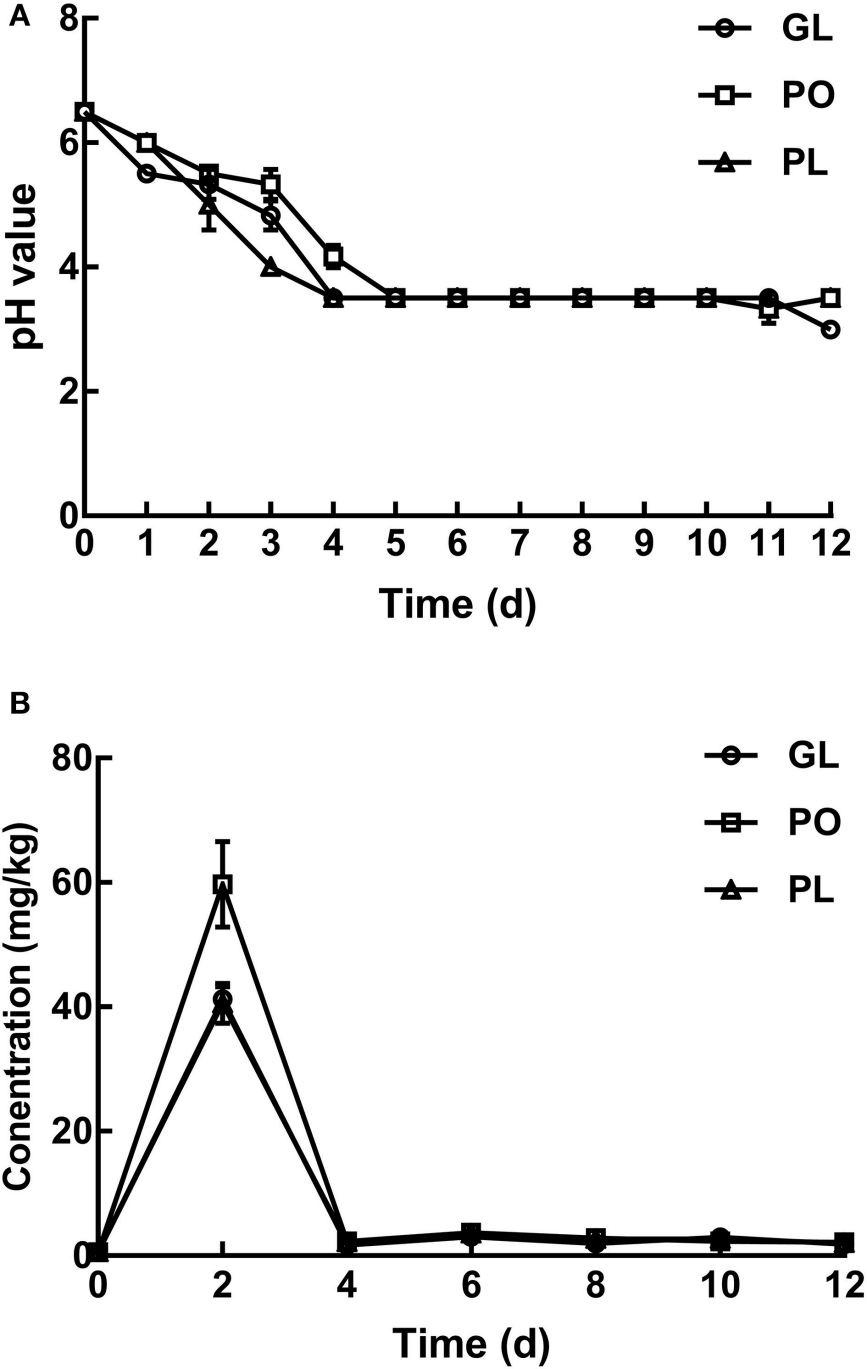
Changes in the pH values **(A)** and nitrite content **(B)** in different containers during the fermentation process.

As shown in Figure 1A, the mean pH value of the brine samples in GL, PO, and PL initially declined rapidly, and then stabilized during the subsequent days. The pH value of the brine displayed the quickest decline in PL while the slowest decrease was evident in PO. Moreover, the pH levels reached a minimum value of 3.5 on the 4th day in GL and PL, while the pH in PO only reached the same value on the 5th day.

According to Figure 1B, the nitrite concentrations in the containers increased to the maximum levels on the 2nd day, and reached 59.73 mg/kg in PO, which was significantly higher than the values in both the other containers. Furthermore, the nitrite content in the containers declined significantly to an extremely low level on the 4th day, while it stabilized during the subsequent days.

### Changes in Texture

**Figure 2:**
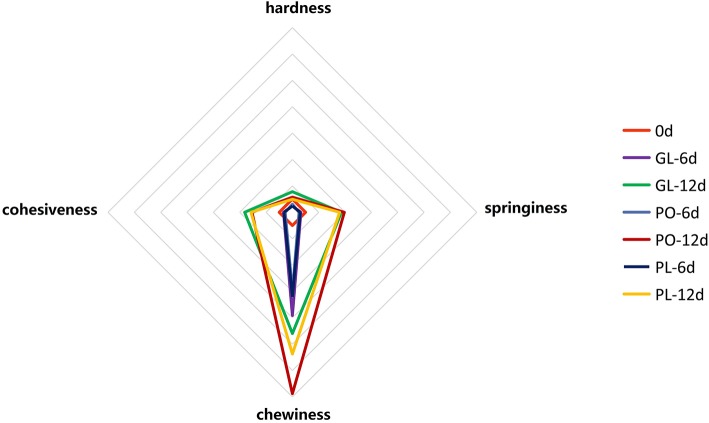
The hardness, cohesiveness, chewiness, and springiness of pickles in different containers on days 0, 6, and 12.

Figure 2 illustrates the texture changes during the fermentation process of radishes in different containers, including the firmness, springiness, chewiness, and cohesiveness. The chewiness of the radish samples displayed significant changes in all the containers. Compared to its value on day 0, it increased 7.83-fold, 6.00-fold, and 6.42-fold in GL, PO, and PL, respectively, on the 6th day. This increasing trend continued, reaching 9.20-fold, 13.76-fold, and 10.74-fold in GL, PO, and PL, respectively, on the 12th day. The springiness and cohesiveness of samples in these containers reduced by half on the 6th day, and then increased- three- to fourfold on the 12th day. No significant differences were observed in the values denoting the springiness and cohesiveness of the radish samples in the containers on either the 6th day or the 12th day. The value signifying firmness in PO and PL declined on the 6th day and increased to the original level on the 12th day, while it was 0.74-fold in GL compared to the original value. Detailed data was listed in Table S1.

### Changes in the Concentrations of Organic Acids and FAA

**Figure 3:**
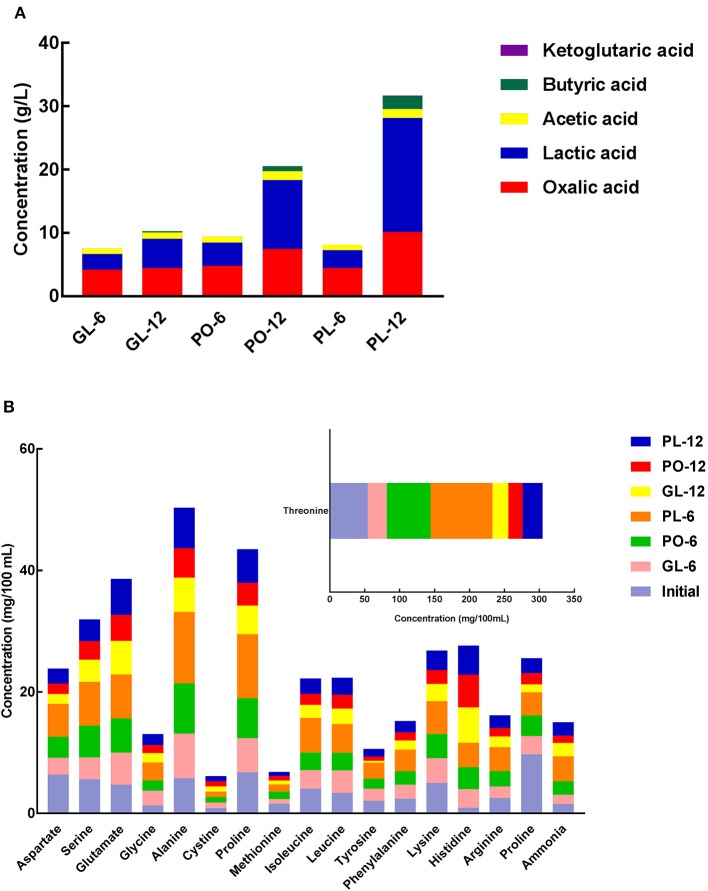
The concentration of organic acids **(A)** and FAA **(B)** in different containers on days 0, 6, and 12.

The organic acid concentrations in the brine samples from the different containers are shown in Figure 3A, and include oxalic acid, lactic acid, acetic acid, butyric acid, and ketoglutaric acid. No significant difference was evident in the oxalic acid concentrations of GL, PO, and PL on the 6th day. However, a significant increase to about 10.13 g/L and 7.50 g/L was apparent on the 12th day in PL and PO, respectively. Moreover, the lactic acid content in the containers significantly increased on the 12th day. It increased mostly to about 18.04 g/L in PL and 10.89 g/L in PO. The acetic acid content of GL, PO, and PL exhibited no significant difference on the 6^th^ day but increased slightly on the 12th day. The presence of butyric acid was only detectable on the 12th day and was primarily identified in PL (2.074 g/L). Furthermore, all containers displayed extremely low concentrations of ketoglutaric acid during the fermentation process. Detailed data was listed in Table S2.

The changes in FAA levels are shown in Figure 3B. The results indicate that 16 free amino acids were detected, including methionine, isoleucine, leucine, tyrosine, phenylalanine, histidine, arginine, lysine, valine, threonine, serine, glycine, alanine, proline, aspartate, and glutamate. In addition, cystine and ammonia were also identified in the samples while tryptophan was not detected in any of the samples during the fermentation process. Threonine was the most abundant amino acid in all samples and increased to 88.88 mg/100 mL and 62.48 mg/100 mL on the 6th day but declined to 28.78 mg100 m/L and 20.53 mg/100 mL on the 12th day in PL and PO, respectively. Furthermore, the threonine content in GL decreased to 27.04 mg/100mL on the 6^th^ day and continued to decline until it reached 22.54 mg/100 mL on the 12th day. The glutamate concentrations displayed no significant change in any of the containers during the fermentation process. Detailed data was listed in Table S3.

### Changes in Volatile Chemical Compounds

**Figure 4:**
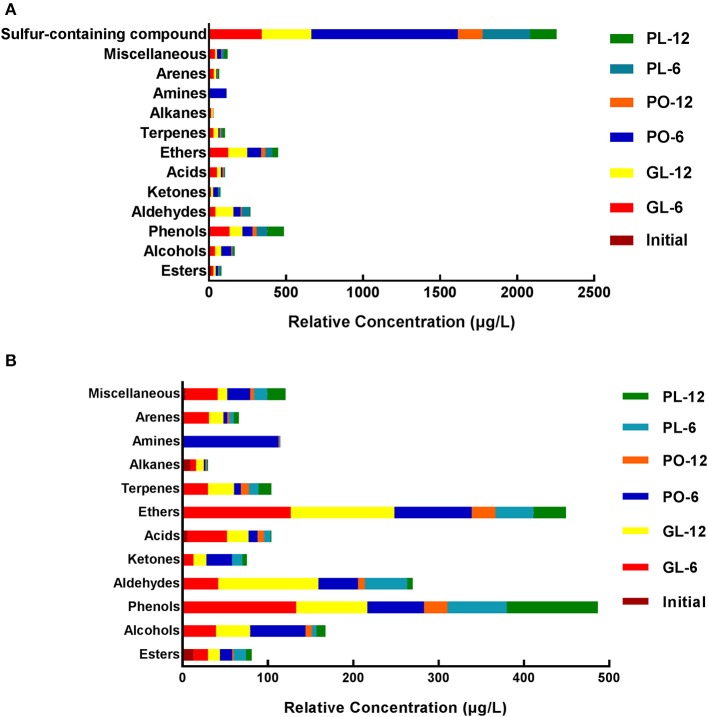
The relative concentration of volatile flavor compounds, including sulfur-containing compounds **(A)** and excluding sulfur-containing compounds **(B)** in different containers on days 0, 6, and 12.

The data provided by SPME-GC/MS (solid-phase microextraction gas chromatography-mass spectrometry) analysis of the brine in GL, PO, and PL after 0 days, 6 days, and 12 days are shown in Figure 4, and detailed information is listed in Table S4. The volatile compounds were divided into 13 groups: sulfur-containing compounds, miscellaneous, arenes, amines, alkanes, terpenes, ethers, acids, ketones, aldehydes, phenols, alcohols, and esters.

Sulfur-containing compounds were the most abundant in the samples of this study. On the 6^th^ day of fermentation, the brine in PO exhibited more sulfur-containing compounds than GL and PL. However, on the 12th day, the brine in GL contained the highest levels of this component, whilst its concentrations were at similar levels in the brine of PO and PL. Besides sulfur-containing compound classes, the top four compounds were phenols, ethers, aldehydes, and alcohols. Phenols increased significantly on the 6th day, and then decreased on the 12th day in GL and PO, but increased during the fermentation process in PL. The levels were higher in GL than in PO and PL on the 6th day, while they were most prominent in PL on the 12th day. Ethers increased significantly in GL and PL on the 6th day, but remained unchanged on the 12th day, while elevated levels were evident in PO on the 6th day that declined on the 12th day in PO. Moreover, these levels were higher in GL than in the other containers. During the fermentation process, aldehydes increased in GL, while it exhibited an initial increase followed by a significant decline in PO and PL. The aldehyde content was the most abundant in GL on the 12th day. On the 6th day the brine samples in PO contained more alcohols than the samples from the other containers. The initial brine contained several alkanes, which remained at a constant level in GL during the fermentation process, but decreased significantly in PO and PL. On both the 6th and 12th day, the GL brine samples contained more terpenes than those from the other containers. The acid content was higher in GL than in the other jars during the fermentation process. The PO brine samples contained more ketones on the 6th day than those from the other containers. Both the content levels and types of esters were the highest in PO, followed by GL.

### Microbiological Diversity Analysis

**Figure 5:**
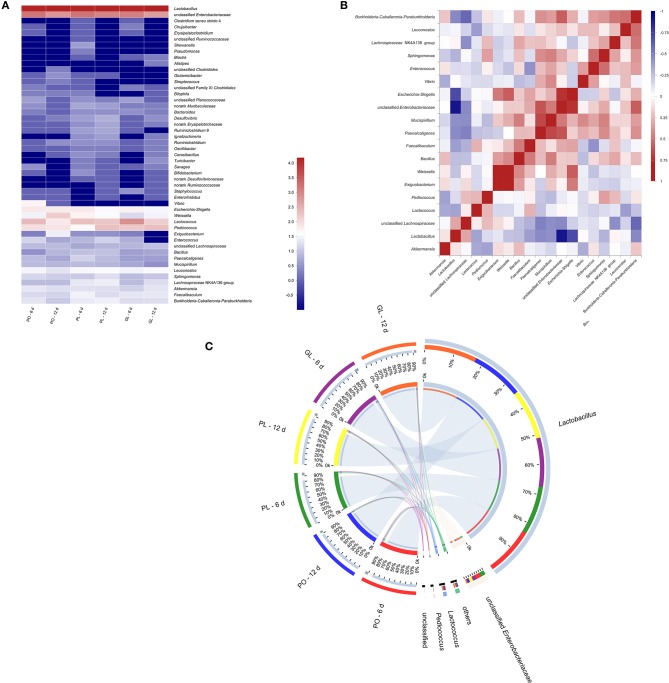
Heatmap of bacterial diversity at the genus level in different containers **(A)**. Correlation matrix of the Spearman's rank correlation among the major genera **(B)**, The Spearman's rank correlation coefficient ranges from 1.0 to −1.0, corresponding to strongly positive correlation to strongly negative correlation. Circular representation of bacterial community in different containers at genus level **(C)**. The length of the bars of each sample on the outer-ring represented the percentage of genus in each sample. Only the genus with the abundance >1% in at least one sample were shown here.

The high throughput sequencing revealed the bacterial communities in all brine samples in GL, PO, and PL after fermentation of 6 and 12 days. The Principal Component Analysis (PCA) and Partial Least Squares Discrimination Analysis (PLS-DA) analysis were shown in Figures S1, S2. The rarefaction analysis was showed in Figure S3. Bacterial communities at genus level are shown in Figure 5A, suggesting that the dominant genera were similar in these different containers. The CLR transformed abundance of OTU was listed in Table S5. Among the identified genera, *Lactobacillus* absolutely dominated in all samples. The genera with a relatively high abundance were unclassified *Enterobacteriaceae, Lactococcus, Pediococcu*s, and *Weissella*. In addition, the abundance of *Vibrio* was higher in PO than in others. In order to determine the correlation between the genera, the data of top 20 genera were analyzed through the Spearman's rank method and shown in correlation matrix in Figure 5B. The result indicated that *Lactobacillus* correlated strongly negatively with unclassified *Enterobacteriaceae, Escherichia-Shigella*, and *Burkholderia-Caballeronia-Paraburkholdera*. Unclassified *Enterobacteriaceae* correlated strongly positively with *Escherichia-Shigella, Burkholderia-Caballeronia-Paraburkholdera*, and *Paenalcaligenes*. *Escherichia-Shigella* positively correlated with *Exiguobacterium, Weissella*, and unclassified *Enterobacteriaceae*. Figure 5C illustrated the abundance of the major genera (the average abundance >1% at least one type of containers) observed in different containers in a circular viewer. The results showed that *Lactobacillus* dominated 85% in all containers on the 6th day and dominated 92% in GL and PO on the 12th day, while it was 89% in PL on the 12th day. Unclassified *Enterobacteriaceae*, the second dominant genus, slightly declined on the 12th day compared with the 6th day, from 7.2% to 3.2 in GL, 9.9% to 6.5% in PL, and 9.5% to 4.1% in PO, respectively.

### Gene Function Characterization

**Figure 6:**
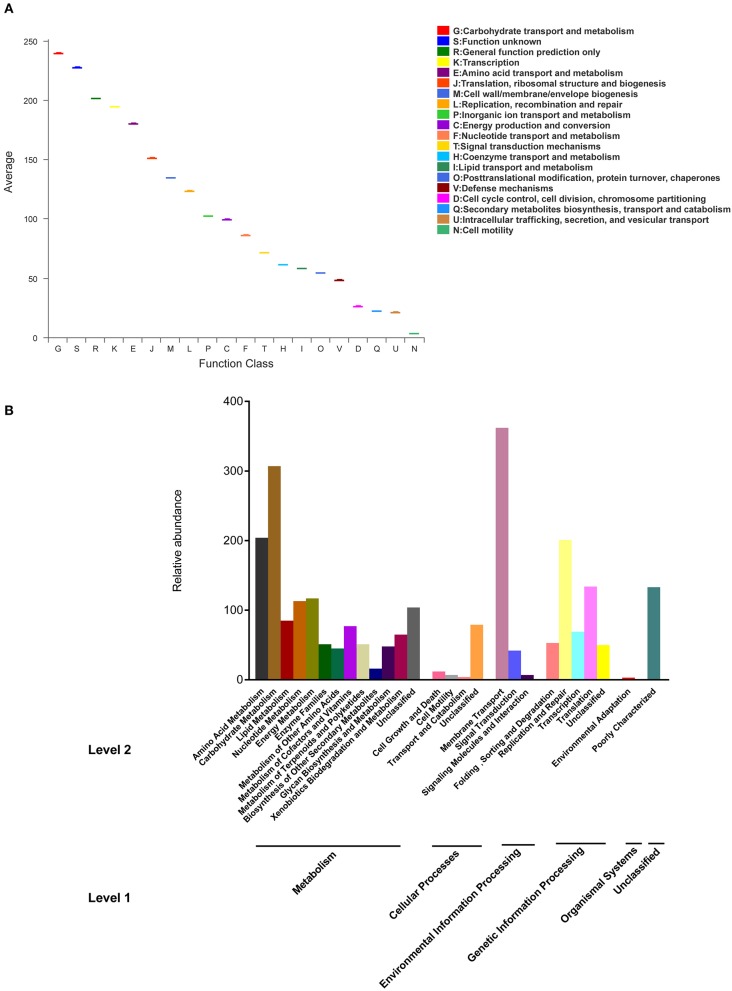
Gene function annotation **(A)** of microbial community, KEGG pathways of metabolism **(B)** at level 1 and 2.

The functions of bacterial genes and KEGG pathways of metabolism were predicted by PICRUSt. The gene function annotation of microbial community and KEGG pathways were showed in Figures 6A,B. 25 types of gene functions were evaluated and the major functional profiles of microbial genes were classified into carbohydrate transport and metabolism, transportation, amino acid transport and metabolism, translation, ribosomal structure and biogenesis, replication, recombination and repair, cell wall/membrane/envelop biogenesis, and other unclear functions (Figure 6A). Carbohydrate transport and metabolism was noted to have the highest abundance. KEGG pathway was analyzed at level 1, including metabolism, cellular processes, genetic information processing, environmental information processes, organism systems, and other unclassified pathways (Figure 6B). Among metabolism, the top 3 pathways were carbohydrate metabolism, amino acid metabolism, and energy metabolism. In addition, the abundance of genes related to membrane transport was high in the samples.

### Association Between Bacterial Communities and Chemical Compounds

**Figure 7:**
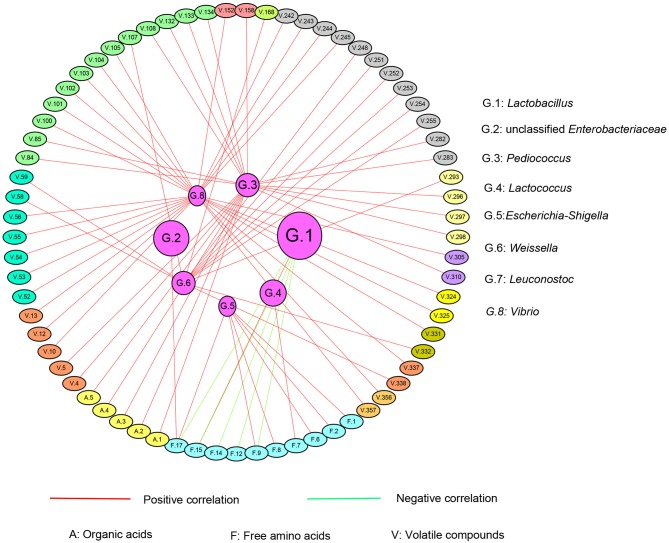
The correlated network between bacteria genera and chemical compounds of the fermented pickles. The inner-side circle represents the bacteria genera, and the out-side circles represent different chemical compounds. The red lines linking the circles represent the positive correlation while those in green represent the negative correlation between the bacterial community and chemical compounds.

The correlation between chemical characteristics and the major genera including *Lactobacillus* (G.1), unclassified *Enterobacteriaceae* (G.2), *Pediococcus* (G.3), *Lactococcus* (G.4), *Escherichia-Shigella* (G.5), *Weissella* (G.6), *Leuconostoc* (G.7), and *Vibrio* (G.8) were observed. An index over 0.6 was considered to be correlated (Figure 7). The details of volatile chemical compounds, amino acids, and organic acids are shown in Table S4. The correlation coefficient and *P*-value were listed in Table 1. Except *Leuconostoc* (G.7), other genera correlated with specific chemical substances.

**Table 1:** The correlation between genera and chemical compounds.

**Genus**	**Interaction**	**Compounds**	**Coefficient**	***P-*value**
G.1	Negative	F.9	−0.601491209	0.008275435
G.1	Negative	F.12	−0.675066821	0.002113123
G.1	Negative	F.14	−0.623146906	0.005731856
G.1	Positive	F.15	0.656812132	0.003064342
G.1	Negative	F.17	−0.693586968	0.001411551
G.2	Positive	F.17	0.610953703	0.007070858
G.3	Positive	V.4	0.624536657	0.005593321
G.3	Positive	V.5	0.741059848	0.000433751
G.3	Positive	V.84	0.618261921	0.00624109
G.3	Positive	V.85	0.701738676	0.001170999
G.3	Positive	V.108	0.708340499	0.001002108
G.3	Positive	V.132	0.810281802	4.56E-05
G.3	Positive	V.133	0.741059848	0.000433751
G.3	Positive	V.134	0.741059848	0.000433751
G.3	Positive	V.158	0.741059848	0.000433751
G.3	Positive	V.282	0.741059848	0.000433751
G.3	Positive	V.283	0.741059848	0.000433751
G.3	Positive	V.296	0.741059848	0.000433751
G.3	Positive	V.297	0.741059848	0.000433751
G.3	Positive	V.298	0.741059848	0.000433751
G.3	Positive	V.310	0.741059848	0.000433751
G.3	Positive	A.1	0.742769851	0.000413809
G.3	Positive	A.2	0.630011362	0.005073784
G.3	Positive	A.3	0.785242087	0.00011305
G.3	Positive	A.4	0.812108328	4.25E-05
G.3	Positive	A.5	0.747644926	0.000361131
G.4	Positive	F.7	0.625898218	0.005460246
G.4	Negative	F.15	−0.623983893	0.005648093
G.5	Positive	F.1	0.645465923	0.003815212
G.5	Positive	F.2	0.638097091	0.004378894
G.5	Positive	F.6	0.621809816	0.00586776
G.5	Positive	F.8	0.601008025	0.008341118
G.5	Positive	F.9	0.662647705	0.002728251
G.5	Positive	F.17	0.650398203	0.003472201
G.6	Positive	V.58	0.69880271	0.001253365
G.6	Positive	V.59	0.956850582	5.28E-10
G.6	Positive	V.107	0.956850582	5.28E-10
G.6	Positive	V.168	0.956850582	5.28E-10
G.6	Positive	V.248	0.623377308	0.005708698
G.6	Positive	V.251	0.956850582	5.28E-10
G.6	Positive	V.252	0.956850582	5.28E-10
G.6	Positive	V.253	0.956850582	5.28E-10
G.6	Positive	V.254	0.956850582	5.28E-10
G.6	Positive	V.255	0.956850582	5.28E-10
G.6	Positive	V.293	0.956850582	5.28E-10
G.6	Positive	V.332	0.956850582	5.28E-10
G.6	Positive	V.338	0.956850582	5.28E-10
G.8	Positive	V.10	0.621875958	0.005860976
G.8	Positive	V.12	0.955242523	7.03E-10
G.8	Positive	V.13	0.938066391	8.95E-09
G.8	Positive	V.52	0.955242523	7.03E-10
G.8	Positive	V.53	0.955242523	7.03E-10
G.8	Positive	V.54	0.955242523	7.03E-10
G.8	Positive	V.55	0.955242523	7.03E-10
G.8	Positive	V.56	0.955242523	7.03E-10
G.8	Positive	V.100	0.955242523	7.03E-10
G.8	Positive	V.101	0.955242523	7.03E-10
G.8	Positive	V.102	0.955242523	7.03E-10
G.8	Positive	V.103	0.955242523	7.03E-10
G.8	Positive	V.104	0.955242523	7.03E-10
G.8	Positive	V.105	0.955242523	7.03E-10
G.8	Positive	V.152	0.955242523	7.03E-10
G.8	Positive	V.242	0.955242523	7.03E-10
G.8	Positive	V.243	0.955242523	7.03E-10
G.8	Positive	V.244	0.955242523	7.03E-10
G.8	Positive	V.245	0.955242523	7.03E-10
G.8	Positive	V.305	0.955242523	7.03E-10
G.8	Positive	V.324	0.955242523	7.03E-10
G.8	Positive	V.325	0.955242523	7.03E-10
G.8	Positive	V.331	0.955242523	7.03E-10
G.8	Positive	V.337	0.955242523	7.03E-10
G.8	Positive	V.356	0.955242523	7.03E-10
G.8	Positive	V.357	0.955242523	7.03E-10

*G, Genus; A, Organic acids; F, Free amino acids; V, Volatile compounds*.

*G.1, Lactobacillus; G.2, unclassified Enterobacteriaceae; G.3, Pediococcus; G.4, Lactococcus; G.5, Escherichia-Shigella; G.6, Weissella; G.7, Leuconostoc; G.8, Vibrio*.

*Detailed information of chemical compounds was shown in Table S4*.

The absolute predominant genus *Lactobacillus* negatively correlated with methionine (F. 9), tyrosine (F. 12), lysine (F. 14), and arginine (F. 17), while it positively correlated with ammonia (F. 15). The second highest abundant genus, unclassified *Enterobacteriaceae*, was only positively correlated with arginine (F. 17). It should be noted that some lesser dominant genera correlated with more compounds compared with *Lactobacillus* and unclassified *Enterobacteriaceae. Pediococcus* (G. 3) positively correlated with 15 kinds of volatile compounds and 5 kinds of organic acids. *Lactococcus* (G.4) positively and negatively correlated with cystine (F.7) and arginine (F.17), respectively. *Escherichia-Shigella* (G.5) positively associated with 6 kinds of FAA. *Weissella* (G. 6) showed a positive correlation with 13 kinds of volatile compounds. As for *Vibrio* (G.8), it positively correlated with 25 kinds of volatile compounds. From the aspect of the strength of correlation, the coefficient of the correlation between *Weissella* (G. 6) and 11 kinds of volatile compounds and *Vibrio* (G.8) and 25 kinds of volatile compounds were over 0.9 with the *P*-value less than 0.0001, suggesting an extreme correlation between them. As for lactic acid (A. 1) and acetic acid (A. 2), they both only correlated with *Pediococcus* (G. 3).

## Discussion

The present study investigates the physical and chemical changes, as well as the microbiological diversity, during the fermentation process in GL, PO, and PL, and the correlation between the microbial communities and chemical compounds in these containers are further analyzed using statistical methods and bioinformatic tools.

The pH value is vital since it affects the relative growth rates of the different microorganisms and the accumulation of metabolic products, which can influence the fermentation process of pickles (Ross et al., 2002; Kang et al., 2003; Wu et al., 2015). In this study, the quickest decrease in pH is evident in PL, indicating that microbial growth, especially LAB, is different in these containers. In addition, LAB can utilize soluble proteins by secreting peptide enzymes and breaking down the protein into a variety of FAA. Previous research indicates that alanine, aspartic acid, glutamic acid, glycine, proline, and serine are the predominant amino acids responsible for aroma and flavor production in kimchi (Kader, 2008). In this study, aspartic acid and glycine are not abundant. During the homolactic and heterolactic fermentation with LAB, different types of organic acids are produced. The predominant organic acid in the samples of this study is lactic acid, while acetic acid concentrations remain at low levels. Although ketoglutaric acid has previously been found in pickled wax gourd (Wu et al., 2016), its concentration was low in the samples of this study. Although butyric acid may be associated with the spoilage of fermented cucumbers (Medina et al., 2016), and was not found in the samples from any of the containers on the 6th day, it was evident in low concentrations in all containers on the 12th day.

In this study GC-MS is used to identify the volatile flavor components in the brine. Sulfides are considered important olfactory compounds in fermented foods, and the variations in these compounds can lead to either consumer acceptance or rejection (Zhao et al., 2016). Furthermore, dimethyl disulfide, dimethyl trisulfide, and dimethyl tetrasulfide are the primary sulfur-containing compounds detected during this study. Hawer et al. reported that dimethyl disulphellode, dimethyl trisulfide, and dipropyl disulfide are the main flavor compounds in Kimchi (Hawer et al., 1988), while isothiocyanate is considered to be the specific flavor compound in cruciferous vegetables and their processed products containing b-D-Glucopyranose (e.g., radishes) (Radomir et al., 1997). In addition, although no previous reports exist regarding the presence of piperidine-2-thione and tetrahydropyrrole-2-thione in the samples, many studies are available involving the esters found in some fruits, vegetables, and their processed products (Zhao et al., 2007). Furthermore, this compound is considered an important olfactory element in fermented foods with fruity odors, which are derived from the esterification of free fatty acids and alcohols (Xiao et al., 2010). Dibutyl phthalate and isobornyl acetate have been detected in Zhacai and another forgotten vegetable, *Smyrnium olusatrum* L. Apiaceae (Maggi et al., 2012). In addition, this study denotes the presence of D,L-isobornyl acetate, butyl valerate, diethyl 2,2-difluoromalonate, and N-octyl acrylate for the first time. Aldehydes produce desirable aromas which are mainly associated with sweet, fruity, nutty, and caramel-like odors (Waller and Feather, 1983). These compounds are considered to enhance flavor quality and can be produced by lipid oxidation and degradation during fermentation (Jennifer and Glesni, 1984). Marsili et al. reported that the brine of fermented cucumbers contains dodecyl aldehyde (Marsili and Miller, 2000). Although ketones are common in fermented foods, those identified in the current study have never been reported previously, and include D-camphor, 3-hexanone, and (-)-camphor. Of the acids detected here, 2-Methyl-Propanoic acid has been found in Chinese sauerkraut and fermented radishes (Wu et al., 2014), while nonanoic acid mainly contributes to the oily notes in fermented vegetables and has been identified in pickled chilis (Xiao et al., 2010). Furthermore, phenols are responsible for smokey and phenolic odors, but the presence of 3,5-Di-tert-Butylphenol and 2,6-Di-tert-butyl-4-methylphenol have never been reported previously.

The spontaneous fermentation of vegetables leads to the complex structure of microorganisms. *Firmicutes* and *Proteobacteria* are the primary phyla in the present study, which is consistent with previous findings (Cao et al., 2017; Liang et al., 2018a). At the genus level, in accordance with most available research, *Lactobacillus* dominates in all samples (Kim and Chun, 2005; Lee et al., 2018; Yang et al., 2018). Compared with other research, the abundance of *Lactobacillus* was much higher (at least > 85%), which may be due to our initial aged brine. In addition, unclassified *Enterobacteriaceae* is the second highest genus found in the samples of this study. *Leuconostoc* and *Weissella* were identified as the dominant genera in kimchi (Plengvidhya et al., 2007; Xiong et al., 2012), they were also detected in this study but with a relatively low abundance. *Pediococcus* commonly increased with the ongoing fermentation in pickles (Hong et al., 2016; Cao et al., 2017; Liang et al., 2018a), which agrees with the change trend in this study, especially in PL. In addition, and in line with other previous research (Liang et al., 2018a), *Vibrio* was also detected in our samples and its population declined along with the fermentation process. Furthermore, our results indicated that the container materials had no significant influence on the overall microbial structure of the fermented radish but had an effect on the abundance of specific genus, such as *Lactococcus* and *Pediococcus*.

There is some research considering the correlation between species (Peng et al., 2018; Yang et al., 2018); here, the correlation among the major genera were determined. According to previous studies, different species, although they belong to the same genus, still have opposite correlations with other species. For example, *Pediococcus pentosaceus* positively correlated with *Lactobacillus plantarum*, but negatively correlated with *Lactobacillus fermentum* (Peng et al., 2018). In the present study, at the genus level, *Lactobacillus* negatively correlated with *Escherichia-Shigella* and unclassified *Enterobacteriaceae*. PICRUSt was also used to predict the gene functions and KEGG pathways of microbiota. The microbial metabolism of carbohydrate, lipids, and proteins produce the complex compounds which form the sensory properties of fermented food (Liang et al., 2018b). Previous research had reported that carbohydrate metabolism, amino acid metabolism, nucleotide metabolism, and energy metabolism were the main pathways in metabolism (Liang et al., 2018a,b; Peng et al., 2018). Our results agreed with these findings, indicating that the top 3 pathways were carbohydrate metabolism, amino acid metabolism, and energy metabolism. In addition, we also found that the abundance of genes related to membrane transport was high as well. However, PICRUSt prediction just depends on the database and mathematics method, which could not reflect the whole array of functional genes involved in metabolism pathways completely and precisely. Therefore, further research needs to be conducted in order to obtain a much more thorough understanding.

Analysis regarding the correlation between microbial communities and metabolites remains limited. Xiao et al. suggested that bacteria, rather than fungi, may be the main producer of Sichuan pickle flavors (Xiao et al., 2018). Therefore, bacterial rather than fungal diversity, as well as its correlation with chemical compounds, are analyzed in the current study. Although *Lactobacillus* was commonly considered as the main producer of lactic acid, our correlation analysis suggested that no correlation existed between *Lactobacillus* and lactic acid. This is not the first report to show this. In one piece of similar research, all identified *Lactobacillus* species including *Lactobacillus buchneri, Lactobacillus versmoldensis, Lactobacillus brevis, Lactobacillus namurensis*, and *Lactobacillus acetotolerans* failed to correlate with lactic acid (Yang et al., 2018). This study also reported that *Pediococcus ethanolidurans* negatively correlated with lactic acid (Yang et al., 2018). However, our results indicated that *Pediococcus* genus positively correlated with lactic acid and acetic acid as well. Furthermore, this study suggested that *Weissella* may strongly correlate with 11 kinds of volatile compounds, although its abundance was not high. It should be noted that these correlations between microbiology and chemical compositions were calculated based on bioinformatic analysis, which may not prove the direct relation between them. Therefore, the understanding of the specific correlation between chemical compounds and its contributors needs further research.

## Conclusion

This study reveals the physicochemical characteristics and bacterial diversity of fermented radish in GL, PO, and PL during the fermentation process. The changes in pH values suggest that PL may facilitate the quickest fermentation of the pickles, while the process in PO progresses at the lowest rate. The PL brine samples contain higher levels of lactic acid and threonine, while more abundant volatile flavor compounds are evident in PO. The container materials had no significant influence on the microbial structure. *Lactobacillus* was the absolute dominant genus in all containers. But it had an effect on the abundance of specific genus, such as *Lactococcus* and *Pediococcus*. The correlation between these major genera was also analyzed and gene function prediction indicated that the top three pathways were carbohydrate metabolism, amino acid metabolism, and energy metabolism. *Lactobacillus* negatively correlated with methionine, tyrosine, lysine, and arginine, while it positively correlated with ammonia, and lactic acid and acetic acid both just correlated with *Pediococcus*. This study provides new insights into the microbiota succession and chemical compounds involved in vegetable fermentation.

## Data Availability Statement

All datasets generated for this study are included in the article/Supplementary Material.

## Author Contributions

LL and YR conceived and designed the experiments. XS, YQ, XC, and YT performed the experiments. XS, YL, and SG analyzed the data. GL and WX contributed reagents. LL, YR, and XS wrote the manuscript.

### Conflict of Interest

The authors declare that the research was conducted in the absence of any commercial or financial relationships that could be construed as a potential conflict of interest.

## Supplementary Material

Figure S1 Principal Component Analysis (PCA) of the 16S rRNA sequencing data.

Figure S2 Partial Least Squares Discrimination Analysis (PLS-DA) analysis of the 16S rRNA sequencing data.

Figure S3 The rarefaction curves of bacteria at phylum **(A)**, class **(B)** and genus **(C)** levels; The Shannon curves **(D)** and Simpson curves **(E)** of bacteria at genus level.

Table S1 Hardness, springiness, cohesiveness and chewiness during the fermentation process in different containers.

Table S2 Average contents of organic acid during the fermentation process in different containers.

Table S3 Average concentration of free amino acids during the fermentation process in different containers.

Table S4 Chemical compounds detected in the study.

Table S5 CLR abundance of OTU during the fermentation in different containers.
