# Supplementary figures and images for: Microbiota Succession and Chemical Composition Involved in the Radish Fermentation Process in Different Containers
Source: Front Microbiol. 2020 Apr 3;11:445. doi: 10.3389/fmicb.2020.00445 (PMC7146078; doi:10.3389/fmicb.2020.00445)

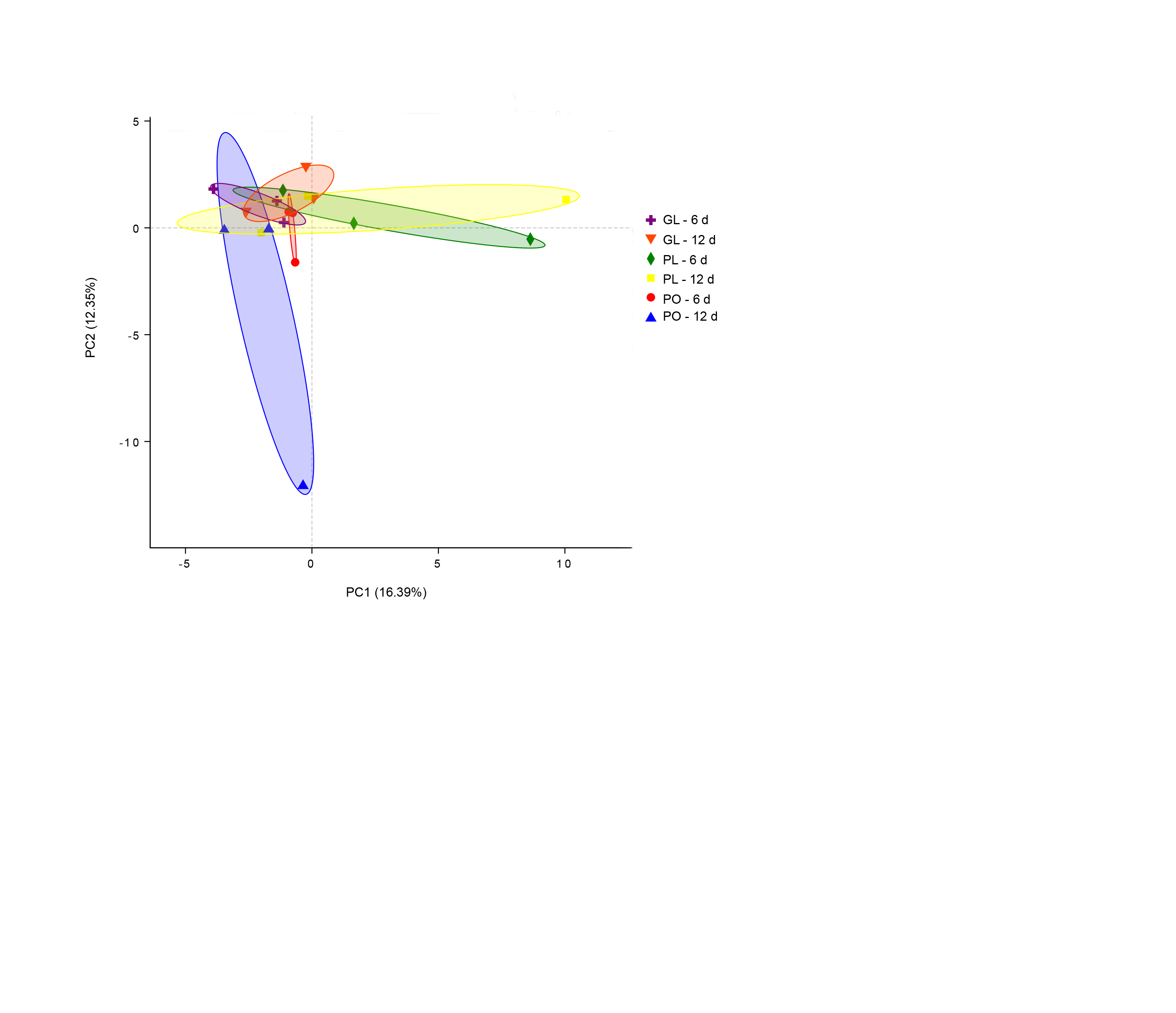

Supplement: Figure S1 — Principal Component Analysis (PCA) of the 16S rRNA sequencing data. [file Image_1.tif]

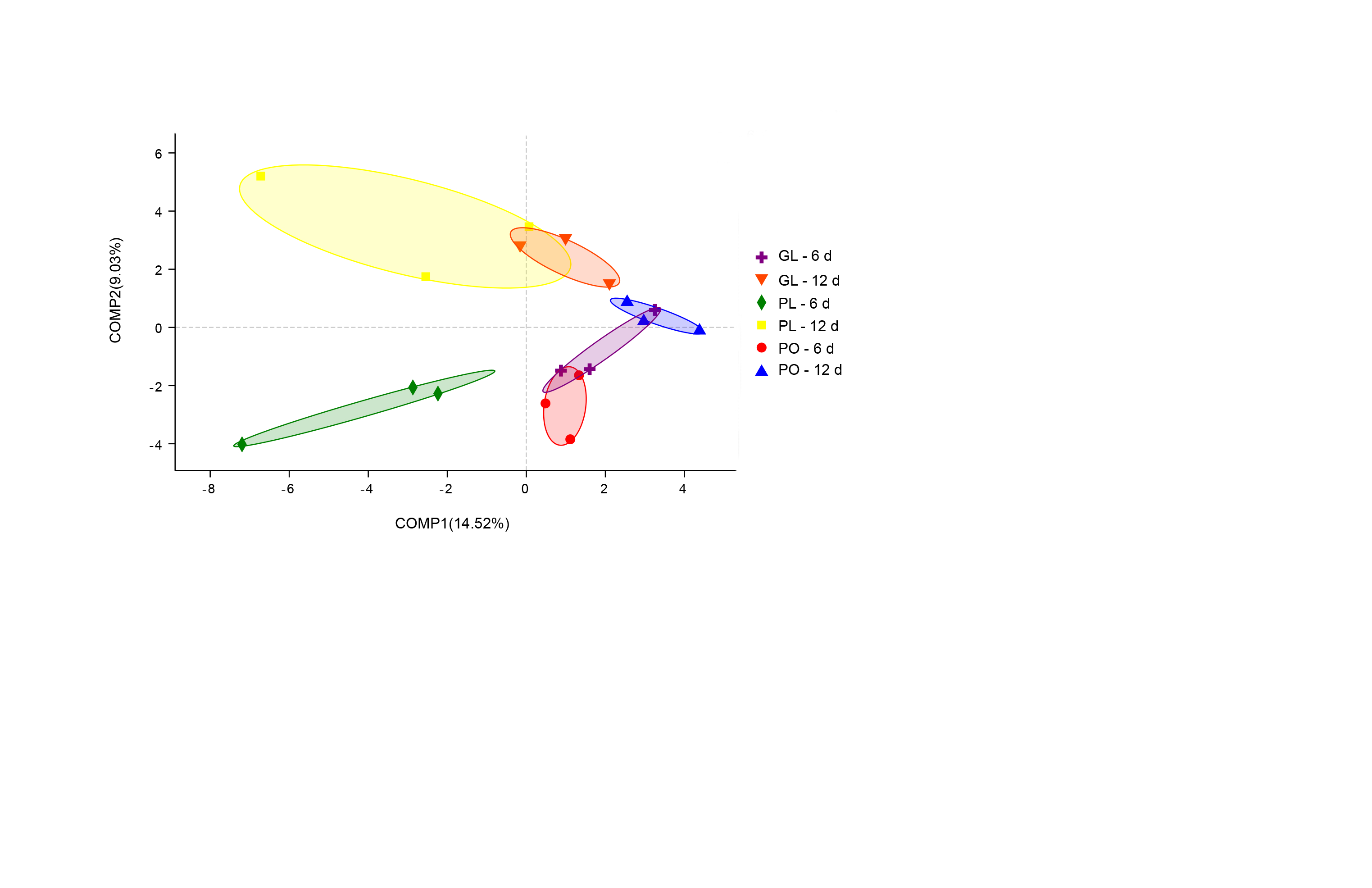

Supplement: Figure S2 — Partial Least Squares Discrimination Analysis (PLS-DA) analysis of the 16S rRNA sequencing data. [file Image_2.tif]

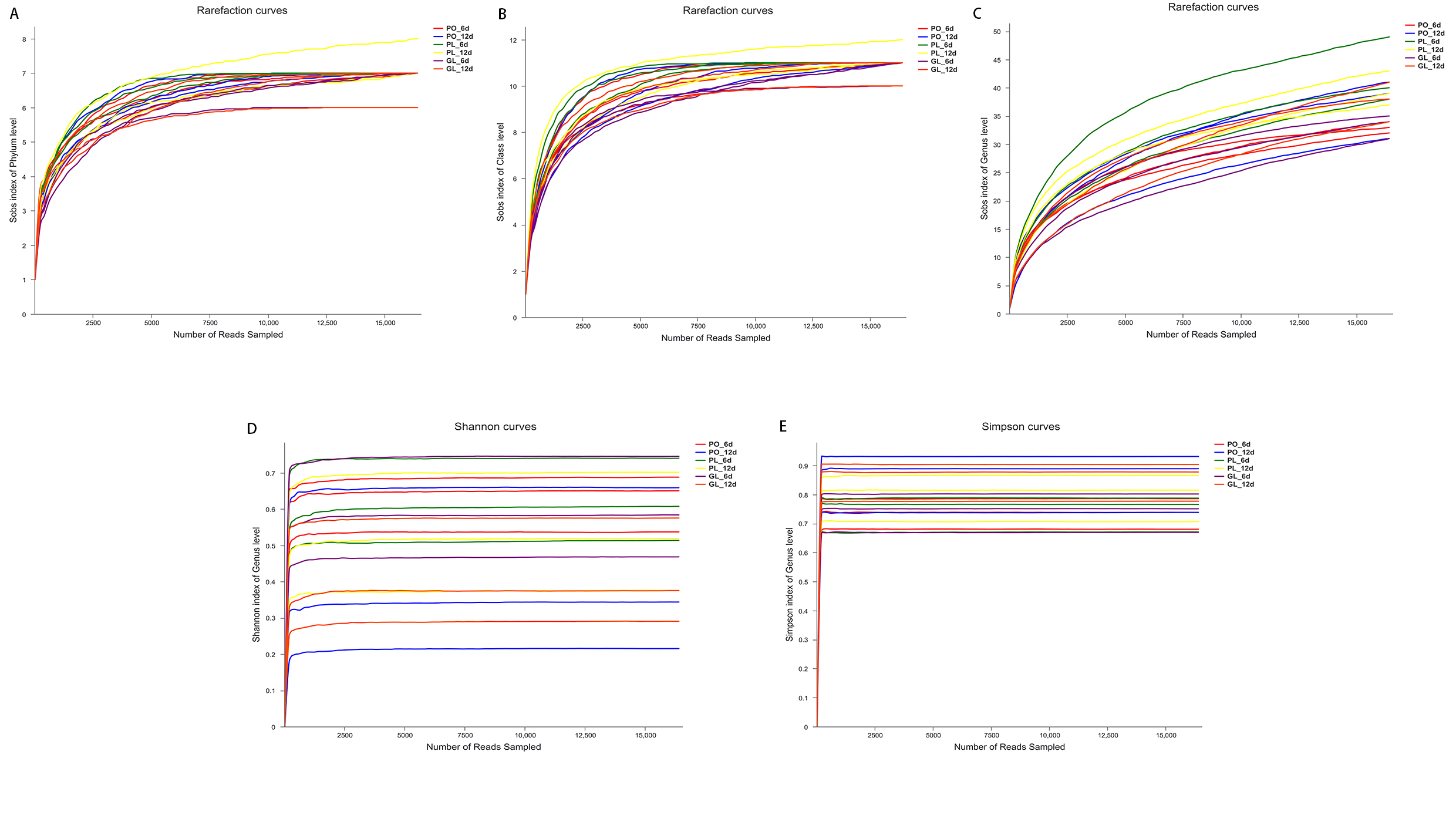

Supplement: Figure S3 — The rarefaction curves of bacteria at phylum (A), class (B) and genus (C) levels; The Shannon curves (D) and Simpson curves (E) of bacteria at genus level. [file Image_3.tif]
