# Supplementary material for: Microbiota Succession and Chemical Composition Involved in the Radish Fermentation Process in Different Containers
Source: Front Microbiol. 2020 Apr 3;11:445. doi: 10.3389/fmicb.2020.00445 (PMC7146078; doi:10.3389/fmicb.2020.00445)
Supplement: Table S1 — Hardness, springiness, cohesiveness and chewiness during the fermentation process in different containers. [file Data_Sheet_1.docx]

Table S1 Hardness, springiness, cohesiveness and chewiness during the fermentation process in different containers.

|  | Hardness (g) | Springiness | Cohesiveness | Chewiness |
| --- | --- | --- | --- | --- |
| Initial (0 d) | 3245.238^ab^+145.553 | 0.142^d^+0.007 | 0.082^c^+0.007 | 35.689^d^+9.235 |
| GL-6 d | 1399.809^d^+195.459 | 0.082^e^+0.003 | 0.049^d^+0.002 | 220.759^c^+22.140 |
| PO-6 d | 1801.443^cd^+116.976 | 0.064^e^+0.007 | 0.039^d^+0.006 | 169.001^c^+31.965 |
| PL-6 d | 1417.013^d^+144.896 | 0.075^e^+0.007 | 0.046^d^+0.008 | 181.177^c^+11.871 |
| GL-12 d | 2186.013^c^+216.586 | 0.473^c^+0.009 | 0.261^a^+0.011 | 259.339^bc^+17.778 |
| PO-12 d | 3404.296^a^+215.241 | 0.501^b^+0.013 | 0.221^b^+0.018 | 388.075^a^+42.420 |
| PL-12 d | 2940.988^b^+256.492 | 0.456^c^+0.015 | 0.227^b^+0.018 | 303.019^b^+40.751 |

Table S2 Average contents of organic acid during the fermentation process in different containers.

| Organic acid (g/L) | Oxalic acid | Lactic acid | Acetic acid | Butyric acid | α-Ketoglutaric acid |
| --- | --- | --- | --- | --- | --- |
| Initial (0 d) | nd | nd | nd | nd | nd |
| GL-6 d | 4.176^cd^+0.636 | 2.502^d^+0.287 | 0.870^d^+0.021 | nd | 0.013^c^+0.002 |
| PO-6 d | 4.808^c^+0.240 | 3.681^cd^+0.355 | 0.921^c^+0.011 | nd | 0.019^bc^+0.001 |
| PL-6 d | 4.489^cd^+0.308 | 2.757^d^+0.183 | 0.884^cd^+0.024 | nd | 0.013^c^+0.005 |
| GL-12 d | 4.467^cd^+0.464 | 4.616^c^+0.601 | 0.970^b^+0.016 | 0.235^c^+0.027 | 0.010^c^+0.001 |
| PO-12 d | 7.49^b^+0.464 | 10.890^b^+0.767 | 1.365^a^+0.026 | 0.820^b^+0.037 | 0.028^b^+0.008 |
| PL-12 d | 10.128^a^+0.315 | 18.044^a^+1.363 | 1.397^a^+0.035 | 2.074^a^+0.071 | 0.066^a^+0.012 |

Table S3 Average concentration of free amino acids during the fermentation process in different containers.

| compounds (μg/100mL) | Initial  (0 d) | GL-6 d | PO-6 d | PL-6 d | GL-12 d | PO-12 d | PL-12 d |
| --- | --- | --- | --- | --- | --- | --- | --- |
| Aspartic acid | 6.4 | 2.76 | 3.44 | 5.43 | 1.61 | 1.7 | 2.5 |
| Threonine | 54.53 | 27.04 | 62.48 | 88.88 | 22.54 | 20.53 | 28.78 |
| Serine | 5.6 | 3.62 | 5.21 | 7.2 | 3.67 | 3.07 | 3.55 |
| Glutamic acid | 4.72 | 5.28 | 5.6 | 7.24 | 5.58 | 4.26 | 5.92 |
| Glycine | 1.32 | 2.42 | 1.67 | 2.95 | 1.56 | 1.33 | 1.81 |
| Alanine | 5.78 | 7.36 | 8.27 | 11.75 | 5.65 | 4.84 | 6.69 |
| Cystine | 0.85 | 0.93 | 0.9 | 0.91 | 0.85 | 0.84 | 0.86 |
| Proline | 6.76 | 5.63 | 6.57 | 10.53 | 4.72 | 3.73 | 5.54 |
| Methionine | 1.59 | 0.77 | 1.23 | 1.18 | 0.64 | 0.75 | 0.69 |
| Isoleucine | 4.06 | 3.04 | 2.91 | 5.71 | 2.16 | 1.8 | 2.54 |
| Leucine | 3.36 | 3.71 | 2.91 | 4.69 | 2.57 | 2.28 | 2.8 |
| Tyrosine | 2.07 | 1.98 | 1.66 | 2.64 | 0.29 | 0.69 | 1.28 |
| Phenylalanine | 2.41 | 2.34 | 2.18 | 3.55 | 1.51 | 1.35 | 1.89 |
| Lysine | 4.99 | 4.08 | 4.01 | 5.4 | 2.85 | 2.25 | 3.22 |
| ammonia | 0.96 | 3.03 | 3.61 | 4.01 | 5.82 | 5.4 | 4.79 |
| Histidine | 2.54 | 1.9 | 2.52 | 3.94 | 1.76 | 1.37 | 2.14 |
| Arginine | 9.74 | 3 | 3.37 | 3.83 | 1.28 | 1.87 | 2.44 |
| Proline | 1.53 | 1.52 | 2.21 | 4.15 | 2.19 | 1.2 | 2.19 |

Table S4 Chemical compounds detected in the study.

| NO. | Compounds | CAS | GL-6-1 | GL-6-2 | GL-6-3 | GL-12-1 | GL-12-2 | GL-12-3 | PO-6-1 | PO-6-2 | PO-6-3 | PO-12-1 | PO-12-2 | PO-12-3 | PL-6-1 | PL-6-2 | PL-6-3 | PL-12-1 | PL-12-2 | PL-12-3 |
| --- | --- | --- | --- | --- | --- | --- | --- | --- | --- | --- | --- | --- | --- | --- | --- | --- | --- | --- | --- | --- |
| **Sulfur-containing compounds (μg/L)** | | | | | | | | | | | | | | | | | | | | |
| V.1 | 3-(methylthio)propyl isothiocyanate | 505-79-3 | 28.807 | 15.410 | 29.742 | 15.803 | nd | 15.971 | 105.234 | nd | nd | 3.409 | 9.153 | nd | nd | 14.843 | nd | 4.704 | nd | 1.066 |
| V.2 | 4-(methylthio)-3-butenylisothiocyanate | 51598-96-0 | nd | nd | 2.575 | nd | nd | 18.485 | 94.983 | nd | 8.516 | nd | nd | nd | 1.513 | 1.958 | 6.717 | nd | nd | 11.912 |
| V.3 | Methyl 2-thiofuroate | 13679-61-3 | nd | nd | nd | 1.036 | nd | nd | nd | nd | nd | nd | nd | nd | nd | nd | nd | nd | nd | nd |
| V.4 | Methyl thiobutyrate | 2432-51-1 | nd | nd | nd | nd | nd | nd | nd | nd | nd | nd | nd | nd | nd | nd | nd | nd | 2.788 | 5.525 |
| V.5 | Pyributicarb | 88678-67-5 | nd | nd | nd | nd | nd | nd | nd | nd | nd | nd | nd | nd | nd | nd | nd | nd | 0.741 | nd |
| V.6 | 1-nonanethiol | 1455-21-6 | nd | 5.515 | nd | nd | nd | nd | nd | nd | nd | nd | nd | nd | nd | nd | nd | nd | nd | nd |
| V.7 | 2-(benzylthio)-9-(2,3-dimethoxyphenyl)-5,6,7,9-tetrahydro-[1,2,4]triazolo[5,1-b]quinazolin-8-ol | 906765-09-1 | nd | nd | nd | nd | 13.643 | nd | nd | nd | nd | nd | nd | nd | nd | nd | nd | nd | 7.561 | nd |
| V.8 | 3-(methylthio)butanal | 16630-52-7 | nd | nd | nd | 2.176 | nd | nd | nd | nd | nd | nd | nd | nd | nd | nd | nd | nd | nd | nd |
| V.9 | Piperidine-2-thione | 13070-01-4 | 194.939 | 149.013 | 210.431 | 135.773 | 157.540 | 135.975 | 710.620 | 161.307 | 133.953 | 40.434 | 101.508 | 110.091 | 149.886 | 140.325 | 197.035 | 97.878 | 49.316 | 38.264 |
| V.10 | Pyrrolidine-2-thione | 2295-35-4 | nd | nd | nd | nd | 11.972 | nd | nd | 30.459 | 16.504 | nd | nd | 5.544 | 14.360 | nd | 20.908 | nd | nd | nd |
| V.11 | 1,1-bis(methylthio)ethane | 7379-30-8 | nd | nd | nd | nd | 3.025 | 2.823 | nd | 2.865 | nd | nd | nd | 2.285 | nd | nd | nd | nd | nd | 3.440 |
| V.12 | Thiane | 1613-51-0 | nd | nd | nd | nd | nd | nd | nd | 2.624 | nd | nd | nd | nd | nd | nd | nd | nd | nd | nd |
| V.13 | Tris(methylthio)methane | 5418-86-0 | nd | nd | nd | nd | nd | nd | nd | 10.463 | nd | nd | nd | 1.546 | nd | nd | nd | nd | nd | nd |
| V.14 | Dimethyl tetrasulfide | 5756-24-1 | 8.258 | nd | nd | nd | nd | nd | nd | nd | nd | nd | nd | nd | nd | nd | nd | nd | nd | 1.359 |
| V.15 | Sec-butylcyclohexyl sulfide | 7133-22-4 | nd | nd | nd | nd | nd | 9.249 | nd | 4.397 | nd | nd | nd | nd | nd | nd | nd | nd | nd | nd |
| V.16 | Dimethyl trisulfide | 3658-80-8 | 165.941 | 81.349 | 94.720 | 61.054 | 90.630 | 98.836 | 1158.953 | 66.500 | 69.985 | 37.464 | 71.916 | 76.826 | 161.267 | 77.018 | 84.525 | 59.645 | 71.847 | 108.407 |
| V.17 | Di-tert-dodecyl disulfide | 27458-90-8 | nd | nd | 0.911 | nd | nd | nd | nd | nd | nd | nd | nd | nd | nd | nd | nd | nd | nd | nd |
| V.18 | N,n-dimethylthioacetamide | 631-67-4 | nd | nd | nd | 0.483 | nd | nd | nd | nd | nd | nd | nd | nd | nd | nd | nd | nd | nd | nd |
| V.19 | 4-methylthiosemicarbazide | 6610-29-3 | nd | 2.892 | nd | nd | nd | nd | nd | nd | nd | nd | nd | nd | nd | nd | nd | nd | nd | nd |
| V.20 | 5-(methylthio)-valeronitrile,5-(methylthio)-pentanenitrile,1-cyano-4-(methylthio)butane,5-methylthiopentanenitrile | 59121-25-4 | nd | nd | nd | nd | nd | 16.070 | nd | nd | nd | nd | nd | nd | nd | nd | nd | nd | nd | nd |
| V.21 | 4-ethyl-3-thiosemicarbazide | 13431-34-0 | nd | nd | nd | nd | nd | nd | nd | nd | nd | nd | nd | 1.452 | nd | nd | nd | nd | nd | nd |
| **Phenols (μg/L)** | | | | | | | | | | | | | | | | | | | | |
| V.22 | 3,5-di-tert-butylphenol | 1138-52-9 | 16.756 | 16.002 | 19.118 | 14.437 | 12.052 | 15.855 | 31.408 | 5.761 | 3.505 | 0.999 | 0.954 | 3.967 | 18.637 | 9.046 | 8.891 | 9.651 | 6.500 | 3.524 |
| V.23 | 4,6-di(tert-butyl)benzene-1,3-diol | 5374-6-1 | nd | nd | nd | nd | nd | nd | 0.528 | nd | nd | nd | nd | nd | nd | nd | nd | nd | nd | nd |
| V.24 | 2,6-di-tert-butyl-4-methylphenol | 128-37-0 | nd | nd | nd | nd | nd | nd | nd | nd | nd | nd | nd | nd | nd | nd | nd | nd | nd | 0.386 |
| **Esters (μg/L)** | | | | | | | | | | | | | | | | | | | | |
| V.25 | (1-hydroxy-2,4,4-trimethylpentan-3-yl) 2-methylpropanoate | 74367-33-2 | 2.905 | 2.349 | nd | 1.483 | nd | 1.311 | nd | nd | nd | nd | nd | nd | nd | nd | nd | nd | nd | nd |
| V.26 | Dibutyl phthalate | 84-74-2 | 4.317 | 2.289 | 1.945 | 9.153 | 5.526 | 4.360 | nd | 1.247 | 1.296 | 0.803 | 0.660 | 0.592 | nd | nd | nd | nd | nd | nd |
| V.27 | Tert-butyl propiolate | 13831-03-3 | 1.385 | nd | nd | nd | nd | nd | nd | nd | nd | nd | nd | nd | nd | nd | nd | nd | nd | nd |
| V.28 | Isobornyl acetat | 80953-07-7 | 23.139 | 13.478 | 6.775 | 6.221 | 13.465 | 3.617 | 2.436 | nd | nd | 0.307 | nd | nd | nd | nd | nd | nd | nd | nd |
| V.29 | Methyl stearate | 27234-05-5 | 0.856 | nd | nd | 0.746 | nd | 0.725 | nd | nd | nd | nd | nd | nd | nd | nd | nd | nd | nd | nd |
| V.30 | Thioglycolic acid n-octyl ester | 7664-80-4 | nd | 0.722 | nd | nd | nd | nd | nd | nd | nd | nd | nd | nd | nd | nd | nd | nd | nd | nd |
| V.31 | Acetic Acid [1s,(+)]-2,7,7-Trimethyl-1α,5α-Methano-2-Cyclohexen-6β-Yl Ester | 50764-55-1 | nd | 9.458 | 11.094 | nd | 13.400 | nd | nd | nd | nd | nd | nd | nd | nd | nd | nd | nd | nd | nd |
| V.32 | 3-hydroxyoctadecanoic acid methyl ester | 420-36-2 | nd | 0.616 | nd | nd | nd | nd | nd | nd | nd | nd | nd | nd | nd | nd | nd | nd | nd | nd |
| V.33 | Diethylborinic acid 1-methyl-2-propynyl ester | 55848-32-3 | nd | 0.592 | nd | nd | nd | nd | nd | nd | nd | nd | nd | nd | nd | nd | nd | nd | nd | nd |
| V.34 | Gamma-decalactone | 706-14-9 | nd | 0.421 | nd | nd | nd | nd | nd | nd | nd | nd | nd | nd | nd | nd | nd | nd | nd | nd |
| V.35 | Rel-9-Octadecenoic Acid [(2s*)-2α*-Phenyl-1,3-Dioxolane]-4α*-Ylmethyl Ester | 56599-88-3 | nd | 0.691 | nd | nd | nd | nd | nd | nd | nd | nd | nd | nd | nd | nd | nd | nd | nd | nd |
| V.36 | Methyl 15-methylhexadecanoate | 6929-04-0 | nd | 0.765 | nd | nd | 0.363 | nd | nd | nd | nd | nd | nd | nd | nd | nd | nd | nd | nd | nd |
| V.37 | 2-ethylhexyl octanoate | 63321-70-0 | nd | nd | 0.796 | nd | 1.217 | nd | nd | nd | nd | nd | nd | nd | nd | nd | nd | nd | nd | nd |
| V.38 | Propyl laurate | 3681-78-5 | nd | nd | nd | 0.303 | nd | nd | nd | nd | nd | nd | nd | nd | nd | nd | nd | nd | nd | nd |
| V.39 | Ethyl stearate | 111-61-5 | nd | nd | nd | 0.699 | nd | nd | nd | nd | nd | nd | nd | nd | nd | nd | nd | nd | nd | nd |
| V.40 | Ethyl carbazate | 4114-31-2 | nd | nd | nd | nd | 3.242 | nd | nd | nd | nd | nd | nd | nd | nd | nd | nd | nd | nd | nd |
| V.41 | 1,2-dimethylpropyl 2-methylbutyrate | 84696-83-3 | nd | nd | nd | nd | 0.656 | nd | nd | nd | nd | nd | nd | nd | nd | nd | nd | nd | nd | nd |
| V.42 | 4,5-dimethyl-3-hydroxy-2,5-dihydrofuran-2-one | 28664-35-9 | nd | nd | nd | nd | 2.381 | nd | nd | nd | nd | nd | nd | nd | nd | nd | nd | nd | nd | nd |
| V.43 | Carbonic acid sec-butylphenyl ester | 13183-17-0 | nd | nd | nd | nd | nd | 1.703 | nd | nd | nd | nd | nd | nd | nd | nd | nd | nd | nd | nd |
| V.44 | N-butyl pentafluoropropionate | 680-28-4 | nd | nd | nd | nd | nd | nd | 15.225 | nd | nd | nd | nd | nd | nd | nd | nd | nd | nd | nd |
| V.45 | Diethyl 2,2-difluoromalonate | 680-65-9 | nd | nd | nd | nd | nd | nd | 9.027 | nd | nd | nd | nd | nd | nd | nd | nd | nd | nd | nd |
| V.46 | O-nitrophenyl n-butyrate | 2487-26-5 | nd | nd | nd | nd | nd | nd | 2.789 | nd | nd | nd | nd | nd | nd | nd | nd | nd | nd | nd |
| V.47 | Ethyl 2-cyano-3-methylcrotonate | 759-58-0 | nd | nd | nd | nd | nd | nd | 1.217 | nd | nd | nd | nd | nd | nd | nd | nd | nd | nd | nd |
| V.48 | Cis-3-hexenyl propionate | 33467-74-2 | nd | nd | nd | nd | nd | nd | 3.471 | nd | nd | nd | nd | nd | nd | nd | nd | nd | nd | nd |
| V.49 | Isoamyl butyrate | 51115-64-1 | nd | nd | nd | nd | nd | nd | 3.479 | nd | nd | nd | nd | nd | nd | nd | nd | nd | nd | nd |
| V.50 | N-octyl acrylate | 2499-59-4 | nd | nd | nd | nd | nd | nd | 13.020 | nd | nd | nd | nd | nd | nd | nd | nd | nd | nd | nd |
| V.51 | Tetrahydrofurfuryl butyrate | 2217-33-6 | nd | nd | nd | nd | nd | nd | 0.537 | nd | nd | nd | nd | nd | nd | nd | nd | nd | nd | nd |
| V.52 | Methyl 5-acetyl-2-methoxybenzoate | 39971-36-3 | nd | nd | nd | nd | nd | nd | nd | 1.174 | nd | nd | nd | nd | nd | nd | nd | nd | nd | nd |
| V.53 | Allyl isovalerate | 2835-39-4 | nd | nd | nd | nd | nd | nd | nd | 0.432 | nd | nd | nd | nd | nd | nd | nd | nd | nd | nd |
| V.54 | 2-(trimethylsilyloxy)stearic acid methyl ester | 56196-58-8 | nd | nd | nd | nd | nd | nd | nd | 5.463 | nd | nd | nd | nd | nd | nd | nd | nd | nd | nd |
| V.55 | Isobornyl acrylate | 5888-33-5 | nd | nd | nd | nd | nd | nd | nd | 0.282 | nd | nd | nd | nd | nd | nd | nd | nd | nd | nd |
| V.56 | Behenyl behenate | 17671-27-1 | nd | nd | nd | nd | nd | nd | nd | 3.486 | nd | nd | nd | nd | nd | nd | nd | nd | nd | nd |
| V.57 | Fema 3396 | 39252-03-4 | nd | nd | nd | nd | nd | nd | nd | nd | 0.900 | nd | nd | nd | nd | nd | nd | nd | nd | nd |
| V.58 | 2,2,4-trimethyl-1,3-pentanediol diisobutyrate | 6846-50-0 | nd | nd | nd | nd | nd | nd | nd | nd | 0.522 | 0.672 | nd | nd | nd | nd | nd | 0.496 | nd | 0.291 |
| V.59 | Diethyl benzamidomalonate | 16798-45-1 | nd | nd | nd | nd | nd | nd | nd | nd | nd | 0.763 | nd | nd | nd | nd | nd | nd | nd | nd |
| V.60 | Sorbic acid vinyl ester | 42739-26-4 | nd | nd | nd | nd | nd | nd | nd | nd | nd | nd | 0.150 | nd | nd | nd | nd | nd | nd | nd |
| V.61 | Prenyl caproate | 76649-22-4 | nd | nd | nd | nd | nd | nd | nd | nd | nd | nd | 0.321 | nd | nd | nd | nd | nd | nd | nd |
| V.62 | D,l-isobornyl acetate | 125-12-2 | nd | nd | nd | nd | nd | nd | nd | nd | nd | nd | 3.488 | nd | 2.902 | 2.185 | 7.567 | 5.454 | 1.959 | 2.221 |
| V.63 | Propyl caprylate | 624-13-5 | nd | nd | nd | nd | nd | nd | nd | nd | nd | nd | nd | 1.209 | nd | nd | nd | nd | nd | nd |
| V.64 | Isooctyl thioglycolate | 25103-09-7 | nd | nd | nd | nd | nd | nd | nd | nd | nd | nd | nd | nd | 1.008 | nd | nd | nd | nd | nd |
| V.65 | Methyl formate | 107-31-3 | nd | nd | nd | nd | nd | nd | nd | nd | nd | nd | nd | nd | nd | nd | nd | 0.766 | nd | nd |
| V.66 | Methyl tert-butylacetate | 10250-48-3 | nd | nd | nd | nd | nd | nd | nd | nd | nd | nd | nd | nd | nd | nd | nd | 4.427 | nd | nd |
| V.67 | Ethyl (2s)-Pyrrolidine-2-Carboxylate | 5817-26-5 | nd | nd | nd | nd | nd | nd | nd | nd | nd | nd | nd | nd | nd | nd | nd | 0.088 | nd | nd |
| V.68 | 2-o-benzylglycolic acid ethyl ester | 32122-09-1 | nd | nd | nd | nd | nd | nd | nd | nd | nd | nd | nd | nd | nd | nd | nd | nd | nd | 1.065 |
| **Alcohols (μg/L)** | | | | | | | | | | | | | | | | | | | | |
| V.69 | 2,3,4-trimethyl-1-pentanol | 6570-88-3 | nd | nd | nd | nd | nd | nd | nd | nd | nd | nd | 0.236 | nd | nd | nd | nd | nd | nd | nd |
| V.70 | Cineole | 470-82-6 | 14.398 | 6.843 | 4.676 | 3.545 | 11.208 | 3.162 | 2.601 | 0.186 | 0.294 | 0.537 | 0.838 | 0.269 | 6.677 | 5.566 | 23.977 | 31.913 | 6.698 | 2.850 |
| V.71 | Fenchol | 1632-73-1 | 0.522 | nd | nd | nd | nd | nd | nd | nd | nd | nd | nd | nd | nd | nd | nd | 6.018 | nd | nd |
| V.72 | Borneol | 507-70-0 | 5.701 | 3.958 | 1.212 | 1.116 | 4.840 | 1.273 | nd | nd | nd | nd | 0.259 | nd | 0.375 | nd | 1.505 | 1.795 | 0.306 | nd |
| V.73 | 1-nonanol | 143-08-8 | 3.796 | nd | nd | nd | 6.589 | 11.379 | nd | nd | nd | nd | 0.620 | 2.174 | nd | nd | 5.157 | 9.864 | 2.215 | 8.995 |
| V.74 | (-)-Alpha-terpineol | 10482-56-1 | 31.282 | 12.671 | nd | nd | 12.609 | nd | nd | nd | nd | nd | 0.631 | nd | 2.207 | nd | 4.153 | 5.297 | nd | nd |
| V.75 | 2-tetradecanol | 4706-81-4 | 8.837 | 9.341 | 2.116 | nd | 1.838 | 2.430 | nd | 3.478 | nd | nd | nd | nd | nd | nd | nd | 2.347 | nd | nd |
| V.76 | Cyclododecanol | 1724-39-6 | 7.684 | 6.929 | nd | 2.970 | nd | nd | nd | 2.505 | nd | nd | nd | nd | 1.474 | 1.799 | 1.533 | nd | nd | nd |
| V.77 | 1-dodecanol | 112-53-8 | 47.258 | 49.418 | nd | nd | nd | 19.888 | 36.582 | 33.217 | nd | nd | nd | nd | nd | nd | nd | 21.237 | nd | nd |
| V.78 | (E)-2,6-dimethyl-3,7-octadiene-2,6-diol | 13741-21-4 | nd | 0.401 | nd | nd | nd | nd | nd | nd | nd | nd | nd | nd | nd | nd | nd | nd | nd | nd |
| V.79 | 1,10-decanediol | 112-47-0 | nd | 6.077 | nd | nd | 6.244 | nd | nd | nd | 0.829 | nd | nd | 1.166 | nd | nd | nd | nd | nd | nd |
| V.80 | P-dioxane-2,5-dimethanol | 14236-12-5 | nd | 0.712 | nd | nd | nd | nd | nd | nd | nd | nd | nd | nd | nd | nd | nd | nd | nd | nd |
| V.81 | 2-methoxyphenylethanol | 72403-22-6 | nd | 0.702 | nd | nd | nd | nd | nd | nd | nd | nd | nd | nd | nd | nd | nd | nd | nd | nd |
| V.82 | 1,2,3,4,5-cyclopentanepentol | 56772-25-9 | nd | 0.565 | nd | nd | nd | nd | nd | nd | nd | nd | nd | nd | nd | nd | nd | nd | nd | nd |
| V.83 | 2-butyl-1-octanol | 3913-2-8 | nd | nd | 0.372 | nd | nd | nd | nd | nd | nd | nd | nd | nd | nd | 0.593 | 3.662 | nd | 0.709 | nd |
| V.84 | 1-undecanol | 112-42-5 | nd | nd | 2.364 | nd | nd | nd | nd | nd | nd | nd | nd | nd | nd | nd | nd | nd | 7.414 | nd |
| V.85 | 5-Azulenemethanol,1,2,3,3a,4,5,6,7-Octahydro-.Alpha.,.Alpha.,3,8-Tetramethyl-,[3s- | 22451-73-6 | nd | nd | 0.983 | nd | nd | nd | nd | nd | nd | nd | nd | nd | nd | nd | nd | nd | 8.123 | nd |
| V.86 | 2-hexyl-1-decanol | 2425-77-6 | nd | nd | 17.794 | 13.230 | 10.702 | nd | nd | nd | 7.587 | nd | nd | 10.407 | 9.131 | nd | nd | nd | nd | nd |
| V.87 | 2-methoxy-6-methyloxane-3,4,5-triol | 14009-07-5 | nd | nd | nd | 2.894 | nd | nd | nd | nd | nd | nd | nd | nd | nd | nd | nd | nd | nd | nd |
| V.88 | 3-methyl-2-octanol | 27644-49-1 | nd | nd | nd | 1.803 | nd | nd | nd | nd | nd | nd | nd | nd | nd | nd | nd | nd | nd | nd |
| V.89 | (2e,6e)-3,7,11-Trimethyl-9-(Phenylsulfonyl)-2,6,10-Dodecatrien-1-Ol | 57683-67-7 | nd | nd | nd | 0.624 | nd | nd | nd | nd | nd | nd | nd | nd | nd | nd | nd | nd | nd | nd |
| V.90 | Cis-alpha,alpha,5-trimethyl-5-vinyltetrahydrofuran-2-methanol | 5989-33-3 | nd | nd | nd | nd | 0.430 | nd | nd | nd | nd | nd | nd | nd | nd | nd | nd | nd | nd | nd |
| V.91 | Cis-2-pinanol | 4948-28-1 | nd | nd | nd | nd | 1.578 | nd | nd | nd | nd | nd | nd | nd | nd | nd | nd | nd | nd | nd |
| V.92 | (1s,2s,4r)-(-)-Alpha,Alpha-Dimethyl-1-Vinyl-O-Menth-8-Ene-4-Methanol | 639-99-6 | nd | nd | nd | nd | 1.034 | nd | nd | nd | nd | nd | nd | nd | nd | nd | nd | nd | nd | nd |
| V.93 | Dl-isoborneol | 124-76-5 | 2.556 | 1.795 | 0.585 | 0.870 | 3.551 | 0.405 | nd | nd | nd | nd | nd | nd | nd | nd | 1.301 | nd | nd | nd |
| V.94 | 2-methyl-1-octanol | 818-81-5 | nd | nd | nd | nd | nd | 1.363 | nd | nd | nd | nd | nd | nd | nd | nd | nd | nd | nd | nd |
| V.95 | 5-Azulenemethanol, 1,2,3,4,5,6,7,8-Octahydro-Alpha,Alpha,3,8-Tetramethyl-, [3s-(3alpha,5alpha,8alpha)]- | 489-86-1 | nd | nd | nd | nd | nd | 0.723 | nd | nd | nd | nd | nd | nd | nd | nd | nd | nd | nd | nd |
| V.96 | (S)-(+)-6-methyl-1-octanol | 110453-78-6 | nd | nd | nd | nd | nd | nd | 1.168 | nd | nd | nd | nd | nd | nd | nd | nd | nd | nd | nd |
| V.97 | Trans-2-undecen-1-ol | 75039-84-8 | nd | nd | nd | nd | nd | nd | 5.983 | nd | nd | nd | nd | nd | nd | nd | nd | nd | nd | nd |
| V.98 | 4-methyl-1-hepten-4-ol | 1186-31-8 | nd | nd | nd | nd | nd | nd | 2.734 | nd | nd | nd | nd | nd | nd | nd | nd | nd | nd | nd |
| V.99 | L-threitol, 2-o-nonyl- | 163776-15-6 | nd | nd | nd | nd | nd | nd | 2.466 | nd | nd | nd | nd | nd | 0.301 | nd | nd | nd | nd | nd |
| V.100 | 2,3-dimethyl-3-buten-2-ol | 10473-13-9 | nd | nd | nd | nd | nd | nd | nd | 0.418 | nd | nd | nd | nd | nd | nd | nd | nd | nd | nd |
| V.101 | 3,7-dimethyl-1-octanol | 151-19-9 | nd | nd | nd | nd | nd | nd | nd | 0.565 | nd | nd | nd | nd | nd | nd | nd | nd | nd | nd |
| V.102 | 2-ethyl-1-decanol | 21078-65-9 | nd | nd | nd | nd | nd | nd | nd | 0.174 | nd | nd | nd | nd | nd | nd | nd | nd | nd | nd |
| V.103 | 2,3-dimethyl-3-hexanol | 4166-46-5 | nd | nd | nd | nd | nd | nd | nd | 1.460 | nd | nd | nd | nd | nd | nd | nd | nd | nd | nd |
| V.104 | Cis-2-methylcyclohexanol | 7443-70-1 | nd | nd | nd | nd | nd | nd | nd | 0.116 | nd | nd | nd | nd | nd | nd | nd | nd | nd | nd |
| V.105 | 2-hexadecanol | 14852-31-4 | nd | nd | nd | nd | nd | nd | nd | 0.173 | nd | nd | nd | nd | nd | nd | nd | nd | nd | nd |
| V.106 | 3,4-dimethyl-2-hexanol | 19550-05-1 | nd | nd | nd | nd | nd | nd | nd | nd | 0.740 | nd | nd | nd | nd | nd | nd | nd | nd | 0.276 |
| V.107 | 6-mercaptohexan-1-ol | 1633-78-9 | nd | nd | nd | nd | nd | nd | nd | nd | nd | 0.898 | nd | nd | nd | nd | nd | nd | nd | nd |
| V.108 | (R,r)-(+)-2,4-dimethylheptan-1-ol | 18450-73-2 | nd | nd | nd | nd | nd | nd | nd | nd | nd | 0.286 | nd | nd | nd | nd | nd | nd | 0.843 | nd |
| V.109 | (-)-Myrtenol, 97 | 515-00-4 | nd | nd | nd | nd | nd | nd | nd | nd | nd | nd | 0.475 | nd | 0.539 | 0.662 | nd | nd | nd | nd |
| V.110 | 2-tridecanol | 1653-31-2 | nd | nd | nd | nd | nd | nd | nd | nd | nd | nd | 1.417 | nd | nd | nd | nd | nd | nd | nd |
| V.111 | 1-hexadecanol | 36653-82-4 | nd | nd | nd | nd | nd | nd | nd | nd | nd | nd | 7.512 | nd | nd | nd | nd | nd | nd | 1.357 |
| V.112 | 1-(2-furyl)-2-methyl-1,2-butanediol | 18927-21-4 | nd | nd | nd | nd | nd | nd | nd | nd | nd | nd | nd | 1.421 | nd | nd | nd | nd | nd | nd |
| V.113 | 3,7,11-trimethyldodecan-3-ol | 7278-65-1 | nd | nd | nd | nd | nd | nd | nd | nd | nd | nd | nd | 0.423 | nd | nd | nd | nd | nd | nd |
| V.114 | 4-methoxy-4-methyl-2-pentanol | 141-73-1 | nd | nd | nd | nd | nd | nd | nd | nd | nd | nd | nd | 0.210 | 0.393 | nd | nd | nd | nd | nd |
| V.115 | 2-decanol | 1120-06-5 | nd | nd | nd | nd | nd | nd | nd | nd | nd | nd | nd | 0.873 | nd | nd | nd | nd | nd | nd |
| V.116 | 3-methylcyclohexanol | 591-23-1 | nd | nd | nd | nd | nd | nd | nd | nd | nd | nd | nd | nd | 1.053 | nd | nd | nd | nd | nd |
| V.117 | 3,3-dimethylcyclohexan-1-ol | 767-12-4 | nd | nd | nd | nd | nd | nd | nd | nd | nd | nd | nd | nd | 0.693 | 0.419 | nd | nd | nd | nd |
| V.118 | 2-propyl-1-heptanol | 10042-59-8 | nd | nd | nd | nd | nd | nd | nd | nd | nd | nd | nd | nd | 10.951 | nd | nd | nd | nd | nd |
| V.119 | 2-dodecanol | 10203-28-8 | nd | nd | nd | nd | nd | nd | nd | nd | nd | nd | nd | nd | 0.998 | 1.164 | nd | nd | nd | nd |
| V.120 | 1-octanol | 111-87-5 | nd | nd | nd | nd | nd | nd | nd | nd | nd | nd | nd | nd | nd | 0.547 | nd | nd | nd | nd |
| V.121 | 2-methyl-1-decanol | 18675-24-6 | nd | nd | nd | nd | nd | nd | nd | nd | nd | nd | nd | nd | nd | 10.557 | nd | nd | nd | nd |
| V.122 | (1s-Exo)-1,3,3-Trimethylbicyclo[2.2.1]Heptan-2-Ol | 22627-95-8 | nd | nd | nd | nd | nd | nd | nd | nd | nd | nd | nd | nd | nd | nd | 4.857 | nd | nd | nd |
| V.123 | 1,3,5-cyclohexanetriol | 2041-15-8 | nd | nd | nd | nd | nd | nd | nd | nd | nd | nd | nd | nd | nd | nd | 1.202 | nd | nd | nd |
| V.124 | 2-(2-chloroethoxy)ethanol | 628-89-7 | nd | nd | nd | nd | nd | nd | nd | nd | nd | nd | nd | nd | nd | nd | 0.868 | nd | nd | nd |
| V.125 | Dl-2-octanol | 123-96-6 | nd | nd | nd | nd | nd | nd | nd | nd | nd | nd | nd | nd | nd | nd | 0.733 | nd | nd | nd |
| V.126 | 4-methylcyclohexanol | 589-91-3 | nd | nd | nd | nd | nd | nd | nd | nd | nd | nd | nd | nd | nd | nd | nd | 0.240 | nd | nd |
| V.127 | Cannabivarol | 33745-21-0 | nd | nd | nd | nd | nd | nd | nd | nd | nd | nd | nd | nd | nd | nd | nd | 18.178 | nd | nd |
| V.128 | Cis-2-nonen-1-ol | 22104-79-6 | nd | nd | nd | nd | nd | nd | nd | nd | nd | nd | nd | nd | nd | nd | nd | 8.770 | nd | nd |
| V.129 | 5-methyl-2-isopropyl-1-hexanol. | 2051-33-4 | nd | nd | nd | nd | nd | nd | nd | nd | nd | nd | nd | nd | nd | nd | nd | 0.734 | nd | nd |
| V.130 | Dihydroterpineol | 498-81-7 | nd | nd | nd | nd | nd | nd | nd | nd | nd | nd | nd | nd | nd | nd | nd | 0.571 | nd | nd |
| V.131 | Cyclodecanol | 1502-05-2 | nd | nd | nd | nd | nd | nd | nd | nd | nd | nd | nd | nd | nd | nd | nd | 2.830 | nd | nd |
| V.132 | 1,4-cyclohexanedimethanol | 105-08-8 | nd | nd | nd | nd | nd | nd | nd | nd | nd | nd | nd | nd | nd | nd | nd | nd | 0.606 | 0.174 |
| V.133 | L-(+)-prolinol | 23356-96-9 | nd | nd | nd | nd | nd | nd | nd | nd | nd | nd | nd | nd | nd | nd | nd | nd | 0.385 | nd |
| V.134 | Trans-2-dodecenol | 22104-81-0 | nd | nd | nd | nd | nd | nd | nd | nd | nd | nd | nd | nd | nd | nd | nd | nd | 1.048 | nd |
| V.135 | Dl-alaninol | 6168-72-5 | nd | nd | nd | nd | nd | nd | nd | nd | nd | nd | nd | nd | nd | nd | nd | nd | nd | 0.745 |
| V.136 | 6,9,12-octadecatrien-1-ol | 56630-94-5 | nd | nd | nd | nd | nd | nd | nd | nd | nd | nd | nd | nd | nd | nd | nd | nd | nd | 1.937 |
| V.137 | 3-cyclohexyl-1-propanol | 1124-63-6 | nd | nd | nd | nd | nd | nd | nd | nd | nd | nd | nd | nd | nd | nd | nd | nd | nd | 0.974 |
| V.138 | (1s,6r)-3,7,7-Trimethylbicyclo[4.1.0]Heptan-3α-Ol | 4017-79-2 | nd | nd | nd | nd | nd | nd | nd | nd | nd | nd | nd | nd | nd | nd | nd | nd | nd | 1.803 |
| V.139 | 2-ethyl-2-propyl-1-hexanol | 54461-00-6 | nd | nd | nd | nd | nd | nd | nd | nd | nd | nd | nd | nd | nd | nd | nd | nd | nd | 0.235 |
| **Aldehydes (μg/L)** | | | | | | | | | | | | | | | | | | | | |
| V.140 | 1-nonanal | 124-19-6 | nd | 1.945 | 11.772 | 20.367 | 8.143 | 27.520 | nd | 6.231 | 2.865 | 2.662 | 1.865 | 2.987 | 6.998 | 2.246 | 4.562 | 5.367 | 8.125 | 3.632 |
| V.141 | 2,4-dimethylbenzaldehyde | 15764-16-6 | nd | nd | nd | nd | nd | 31.762 | nd | nd | nd | 2.391 | nd | nd | 11.360 | nd | nd | nd | nd | nd |
| V.142 | 3,5-dimethylbenzaldehyde | 5779-95-3 | nd | nd | nd | nd | nd | nd | nd | 29.160 | nd | nd | nd | 3.034 | nd | 18.692 | 49.173 | nd | nd | nd |
| V.143 | 2,5-dimethylbenzaldehyde | 5779-94-2 | nd | 15.101 | 54.786 | 64.552 | nd | nd | nd | nd | 11.670 | nd | nd | nd | nd | nd | nd | nd | nd | nd |
| V.144 | Oleic aldehyde dimethyl acetal | 15677-71-1 | nd | nd | nd | nd | 1.132 | nd | nd | nd | nd | nd | nd | nd | nd | nd | nd | nd | nd | nd |
| V.145 | 4-(diethoxymethyl)benzaldehyde | 81172-89-6 | nd | nd | nd | nd | nd | 1.166 | nd | nd | nd | nd | nd | nd | nd | nd | nd | nd | nd | nd |
| V.146 | Fema 3766 | 17587-33-6 | nd | nd | nd | nd | nd | nd | 1.175 | nd | nd | nd | nd | nd | nd | nd | nd | nd | nd | nd |
| V.147 | Dodecyl aldehyde | 112-54-9 | nd | nd | nd | nd | nd | nd | nd | nd | nd | nd | nd | nd | nd | nd | nd | nd | nd | 0.737 |
| **Ketones (μg/L)** | | | | | | | | | | | | | | | | | | | | |
| V.148 | 2-Cyclohexyl-1-(1h-Imidazol-4-Yl)Ethanone | 69393-23-3 | nd | nd | nd | 0.955 | nd | nd | nd | nd | nd | nd | nd | nd | nd | nd | nd | nd | nd | nd |
| V.149 | 3α-(Trimethylsiloxy)-17-(Phenylmethoxyimino)-5α-Androstan-11-One | 57305-11-0 | nd | nd | nd | nd | 0.656 | nd | nd | nd | nd | nd | nd | nd | nd | nd | nd | nd | nd | nd |
| V.150 | (S)-5-(isobutyl)imidazolidine-2,4-dione | 40856-75-5 | nd | nd | nd | nd | nd | 0.500 | nd | nd | nd | nd | nd | nd | nd | nd | nd | nd | nd | nd |
| V.151 | 2,2,2-trifluoro-1-[4-(2,2,2-trifluoroacetyl)piperazin-1-yl]ethanone | 6345-81-9 | nd | nd | nd | nd | nd | nd | 29.543 | nd | nd | nd | nd | nd | nd | nd | nd | nd | nd | nd |
| V.152 | 4h-Pyran-4-One | 108-97-4 | nd | nd | nd | nd | nd | nd | nd | 0.604 | nd | nd | nd | nd | nd | nd | nd | nd | nd | nd |
| V.153 | 3-hexanone | 589-38-8 | nd | nd | nd | nd | nd | nd | nd | nd | 0.207 | nd | nd | nd | nd | nd | nd | nd | nd | nd |
| V.154 | 2-methyl-4-heptanone | 626-33-5 | nd | nd | nd | nd | nd | nd | nd | nd | nd | nd | nd | 0.140 | 1.884 | nd | nd | nd | nd | nd |
| V.155 | 3-methyl-4-heptanone | 15726-15-5 | nd | nd | nd | nd | nd | nd | nd | nd | nd | nd | nd | nd | nd | 1.264 | nd | nd | nd | nd |
| V.156 | 2,5-dimethyl-3-hexanone | 1888-57-9 | nd | nd | nd | nd | nd | nd | nd | nd | nd | nd | nd | nd | nd | 0.361 | nd | nd | nd | nd |
| V.157 | 7,9-ditert-butyl-1-oxaspiro[4.5]deca-6,9-diene-2,8-dione | 82304-66-3 | nd | nd | nd | nd | nd | nd | nd | nd | nd | nd | nd | nd | nd | nd | 4.552 | nd | nd | nd |
| V.158 | Hydroxypentanone,2-hydroxy-3-pentanone | 5704-20-1 | nd | nd | nd | nd | nd | nd | nd | nd | nd | nd | nd | nd | nd | nd | nd | nd | 1.267 | nd |
| **Alkenes (μg/L)** | | | | | | | | | | | | | | | | | | | | |
| V.159 | 1-chloro-8-heptadecene | 56554-80-4 | 0.567 | nd | nd | nd | nd | nd | nd | nd | nd | nd | nd | nd | nd | nd | nd | nd | nd | nd |
| V.160 | 2,3,4,5,6,7,8,9-Octahydro-1,1,4,4,7,7-Hexamethyl-1h-Trindene | 40650-56-4 | nd | 19.660 | 17.626 | 18.189 | 21.266 | 15.881 | nd | 5.728 | nd | nd | 5.026 | 6.953 | nd | nd | nd | nd | nd | nd |
| V.161 | (-)-Beta-citronellene | 2436-90-0 | nd | 27.171 | nd | 2.566 | nd | nd | nd | nd | nd | nd | nd | nd | nd | nd | nd | nd | nd | nd |
| V.162 | 1-hexadecene | 629-73-2 | nd | nd | nd | 0.964 | 4.628 | nd | nd | 2.046 | nd | nd | nd | nd | nd | 2.253 | 2.833 | nd | 1.891 | 0.667 |
| V.163 | (+)-Longifolene | 475-20-7 | nd | nd | nd | 0.513 | nd | nd | nd | nd | nd | nd | nd | nd | nd | nd | nd | nd | nd | nd |
| V.164 | Germacrened,1-methyl-5-methylene-8-(1-methylethyl)-1,6-cyclodecadiene | 23986-74-5 | nd | nd | nd | 0.555 | nd | nd | nd | nd | nd | nd | nd | nd | nd | nd | nd | nd | nd | nd |
| V.165 | 2,6,10,10-tetramethyl-1-oxaspiro[4.5]dec-6-ene | 36431-72-8 | nd | nd | nd | nd | 0.811 | nd | nd | nd | nd | nd | nd | nd | nd | nd | nd | nd | nd | nd |
| V.166 | (+)-Calarene | 17334-55-3 | nd | nd | nd | nd | nd | 0.422 | nd | nd | nd | nd | nd | nd | nd | nd | nd | nd | nd | nd |
| V.167 | 6,6-dimethylfulvene | 2175-91-9 | nd | nd | nd | nd | nd | nd | 2.880 | nd | nd | nd | nd | nd | nd | nd | nd | nd | nd | nd |
| V.168 | 2,3,4-trimethyl-1,4-pentadiene | 72014-90-5 | nd | nd | nd | nd | nd | nd | nd | nd | nd | 0.117 | nd | nd | nd | nd | nd | nd | nd | nd |
| V.169 | 1-chloro-2,3,3-trifluorocyclobutene | 694-62-2 | nd | nd | nd | nd | nd | nd | nd | nd | nd | nd | 1.162 | nd | nd | nd | nd | nd | nd | nd |
| V.170 | 4-acetyl-1-methyl-1-cyclohexene | 6090-09-1 | nd | nd | nd | nd | nd | nd | nd | nd | nd | nd | nd | 0.302 | nd | nd | nd | nd | nd | nd |
| V.171 | (E)-9-methyl-3-undecene | 74630-54-9 | nd | nd | nd | nd | nd | nd | nd | nd | nd | nd | nd | nd | nd | 0.388 | nd | nd | nd | nd |
| V.172 | 4,5-diethyl-3,6-dimethyl-3,5-octadiene | 61233-79-2 | nd | nd | nd | nd | nd | nd | nd | nd | nd | nd | nd | nd | nd | 4.342 | nd | nd | nd | nd |
| **Alkanes (μg/L)** | | | | | | | | | | | | | | | | | | | | |
| V.173 | 4,4-dimethyloctane | 15869-95-1 | nd | nd | nd | nd | nd | nd | nd | 1.154 | nd | nd | nd | nd | 1.928 | nd | nd | nd | nd | nd |
| V.174 | 2,3,6-trimethyloctane | 62016-33-5 | nd | nd | nd | nd | 0.974 | nd | 26.248 | nd | nd | 0.262 | nd | nd | nd | 4.567 | nd | nd | nd | 1.212 |
| V.175 | 4,5-dimethylnonane | 17302-23-7 | nd | nd | nd | nd | nd | nd | nd | nd | 0.439 | nd | nd | nd | nd | nd | 0.146 | nd | nd | nd |
| V.176 | 3,7-dimethylnonane | 72522-40-8 | nd | nd | nd | nd | nd | 3.809 | 1.219 | 0.401 | 0.227 | 0.216 | 0.365 | 0.784 | 1.013 | nd | 5.280 | nd | 0.632 | 1.479 |
| V.177 | 3,8-dimethylundecane | 17301-30-3 | 1.802 | nd | nd | 3.760 | 3.357 | nd | nd | 0.419 | 0.628 | nd | nd | nd | 0.884 | nd | nd | nd | 1.744 | nd |
| V.178 | 2,6,10-trimethyldodecane | 3891-98-3 | 6.879 | 9.294 | nd | nd | nd | 5.804 | nd | 0.242 | nd | nd | nd | nd | nd | nd | nd | nd | nd | nd |
| V.179 | 2,5,5-trimethylheptane. | 1189-99-7 | 0.482 | nd | nd | nd | nd | nd | nd | nd | nd | nd | nd | nd | nd | nd | nd | nd | nd | nd |
| V.180 | Dodecane,4,6-dimethyl | 61141-72-8 | 3.799 | 2.973 | 2.694 | 5.530 | 2.787 | 4.914 | 12.334 | 0.611 | nd | 0.937 | 1.342 | 1.829 | 3.466 | 0.935 | 4.381 | 2.693 | 3.060 | 2.443 |
| V.181 | 4-methyldodecane | 6117-97-1 | 3.534 | nd | nd | nd | nd | nd | nd | nd | nd | nd | nd | nd | nd | nd | nd | nd | nd | nd |
| V.182 | 3,6-dimethyloctane | 15869-94-0 | 9.298 | nd | nd | 9.730 | 7.467 | nd | nd | nd | 0.338 | nd | nd | nd | nd | nd | nd | nd | nd | nd |
| V.183 | 2,3,4-trimethyldecane | 62238-15-7 | 1.146 | 4.124 | nd | nd | nd | nd | nd | nd | nd | nd | nd | nd | nd | nd | nd | nd | 0.486 | nd |
| V.184 | N-heptadecane | 629-78-7 | 6.396 | 0.839 | 3.214 | 1.914 | nd | nd | nd | nd | nd | nd | nd | nd | nd | nd | nd | nd | nd | nd |
| V.185 | 2,6,11-trimethyldodecane | 31295-56-4 | 4.854 | nd | nd | 2.074 | nd | nd | nd | nd | nd | nd | nd | nd | nd | nd | nd | nd | nd | nd |
| V.186 | 5-methyl-5-propylnonane | 17312-75-3 | 4.795 | nd | nd | 3.559 | 3.178 | 0.860 | nd | nd | nd | nd | nd | nd | nd | nd | nd | nd | 0.836 | nd |
| V.187 | Tetradecane | 629-59-4 | 9.819 | 6.986 | nd | nd | nd | nd | nd | nd | 1.312 | nd | nd | nd | 0.812 | nd | nd | nd | nd | nd |
| V.188 | 4-phenyltridecane | 4534-51-4 | 4.427 | 0.545 | nd | nd | nd | nd | nd | nd | nd | nd | nd | nd | nd | 0.394 | 0.347 | nd | nd | nd |
| V.189 | 3-phenyldecane | 4621-36-7 | 2.219 | 3.103 | nd | nd | nd | nd | nd | nd | nd | nd | 0.500 | nd | nd | nd | nd | nd | nd | nd |
| V.190 | 8-propoxycedrane | 19870-75-8 | 0.565 | nd | nd | 0.911 | 0.806 | 0.670 | nd | nd | nd | nd | nd | nd | nd | nd | nd | nd | nd | nd |
| V.191 | 3-phenylundecane | 4536-87-2 | 1.919 | 2.603 | nd | nd | nd | nd | nd | nd | nd | nd | nd | nd | nd | nd | nd | nd | nd | nd |
| V.192 | 6-phenyldodecane | 2719-62-2 | 0.544 | 1.076 | nd | nd | nd | nd | nd | nd | nd | nd | nd | nd | nd | nd | nd | nd | nd | nd |
| V.193 | Heptacosane,1-chloro | 62016-79-9 | 1.114 | nd | nd | nd | nd | nd | nd | nd | nd | nd | nd | nd | nd | nd | nd | nd | nd | nd |
| V.194 | 2,5,5-trimethylheptane. | 1189-99-7 | nd | 0.950 | 0.497 | 0.361 | nd | 0.378 | nd | nd | nd | 0.175 | nd | nd | 0.278 | nd | nd | nd | nd | nd |
| V.195 | 1-chlorooctane | 111-85-3 | nd | 1.701 | nd | nd | nd | nd | nd | nd | nd | nd | nd | nd | nd | nd | nd | nd | nd | nd |
| V.196 | 1-(hexyloxy)-4-methylhexane | 74421-20-8 | nd | 0.565 | nd | nd | nd | nd | nd | nd | nd | nd | nd | nd | nd | 1.545 | nd | nd | nd | nd |
| V.197 | 5-(2-methylpropyl)nonane | 62185-53-9 | nd | 3.120 | 3.901 | 2.150 | 2.816 | nd | nd | nd | nd | nd | nd | 0.191 | nd | nd | nd | nd | nd | 0.625 |
| V.198 | Undecane,3,6-dimethyl- | 17301-28-9 | nd | 3.392 | nd | nd | 4.093 | nd | nd | nd | nd | nd | nd | nd | nd | nd | nd | nd | nd | nd |
| V.199 | 3,3,5-trimethylheptane | 7154-80-5 | nd | 1.087 | 0.791 | nd | 0.950 | nd | nd | 0.341 | nd | nd | nd | nd | nd | nd | nd | nd | nd | nd |
| V.200 | 1-iodohexadecane | 544-77-4 | nd | 2.415 | nd | 0.777 | 2.875 | nd | nd | 0.526 | nd | nd | nd | nd | nd | nd | nd | nd | nd | nd |
| V.201 | 3-ethyl-3-methylheptane | 17302-01-1 | nd | 7.114 | nd | 2.529 | 1.448 | 3.673 | nd | nd | 0.184 | 0.665 | nd | nd | nd | nd | nd | nd | 2.049 | 0.603 |
| V.202 | 1-methoxydodecane | 3482-63-1 | nd | 5.828 | nd | nd | nd | nd | nd | nd | nd | nd | nd | nd | nd | nd | nd | nd | nd | nd |
| V.203 | 4-phenyldecane | 4537-12-6 | nd | 5.113 | nd | nd | nd | nd | nd | nd | nd | nd | 0.776 | nd | nd | nd | nd | 1.170 | nd | nd |
| V.204 | 2-bromotetradecane | 74036-95-6 | nd | 0.736 | nd | nd | nd | nd | nd | nd | nd | nd | nd | nd | nd | nd | nd | nd | nd | nd |
| V.205 | 6-phenylundecane | 4537-14-8 | nd | 1.893 | nd | nd | nd | nd | nd | nd | nd | nd | nd | nd | nd | nd | nd | nd | nd | nd |
| V.206 | 5-phenyldodecane | 2719-63-3 | nd | 1.281 | nd | nd | nd | nd | nd | 0.348 | nd | nd | nd | nd | nd | nd | nd | nd | 0.506 | nd |
| V.207 | 5-phenyltridecane | 4534-50-3 | nd | 0.421 | nd | nd | nd | 0.489 | nd | nd | nd | nd | nd | 0.248 | 0.359 | nd | nd | nd | nd | nd |
| V.208 | 1-iododecane | 2050-77-3 | nd | 0.462 | nd | nd | nd | 0.333 | nd | nd | nd | nd | nd | nd | nd | nd | nd | nd | nd | nd |
| V.209 | Tridecane,1-iodo- | 35599-77-0 | nd | nd | 8.226 | nd | nd | nd | nd | 3.794 | nd | 0.162 | 1.241 | nd | nd | 2.064 | nd | nd | nd | nd |
| V.210 | N-pentadecane | 629-62-9 | nd | nd | 4.152 | 1.193 | 5.216 | 2.094 | nd | 1.005 | nd | nd | nd | nd | nd | nd | nd | nd | nd | nd |
| V.211 | Heptanal-diethylacetal | 688-82-4 | nd | nd | 1.127 | nd | nd | nd | nd | nd | nd | nd | nd | nd | nd | nd | nd | nd | nd | nd |
| V.212 | 3,4-dimethyldecane | 17312-45-7 | nd | nd | 8.030 | 7.884 | nd | 0.822 | nd | 0.404 | nd | nd | nd | nd | nd | nd | nd | nd | nd | nd |
| V.213 | N-heneicosane | 629-94-7 | nd | nd | 5.170 | nd | nd | nd | nd | nd | nd | nd | nd | nd | nd | nd | nd | nd | nd | nd |
| V.214 | 3,3,4-trimethylhexane | 16747-31-2 | nd | nd | 1.649 | nd | nd | nd | nd | nd | 0.496 | 1.214 | nd | nd | nd | nd | nd | 1.057 | 1.303 | nd |
| V.215 | (2,3-dimethyldecyl)benzene | 55134-08-2 | nd | nd | 0.695 | nd | nd | nd | nd | nd | nd | nd | nd | nd | nd | nd | nd | nd | nd | nd |
| V.216 | 4-phenyldodecane | 2719-64-4 | nd | nd | 0.666 | 0.561 | nd | nd | nd | nd | nd | nd | nd | nd | nd | nd | nd | nd | nd | nd |
| V.217 | Undecane,3,8-dimethyl- | 17301-30-3 | nd | nd | 0.506 | nd | nd | nd | nd | nd | nd | nd | nd | nd | nd | nd | nd | nd | nd | nd |
| V.218 | (2-ethyl-1-methylbutylidene)cyclohexane | 74810-41-6 | nd | nd | nd | 0.914 | nd | nd | nd | nd | nd | nd | nd | nd | nd | nd | nd | nd | nd | nd |
| V.219 | 1β,4α-Diethoxycyclohexane | 29887-72-7 | nd | nd | nd | 19.524 | nd | nd | nd | nd | nd | nd | 4.974 | nd | nd | nd | nd | nd | nd | nd |
| V.220 | 1-bromododecane | 143-15-7 | nd | nd | nd | 5.043 | nd | nd | nd | nd | nd | nd | nd | nd | nd | nd | nd | nd | nd | nd |
| V.221 | 7-methylheptadecane | 20959-33-5 | nd | nd | nd | 2.309 | 1.080 | nd | nd | nd | nd | nd | nd | nd | nd | nd | nd | nd | nd | nd |
| V.222 | 3-methylnonane | 5911-04-6 | nd | nd | nd | 4.385 | nd | nd | nd | nd | nd | nd | nd | nd | nd | nd | nd | nd | nd | nd |
| V.223 | Decane,2,3,7-trimethyl- | 62238-13-5 | nd | nd | nd | 0.894 | nd | nd | nd | nd | nd | nd | nd | nd | 0.396 | nd | nd | 1.911 | nd | nd |
| V.224 | 3,8-dimethyldecane | 17312-55-9 | nd | nd | nd | 1.524 | 0.793 | 0.547 | nd | nd | nd | nd | 0.364 | nd | nd | nd | nd | nd | nd | nd |
| V.225 | Heptadecane,2,6,10,15-tetr | 54833-48-6 | nd | nd | nd | nd | 3.289 | 1.833 | nd | nd | nd | nd | nd | nd | nd | nd | nd | nd | nd | nd |
| V.226 | 3-methyldecane | 13151-34-3 | nd | nd | nd | nd | 2.391 | nd | nd | 0.222 | 0.227 | nd | nd | nd | nd | nd | nd | nd | nd | nd |
| V.227 | 2,2,2',2'-tetramethyl-1,1'-bicyclopropane | 68998-20-9 | nd | nd | nd | nd | 1.289 | nd | nd | nd | nd | nd | nd | nd | nd | nd | nd | nd | nd | nd |
| V.228 | 1-fluorononane | 463-18-3 | nd | nd | nd | nd | 5.886 | nd | nd | nd | nd | nd | nd | nd | nd | nd | nd | nd | nd | nd |
| V.229 | 1,2-epoxycyclododecane | 286-99-7 | nd | nd | nd | nd | 2.375 | nd | nd | nd | nd | nd | nd | nd | nd | nd | nd | nd | nd | nd |
| V.230 | 1-chlorotetradecane | 2425-54-9 | nd | nd | nd | nd | 0.797 | nd | nd | nd | nd | nd | nd | nd | nd | nd | nd | 3.256 | 1.589 | nd |
| V.231 | 3,6-dimethyldecane | 17312-53-7 | nd | nd | nd | nd | nd | 3.200 | nd | nd | nd | nd | nd | nd | nd | nd | nd | nd | nd | nd |
| V.232 | Nonane,5-(1-methylpropyl)- | 62185-54-0 | nd | nd | nd | nd | nd | 3.445 | nd | nd | nd | nd | nd | nd | nd | nd | nd | nd | nd | nd |
| V.233 | 1-bromohexadecane | 112-82-3 | nd | nd | nd | nd | nd | 5.553 | nd | nd | nd | nd | nd | nd | nd | nd | nd | nd | nd | nd |
| V.234 | 1-chlorooctadecane | 3386-33-2 | nd | nd | nd | nd | nd | 2.441 | nd | nd | nd | nd | nd | nd | nd | nd | nd | nd | nd | nd |
| V.235 | 2-bromododecane | 13187-99-0 | nd | nd | nd | nd | nd | 0.503 | nd | nd | nd | nd | nd | nd | nd | nd | nd | nd | nd | nd |
| V.236 | 2,5,5-trimethylheptane. | 1189-99-7 | nd | nd | nd | nd | nd | nd | 3.031 | nd | nd | nd | nd | nd | nd | nd | nd | nd | nd | nd |
| V.237 | 3,7-dimethyldecane | 17312-54-8 | nd | nd | nd | nd | nd | nd | 10.211 | 1.149 | 0.469 | 0.197 | 0.974 | 1.034 | 4.958 | 4.983 | 4.101 | 4.524 | 4.972 | 1.341 |
| V.238 | 2-methylundecane | 97659-99-9 | nd | nd | nd | nd | nd | nd | 3.797 | nd | nd | nd | nd | nd | nd | nd | nd | nd | nd | nd |
| V.239 | 2-bromononane | 2216-35-5 | nd | nd | nd | nd | nd | nd | 10.462 | nd | nd | nd | nd | nd | nd | nd | nd | nd | nd | nd |
| V.240 | 3,4,5,6-tetramethyloctane | 62185-21-1 | nd | nd | nd | nd | nd | nd | 11.577 | nd | nd | nd | nd | nd | 0.581 | nd | nd | nd | nd | nd |
| V.241 | 4-methyl-3,5-dioxatridecane | 54889-49-5 | nd | nd | nd | nd | nd | nd | 2.917 | nd | nd | nd | nd | nd | nd | nd | nd | nd | nd | nd |
| V.242 | 2,3,6-trimethyloctane | 62016-33-5 | nd | nd | nd | nd | nd | nd | nd | 2.141 | nd | nd | nd | nd | nd | nd | nd | nd | nd | nd |
| V.243 | N-heptacosane | 593-49-7 | nd | nd | nd | nd | nd | nd | nd | 0.544 | nd | nd | nd | nd | nd | nd | nd | nd | nd | nd |
| V.244 | 3-bromodecane | 30571-71-2 | nd | nd | nd | nd | nd | nd | nd | 0.105 | nd | nd | nd | nd | nd | nd | nd | nd | nd | nd |
| V.245 | 4,4-dimethyl heptane | 1068-19-5 | nd | nd | nd | nd | nd | nd | nd | 0.213 | nd | nd | nd | nd | nd | nd | nd | nd | nd | nd |
| V.246 | 2,2,3-trimethylbutane | 464-06-2 | nd | nd | nd | nd | nd | nd | nd | nd | 0.193 | nd | nd | nd | nd | nd | nd | nd | nd | nd |
| V.247 | 2,5,5-trimethylheptane. | 1189-99-7 | nd | nd | nd | nd | nd | nd | nd | nd | 0.271 | nd | nd | nd | nd | nd | nd | nd | nd | nd |
| V.248 | 2,3,4-trimethylhexane | 921-47-1 | nd | nd | nd | nd | nd | nd | nd | nd | 0.380 | 0.444 | nd | 0.386 | nd | nd | nd | nd | nd | nd |
| V.249 | 2,2,3,4-tetramethylpentane | 1186-53-4 | nd | nd | nd | nd | nd | nd | nd | nd | 1.814 | nd | nd | 1.400 | nd | nd | nd | nd | nd | nd |
| V.250 | Heptadecane,2,6,10,15-tetr | 54833-48-6 | nd | nd | nd | nd | nd | nd | nd | nd | 0.211 | nd | nd | nd | nd | nd | nd | nd | nd | nd |
| V.251 | 4,5-dimethylnonane | 17302-23-7 | nd | nd | nd | nd | nd | nd | nd | nd | nd | 0.273 | nd | nd | nd | nd | nd | nd | nd | nd |
| V.252 | 2-hydroxymethyl-1,3-dioxolane | 5694-68-8 | nd | nd | nd | nd | nd | nd | nd | nd | nd | 0.544 | nd | nd | nd | nd | nd | nd | nd | nd |
| V.253 | N-tetradecylcyclohexane | 1795-18-2 | nd | nd | nd | nd | nd | nd | nd | nd | nd | 0.114 | nd | nd | nd | nd | nd | nd | nd | nd |
| V.254 | (1r,5r,6r)-6-Methyl-2-Methylene-6-(4-Methyl-3-Pentenyl)Bicyclo[3.1.1]Heptane | 55123-21-2 | nd | nd | nd | nd | nd | nd | nd | nd | nd | 0.726 | nd | nd | nd | nd | nd | nd | nd | nd |
| V.255 | Heptadecane,2,6,10,15-tetr | 54833-48-6 | nd | nd | nd | nd | nd | nd | nd | nd | nd | 1.645 | nd | nd | nd | nd | nd | nd | nd | nd |
| V.256 | 3,3-dimethyloctane | 4110-44-5 | nd | nd | nd | nd | nd | nd | nd | nd | nd | 0.272 | 0.292 | nd | 0.944 | 0.706 | 0.932 | 0.886 | 0.303 | nd |
| V.257 | 2,3,7-trimethyloctane | 62016-34-6 | nd | nd | nd | nd | nd | nd | nd | nd | nd | nd | 2.449 | nd | 5.362 | nd | nd | nd | 3.786 | nd |
| V.258 | 3-ethyl-3-methyldecane | 17312-66-2 | nd | nd | nd | nd | nd | nd | nd | nd | nd | nd | 0.739 | nd | nd | nd | nd | nd | nd | nd |
| V.259 | 2,4,4-trimethylhexane | 16747-30-1 | nd | nd | nd | nd | nd | nd | nd | nd | nd | nd | 1.192 | 0.361 | 1.936 | 0.925 | 1.745 | nd | nd | 0.474 |
| V.260 | Dodecane | 112-40-3 | nd | nd | nd | nd | nd | nd | nd | nd | nd | nd | 1.903 | nd | 1.431 | 1.649 | 2.611 | 1.847 | nd | nd |
| V.261 | 1,2-epoxyhexadecane | 7320-37-8 | nd | nd | nd | nd | nd | nd | nd | nd | nd | nd | 1.632 | nd | nd | nd | nd | nd | nd | nd |
| V.262 | 2-(3-bromo-but-3-enyl)-[1,3]dioxolane | 333961-98-1 | nd | nd | nd | nd | nd | nd | nd | nd | nd | nd | 0.435 | nd | nd | nd | nd | nd | nd | nd |
| V.263 | 2-allylbicyclo[2.2.1]heptane | 2633-80-9 | nd | nd | nd | nd | nd | nd | nd | nd | nd | nd | nd | 0.147 | nd | nd | nd | nd | nd | nd |
| V.264 | 2,2,3,3-tetramethylpentane | 7154-79-2 | nd | nd | nd | nd | nd | nd | nd | nd | nd | nd | nd | 0.268 | nd | nd | nd | nd | nd | nd |
| V.265 | 2,6,11-trimethyldodecane | 31295-56-4 | nd | nd | nd | nd | nd | nd | nd | nd | nd | nd | nd | 0.448 | nd | nd | nd | nd | nd | nd |
| V.266 | 3,4-dimethylheptane | 922-28-1 | nd | nd | nd | nd | nd | nd | nd | nd | nd | nd | nd | 0.225 | nd | nd | nd | nd | nd | nd |
| V.267 | 4,5-dimethyloctane | 15869-96-2 | nd | nd | nd | nd | nd | nd | nd | nd | nd | nd | nd | 0.998 | nd | nd | nd | nd | nd | nd |
| V.268 | Undecane,3,5-dimethyl- | 17312-81-1 | nd | nd | nd | nd | nd | nd | nd | nd | nd | nd | nd | nd | 0.274 | nd | nd | nd | nd | nd |
| V.269 | 1-chlorohexadecane | 4860-03-1 | nd | nd | nd | nd | nd | nd | nd | nd | nd | nd | nd | nd | 1.576 | 0.467 | nd | 0.457 | 1.580 | nd |
| V.270 | 1-(hexyloxy)-3-methylhexane | 74421-18-4 | nd | nd | nd | nd | nd | nd | nd | nd | nd | nd | nd | nd | 0.457 | nd | nd | nd | nd | nd |
| V.271 | 1-bromoundecane | 693-67-4 | nd | nd | nd | nd | nd | nd | nd | nd | nd | nd | nd | nd | 3.710 | nd | nd | nd | nd | nd |
| V.272 | 1-phenyl-3,3-dimethylbutane | 17314-92-0 | nd | nd | nd | nd | nd | nd | nd | nd | nd | nd | nd | nd | nd | 0.345 | nd | nd | nd | nd |
| V.273 | 1-(2-chloroethoxy)-1-ethoxyethane | 14689-96-4 | nd | nd | nd | nd | nd | nd | nd | nd | nd | nd | nd | nd | nd | 1.637 | nd | nd | nd | nd |
| V.274 | N-octacosane | 630-02-4 | nd | nd | nd | nd | nd | nd | nd | nd | nd | nd | nd | nd | nd | nd | 1.271 | nd | nd | nd |
| V.275 | 2,6,11-trimethyldodecane | 31295-56-4 | nd | nd | nd | nd | nd | nd | nd | nd | nd | nd | nd | nd | nd | nd | 1.658 | nd | nd | nd |
| V.276 | N-nonadecane | 629-92-5 | nd | nd | nd | nd | nd | nd | nd | nd | nd | nd | nd | nd | nd | nd | nd | 0.924 | nd | 1.235 |
| V.277 | 3-phenyldodecane | 2400-00-2 | nd | nd | nd | nd | nd | nd | nd | nd | nd | nd | nd | nd | nd | nd | nd | 0.412 | nd | 0.247 |
| V.278 | 5-ethyl-2-methylheptane | 13475-78-0 | nd | nd | nd | nd | nd | nd | nd | nd | nd | nd | nd | nd | nd | nd | nd | 0.746 | nd | nd |
| V.279 | 2,2,3,3-tetramethylhexane | 13475-81-5 | nd | nd | nd | nd | nd | nd | nd | nd | nd | nd | nd | nd | nd | nd | nd | 1.172 | nd | nd |
| V.280 | 2,6,10-trimethyldodecane | 3891-98-3 | nd | nd | nd | nd | nd | nd | nd | nd | nd | nd | nd | nd | nd | nd | nd | 4.048 | nd | nd |
| V.281 | 3-methyldodecane | 17312-57-1 | nd | nd | nd | nd | nd | nd | nd | nd | nd | nd | nd | nd | nd | nd | nd | 0.355 | nd | nd |
| V.282 | 3,4-dimethylundecane | 17312-78-6 | nd | nd | nd | nd | nd | nd | nd | nd | nd | nd | nd | nd | nd | nd | nd | nd | 0.257 | nd |
| V.283 | 3,3,4-trimethylheptane. | 20278-87-9 | nd | nd | nd | nd | nd | nd | nd | nd | nd | nd | nd | nd | nd | nd | nd | nd | 0.545 | nd |
| V.284 | Undecane,3,7-dimethyl- | 17301-29-0 | nd | nd | nd | nd | nd | nd | nd | nd | nd | nd | nd | nd | nd | nd | nd | nd | nd | 1.638 |
| V.285 | Pentane, 2-methoxy-2,4,4-trimethyl- | 62108-41-2 | nd | nd | nd | nd | nd | nd | 4.105 | nd | nd | nd | nd | nd | nd | nd | nd | nd | nd | nd |
| **Acids (μg/L)** | | | | | | | | | | | | | | | | | | | | |
| V.286 | 2-amino-5-methylbenzoic acid | 2941-78-8 | 13.196 | nd | nd | 4.002 | nd | 6.481 | nd | nd | 3.667 | 2.347 | 2.908 | nd | nd | nd | 4.025 | 6.295 | 5.597 | nd |
| V.287 | 2-hydroxytetradecanoic acid | 2507-55-3 | 17.706 | nd | 10.719 | nd | 5.937 | 4.990 | nd | nd | nd | nd | nd | nd | 4.118 | 4.017 | 7.686 | 4.959 | 2.516 | 3.857 |
| V.288 | Propanoicacid,2-methyl-, | 79-31-2 | 3.198 | 2.805 | 0.916 | 0.553 | 0.380 | nd | 8.247 | 1.082 | 2.762 | 3.081 | nd | 1.268 | 0.486 | 0.675 | 0.492 | nd | nd | nd |
| V.289 | 3-methyldecanoic acid | 60308-82-9 | nd | nd | nd | 6.796 | nd | nd | nd | nd | nd | nd | nd | nd | nd | 1.485 | nd | nd | nd | nd |
| V.290 | Hydroxylamine, o-(carboxymethyl)- | 645-88-5 | nd | nd | nd | nd | 0.891 | nd | nd | nd | nd | nd | nd | nd | nd | nd | nd | nd | nd | nd |
| V.291 | Nonanoic acid | 112-05-0 | nd | nd | nd | nd | nd | 2.638 | nd | nd | nd | nd | nd | nd | nd | nd | nd | nd | nd | nd |
| V.292 | N-formylglycine | 2491-15-8 | nd | nd | nd | nd | nd | nd | nd | nd | 0.215 | nd | nd | nd | nd | nd | nd | nd | nd | nd |
| V.293 | Propanoicacid,2-methyl-,3-hydrox | 74367-34-3 | nd | nd | nd | nd | nd | nd | nd | nd | nd | 0.376 | nd | nd | nd | nd | nd | nd | nd | nd |
| V.294 | 2-hydroxydecanoic acid | 37639-47-7 | nd | nd | nd | nd | nd | nd | nd | nd | nd | nd | 2.128 | nd | nd | nd | nd | nd | nd | nd |
| V.295 | N-cbz-l-methionine | 1152-62-1 | nd | nd | nd | nd | nd | nd | nd | nd | nd | nd | 1.623 | nd | nd | nd | nd | nd | nd | nd |
| V.296 | 1,2,4-benzenetricarboxylic acid | 528-44-9 | nd | nd | nd | nd | nd | nd | nd | nd | nd | nd | nd | nd | nd | nd | nd | nd | 1.273 | nd |
| V.297 | Haloxazolam | 59128-97-1 | nd | nd | nd | nd | nd | nd | nd | nd | nd | nd | nd | nd | nd | nd | nd | nd | 3.652 | nd |
| V.298 | Valeric acid | 109-52-4 | nd | nd | nd | nd | nd | nd | nd | nd | nd | nd | nd | nd | nd | nd | nd | nd | 0.254 | nd |
| V.299 | P-toluenesulfonic acid n-hexyl ester | 3839-35-8 | nd | nd | nd | nd | nd | nd | nd | nd | nd | nd | nd | nd | nd | nd | nd | nd | nd | 0.300 |
| **Ethers (μg/L)** | | | | | | | | | | | | | | | | | | | | |
| V.300 | 1-methoxydecane | 7289-52-3 | 3.687 | nd | nd | nd | nd | nd | nd | nd | nd | nd | 0.498 | nd | nd | nd | nd | 0.592 | nd | nd |
| V.301 | Glycidyl hexadecyl ether | 15965-99-8 | 1.488 | nd | nd | nd | nd | nd | nd | nd | nd | nd | nd | nd | nd | nd | nd | nd | nd | nd |
| V.302 | Octadecyl vinyl ether | 930-02-9 | nd | 1.634 | nd | nd | nd | nd | nd | nd | nd | nd | nd | nd | nd | nd | nd | nd | nd | nd |
| V.303 | 2-chloroethyl(1-methylundecyl) ether | 17648-35-0 | nd | nd | nd | nd | 6.380 | 12.125 | nd | nd | nd | nd | nd | 0.535 | nd | nd | nd | nd | nd | nd |
| V.304 | Methyl 3-methylbutyl ether | 626-91-5 | nd | nd | nd | nd | nd | nd | 0.845 | nd | nd | nd | nd | nd | nd | nd | nd | nd | nd | 0.351 |
| V.305 | N-hexyl n-octyl ether | 17071-54-4 | nd | nd | nd | nd | nd | nd | nd | 0.363 | nd | nd | nd | nd | nd | nd | nd | nd | nd | nd |
| V.306 | Tert-butyl methyl ether | 1634-04-4 | nd | nd | nd | nd | nd | nd | nd | nd | 0.407 | nd | nd | nd | nd | nd | nd | nd | nd | nd |
| V.307 | Cyclohexylethylether | 932-92-3 | nd | nd | nd | nd | nd | nd | nd | nd | nd | 0.096 | nd | nd | nd | nd | nd | 0.348 | nd | nd |
| V.308 | Dioctyl ether | 629-82-3 | nd | nd | nd | nd | nd | nd | nd | nd | nd | nd | nd | nd | nd | nd | 0.937 | nd | nd | nd |
| V.309 | Ethyl phenethyl ether | 1817-90-9 | nd | nd | nd | nd | nd | nd | nd | nd | nd | nd | nd | nd | nd | nd | 0.728 | nd | nd | nd |
| V.310 | 2-chloro-1,1,2-trifluoroethyl methyl ether | 425-87-6 | nd | nd | nd | nd | nd | nd | nd | nd | nd | nd | nd | nd | nd | nd | nd | nd | 0.200 | nd |
| **Arenes (μg/L)** | | | | | | | | | | | | | | | | | | | | |
| V.311 | Benzene,(1-butylhexyl)- | 963455 | 6.942 | 7.114 | nd | nd | nd | nd | nd | nd | nd | nd | nd | nd | nd | nd | nd | 2.443 | nd | nd |
| V.312 | Benzene,(1-butylheptyl)- | 4537-15-9 | 2.569 | 6.563 | 1.124 | 1.215 | 0.990 | nd | nd | nd | nd | nd | 1.219 | nd | nd | 1.105 | nd | 2.080 | nd | 0.692 |
| V.313 | Benzene,(1-propyloctyl)- | 4536-86-1 | 3.880 | 4.186 | nd | nd | nd | nd | nd | nd | nd | nd | 0.593 | nd | nd | nd | nd | 1.061 | nd | 0.202 |
| V.314 | 1-methylnonadecylbenzene | 2398-66-5 | 2.037 | nd | nd | nd | nd | nd | nd | nd | nd | nd | nd | nd | nd | nd | nd | nd | nd | nd |
| V.315 | (1,3,3-trimethylnonyl)benzene | 54986-44-6 | nd | 5.466 | nd | nd | nd | nd | nd | nd | nd | nd | nd | nd | nd | nd | nd | nd | nd | nd |
| V.316 | 1-pentyloctylbenzene | 4534-49-0 | nd | 0.405 | nd | 0.367 | nd | nd | nd | nd | nd | nd | 0.391 | nd | nd | nd | nd | nd | nd | nd |
| V.317 | P-xylen | 106-42-3 | nd | 2.379 | nd | nd | 2.060 | 2.540 | nd | nd | nd | nd | nd | nd | nd | nd | nd | nd | nd | nd |
| V.318 | (3,3-dimethyldecyl)benzene | 55134-09-3 | nd | nd | nd | 3.301 | nd | nd | nd | nd | nd | nd | nd | nd | nd | nd | nd | nd | nd | nd |
| V.319 | (2,3-dimethyldecyl)benzene | 55134-08-2 | nd | nd | nd | 0.475 | nd | nd | nd | nd | nd | nd | nd | nd | nd | nd | nd | nd | nd | nd |
| V.320 | (2,3-dimethyldecyl)benzene | 55134-08-2 | nd | nd | nd | nd | 2.417 | nd | nd | nd | nd | nd | nd | nd | nd | nd | nd | nd | nd | nd |
| V.321 | (3,3-dimethyldecyl)benzene | 55134-09-3 | nd | nd | 3.088 | nd | nd | nd | nd | nd | nd | nd | nd | nd | nd | nd | nd | nd | nd | nd |
| V.322 | (1-ethyloctadecyl)benzene | 2400-2-4 | nd | nd | 0.977 | 0.490 | nd | nd | nd | nd | nd | nd | nd | nd | nd | nd | nd | nd | nd | nd |
| V.323 | Benzene,(1-methylundecyl)- | 2719-61-1 | nd | nd | 1.205 | 1.655 | 3.587 | 0.600 | 2.993 | 0.468 | 0.312 | nd | 0.309 | 0.507 | 0.823 | 0.641 | 0.390 | 0.712 | nd | 0.310 |
| V.324 | [2-methyl-1-isopropylpropyl]benzene | 21777-84-4 | nd | nd | nd | nd | nd | nd | nd | 0.289 | nd | nd | nd | nd | nd | nd | nd | nd | nd | nd |
| V.325 | M-xylene | 108-38-3 | nd | nd | nd | nd | nd | nd | nd | 0.495 | nd | nd | nd | nd | nd | nd | nd | nd | nd | nd |
| V.326 | 5-(2-methylpropyl)nonane | 62185-53-9 | nd | nd | nd | nd | nd | 2.255 | nd | nd | nd | nd | nd | nd | nd | nd | nd | nd | nd | nd |
| V.327 | (1,1-diethylpropyl)benzene | 4170-84-7 | nd | nd | nd | nd | nd | nd | nd | nd | 0.183 | nd | nd | nd | nd | nd | nd | nd | nd | nd |
| V.328 | (1-hexadecylheptadecyl)benzene | 55517-74-3 | nd | nd | nd | 0.340 | nd | nd | nd | nd | nd | nd | nd | nd | nd | nd | nd | nd | nd | nd |
| V.329 | (1-butylhexadecyl)benzene. | 2400-04-6 | nd | nd | nd | nd | nd | nd | nd | nd | nd | nd | nd | nd | 0.266 | 1.682 | 1.077 | nd | nd | 0.764 |
| V.330 | P-xylene | 106-42-3 | nd | nd | nd | nd | nd | nd | nd | nd | nd | nd | nd | nd | nd | nd | nd | 0.835 | nd | 0.603 |
|  | 呋喃 |  |  |  |  |  |  |  |  |  |  |  |  |  |  |  |  |  |  |  |
| V.331 | 3-(methylthio)propylamine | 4104-45-4 | nd | nd | nd | nd | nd | nd | nd | 0.122 | nd | nd | nd | nd | nd | nd | nd | nd | nd | nd |
| V.332 | 2,5-dimethyltetrahydrofuran | 1003-38-9 | nd | nd | nd | nd | nd | nd | nd | nd | nd | 0.108 | nd | nd | nd | nd | nd | nd | nd | nd |
| V.333 | 2-ethyl-5-methyl furan | 1703-52-2 | nd | nd | nd | nd | nd | nd | nd | nd | nd | nd | 0.195 | nd | nd | nd | nd | nd | nd | nd |
| **Amines (μg/L)** | | | | | | | | | | | | | | | | | | | | |
| V.334 | N-(5-chloro-2-hydroxyphenyl)dodecanamide | 72298-74-9 | nd | nd | nd | nd | 0.407 | nd | nd | nd | nd | nd | nd | nd | nd | nd | nd | nd | nd | nd |
| V.335 | N-[(pentafluorophenyl)methylene]-β,3,4-tris(trimethylsilyloxy)benzeneethanamine | [55429-13-5](https://www.chemicalbook.com/Search_EN.aspx?keyword=55429-13-5) | nd | nd | nd | nd | nd | nd | 99.836 | nd | nd | nd | nd | nd | nd | nd | nd | nd | nd | nd |
| V.336 | 1,1,3,3,5,5,7,7,9,9,11,11,13,13,15,15-hexadecamethyloctasilo | 19095-24-0 | nd | nd | nd | nd | nd | nd | 12.065 | nd | nd | nd | nd | nd | 1.116 | nd | nd | nd | nd | nd |
| V.337 | Ammelide | 645-93-2 | nd | nd | nd | nd | nd | nd | nd | 0.047 | nd | nd | nd | nd | nd | nd | nd | nd | nd | nd |
| V.338 | 5-amino-3-methylisoxazole | 14678-02-5 | nd | nd | nd | nd | nd | nd | nd | nd | nd | 1.238 | nd | nd | nd | nd | nd | nd | nd | nd |
| **Miscellaneous (μg/L)** | | | | | | | | | | | | | | | | | | | | |
| V.339 | 1,2,3,6-tetrahydropyridine | 694-05-3 | nd | nd | nd | nd | nd | nd | 22.797 | nd | nd | nd | nd | nd | nd | nd | nd | nd | nd | nd |
| V.340 | 4-hydroxy-6-aminopyrimidine | 1193-22-2 | nd | 2.123 | nd | nd | nd | nd | nd | nd | nd | nd | nd | nd | nd | nd | nd | nd | nd | nd |
| V.341 | 1,4-cineole | 470-67-7 | 0.908 | nd | nd | nd | 2.562 | nd | nd | nd | nd | nd | nd | nd | nd | nd | 2.489 | 4.405 | nd | nd |
| V.342 | D-camphor | 464-49-3 | 13.088 | nd | nd | nd | 12.637 | nd | nd | nd | nd | nd | nd | nd | nd | nd | nd | nd | nd | nd |
| V.343 | 2-pentadecyl-1,3-dioxolane | 4360-57-0 | 0.554 | nd | nd | 0.477 | nd | nd | nd | 0.438 | nd | 0.383 | nd | nd | nd | nd | nd | nd | nd | nd |
| V.344 | 4-octadecylmorpholine | 16528-77-1 | 0.745 | nd | 0.495 | nd | 0.435 | nd | nd | nd | nd | nd | nd | nd | nd | nd | nd | nd | nd | nd |
| V.345 | Biurea | 110-21-4 | nd | 1.118 | nd | nd | nd | nd | nd | nd | nd | nd | nd | nd | nd | nd | nd | nd | nd | nd |
| V.346 | 1-phenyl-1,3,3-trimethylindan | 3910-35-8 | nd | 0.471 | nd | nd | nd | nd | nd | nd | nd | nd | nd | nd | nd | nd | nd | nd | nd | nd |
| V.347 | 4,5-dimethyl-1,3-thiazol-2-amine | 2289-75-0 | nd | nd | 6.827 | nd | nd | nd | nd | nd | nd | nd | nd | nd | nd | nd | nd | nd | nd | nd |
| V.348 | 2-methyltetrahydrothiophene | 1795-09-1 | nd | nd | 9.727 | nd | nd | nd | nd | nd | nd | nd | nd | nd | nd | nd | nd | nd | nd | nd |
| V.349 | 2-pentadecyl-4-(hexadecyloxy)-1,3-dioxane | 56599-40-7 | nd | nd | 14.937 | nd | nd | nd | nd | nd | nd | nd | nd | nd | 8.702 | 7.763 | 11.312 | nd | 6.456 | 12.206 |
| V.350 | Oxamic hydrazide | 515-96-8 | nd | nd | nd | 3.010 | nd | nd | nd | nd | nd | nd | nd | nd | nd | nd | nd | nd | nd | nd |
| V.351 | Haloxazolam | 59128-97-1 | nd | nd | nd | nd | 2.321 | nd | nd | nd | nd | nd | nd | nd | nd | nd | nd | nd | nd | nd |
| V.352 | D-glucal | 13265-84-4 | nd | nd | nd | nd | 1.077 | nd | nd | nd | nd | nd | nd | nd | nd | nd | nd | nd | nd | nd |
| V.353 | Naphthalene | 91-20-3 | nd | nd | nd | nd | nd | 1.347 | nd | nd | nd | nd | nd | nd | nd | nd | nd | nd | nd | nd |
| V.354 | 4,4'-diheptylazoxybenzene | 37592-89-5 | nd | nd | nd | nd | nd | 0.667 | nd | nd | nd | nd | nd | nd | nd | nd | nd | nd | nd | nd |
| V.355 | 5-hydroxyuridine | 957-77-7 | nd | nd | nd | nd | nd | nd | 0.744 | nd | nd | nd | nd | nd | nd | nd | nd | nd | nd | nd |
| V.356 | Carbohydrazide | 497-18-7 | nd | nd | nd | nd | nd | nd | nd | 2.083 | nd | nd | nd | nd | nd | nd | nd | nd | nd | nd |
| V.357 | 2-hexylthiophene | 18794-77-9 | nd | nd | nd | nd | nd | nd | nd | 0.032 | nd | nd | nd | nd | nd | nd | nd | nd | nd | nd |
| V.358 | 1,5-dimethylpyrazole | 694-31-5 | nd | nd | nd | nd | nd | nd | nd | nd | 0.757 | nd | nd | nd | nd | nd | nd | nd | nd | nd |
| V.359 | Terbutol | 191811-2 | nd | nd | nd | nd | nd | nd | nd | nd | 0.456 | nd | nd | nd | nd | nd | 1.630 | nd | 2.373 | nd |
| V.360 | 2-amino-4,4,6,6-tetramethyl-4,6-dihydrothieno[2,3-c]furan-3-carbonitrile(saltdata: free) | 447412-24-0 | nd | nd | nd | nd | nd | nd | nd | nd | nd | nd | nd | 1.740 | nd | nd | nd | nd | nd | nd |
| V.361 | Isobutyroin | 815-77-0 | nd | nd | nd | nd | nd | nd | nd | nd | nd | nd | nd | 1.827 | nd | nd | nd | nd | nd | nd |
| V.362 | 1,1-dimethyl-2-methylenehydrazine | 2035-89-4 | nd | nd | nd | nd | nd | nd | nd | nd | nd | nd | nd | 0.197 | nd | nd | nd | nd | nd | nd |
| V.363 | 2,6-di-tert-butyl-4-methylphenol | 128-37-0 | nd | nd | nd | nd | nd | nd | nd | nd | nd | nd | nd | nd | 1.673 | 2.435 | nd | nd | nd | nd |
| V.364 | (-)-Camphor | 464-48-2 | nd | nd | nd | nd | nd | nd | nd | nd | nd | nd | nd | nd | nd | 1.482 | 6.167 | 6.644 | nd | 1.889 |
| V.365 | 3,4-dimethylbenzohydrazide | 42596-61-2 | nd | nd | nd | nd | nd | nd | nd | nd | nd | nd | nd | nd | nd | 0.416 | nd | nd | nd | nd |
| V.366 | (1-propylheptadecyl)benzene. | 2400-3-5 | nd | nd | nd | nd | nd | nd | nd | nd | nd | nd | nd | nd | nd | 1.500 | nd | nd | nd | nd |
| V.367 | Di-n-decyl sulphone | 111530-37-1 | nd | nd | nd | nd | nd | nd | nd | nd | nd | nd | nd | nd | nd | nd | 1.340 | 2.813 | nd | nd |
| V.368 | 2-(3-methyl-3-nitrobutyl)-1,3-dioxolane | 57620-56-1 | nd | nd | nd | nd | nd | nd | nd | nd | nd | nd | nd | nd | nd | nd | 0.339 | nd | nd | nd |
| V.369 | 2-chloroethanesulfonyl chloride | 1622-32-8 | nd | nd | nd | nd | nd | nd | nd | nd | nd | nd | nd | nd | nd | nd | 0.243 | nd | nd | nd |
| V.370 | 4-methyl-2-pentadecyl-1,3-dioxolane | 54950-56-0 | nd | nd | nd | nd | nd | nd | nd | nd | nd | nd | nd | nd | nd | nd | nd | 0.083 | nd | nd |
| V.371 | 2-(benzylamino)nicotinonitrile | 50351-72-9 | nd | nd | nd | nd | nd | nd | nd | nd | nd | nd | nd | nd | nd | nd | nd | nd | nd | 0.566 |
| V.372 | 5-amino-1,3,4-thiadiazole-2-thiol | 2349-67-9 | nd | nd | nd | nd | nd | nd | nd | nd | nd | nd | nd | nd | nd | nd | nd | nd | nd | 1.516 |
| V.373 | 2-heptyl-1,3-dioxalane octanal glycol acetal | 4359-57-3 | nd | nd | nd | nd | nd | nd | nd | nd | nd | nd | nd | nd | nd | nd | nd | nd | nd | 0.354 |
| **Free amino acid (μg/100mL)** | | | | | | | | | | | | | | | | | | | | |
| F.1 | Aspartic acid | 6899-3-2 | 2.760 | 2.710 | 2.810 | 1.610 | 1.550 | 1.670 | 3.440 | 3.350 | 3.530 | 1.700 | 1.650 | 1.750 | 5.430 | 5.320 | 5.540 | 2.500 | 2.410 | 2.590 |
| F.2 | Threonine | 72-19-5 | 27.040 | 26.950 | 27.130 | 22.540 | 22.380 | 22.700 | 62.480 | 61.010 | 63.950 | 20.530 | 20.320 | 20.740 | 88.880 | 88.710 | 89.050 | 28.780 | 29.210 | 28.350 |
| F.3 | Serine | 302-84-1 | 3.620 | 3.690 | 3.550 | 3.670 | 3.590 | 3.750 | 5.210 | 5.120 | 5.300 | 3.070 | 3.160 | 2.980 | 7.200 | 7.290 | 7.110 | 3.550 | 3.490 | 3.610 |
| F.4 | Glutamic acid | 56-86-0 | 5.280 | 5.210 | 5.350 | 5.580 | 5.510 | 5.650 | 5.600 | 5.690 | 5.510 | 4.260 | 4.190 | 4.330 | 7.240 | 7.160 | 7.320 | 5.920 | 5.890 | 5.950 |
| F.5 | Glycine | 56-40-6 | 2.420 | 2.490 | 2.350 | 1.560 | 1.510 | 1.610 | 1.670 | 1.610 | 1.730 | 1.330 | 1.390 | 1.270 | 2.950 | 2.840 | 3.060 | 1.810 | 1.890 | 1.730 |
| F.6 | Alanine | 338-69-2 | 7.360 | 7.310 | 7.410 | 5.650 | 5.690 | 5.610 | 8.270 | 8.160 | 8.380 | 4.840 | 4.810 | 4.870 | 11.750 | 11.680 | 11.820 | 6.690 | 6.760 | 6.620 |
| F.7 | Cystine | 52-90-4 | 0.930 | 0.890 | 0.970 | 0.850 | 0.810 | 0.890 | 0.900 | 0.850 | 0.950 | 0.840 | 0.890 | 0.790 | 0.910 | 0.950 | 0.870 | 0.860 | 0.890 | 0.830 |
| F.8 | Valine | 7004-3-7 | 5.630 | 5.560 | 5.700 | 4.720 | 4.790 | 4.650 | 6.570 | 6.490 | 6.650 | 3.730 | 3.780 | 3.680 | 10.530 | 10.460 | 10.600 | 5.540 | 5.460 | 5.620 |
| F.9 | Methionine | 63-68-3 | 0.770 | 0.710 | 0.830 | 0.640 | 0.690 | 0.590 | 1.230 | 1.210 | 1.250 | 0.750 | 0.760 | 0.740 | 1.180 | 1.110 | 1.250 | 0.690 | 0.610 | 0.770 |
| F.10 | Isoleucine | 73-32-5 | 3.040 | 2.990 | 3.090 | 2.160 | 2.060 | 2.260 | 2.910 | 2.990 | 2.830 | 1.800 | 1.880 | 1.720 | 5.710 | 5.790 | 5.630 | 2.540 | 2.590 | 2.490 |
| F.11 | Leucine | 3588-60-1 | 3.710 | 3.790 | 3.630 | 2.570 | 2.660 | 2.480 | 2.910 | 2.860 | 2.960 | 2.280 | 2.210 | 2.350 | 4.690 | 4.750 | 4.630 | 2.800 | 2.740 | 2.860 |
| F.12 | Tyrosine | 70642-86-3 | 1.980 | 1.910 | 2.050 | 0.290 | 0.210 | 0.370 | 1.660 | 1.580 | 1.740 | 0.690 | 0.610 | 0.770 | 2.640 | 2.760 | 2.520 | 1.280 | 1.210 | 1.350 |
| F.13 | Phenylalanine | 673-31-4 | 2.340 | 2.390 | 2.290 | 1.510 | 1.480 | 1.540 | 2.180 | 2.080 | 2.280 | 1.350 | 1.390 | 1.310 | 3.550 | 3.630 | 3.470 | 1.890 | 1.800 | 1.980 |
| F.14 | Lysine | 56-87-1 | 4.080 | 4.160 | 4.000 | 2.850 | 2.810 | 2.890 | 4.010 | 4.120 | 3.900 | 2.250 | 2.190 | 2.310 | 5.400 | 5.490 | 5.310 | 3.220 | 3.120 | 3.320 |
| F.15 | Ammonia | 7664-41-7 | 3.030 | 3.130 | 2.930 | 5.820 | 5.750 | 5.890 | 3.610 | 3.690 | 3.530 | 5.400 | 5.490 | 5.310 | 4.010 | 4.190 | 3.830 | 4.790 | 4.850 | 4.730 |
| F.16 | Histidine | 71-00-1 | 1.900 | 1.810 | 1.990 | 1.760 | 1.720 | 1.800 | 2.520 | 2.590 | 2.450 | 1.370 | 1.310 | 1.430 | 3.940 | 3.890 | 3.990 | 2.140 | 2.190 | 2.090 |
| F.17 | Arginine | 7004-12-8 | 3.000 | 3.120 | 2.880 | 1.280 | 1.210 | 1.350 | 3.370 | 3.290 | 3.450 | 1.870 | 1.810 | 1.930 | 3.830 | 3.760 | 3.900 | 2.440 | 2.350 | 2.530 |
| F.18 | Proline | 344-25-2 | 1.520 | 1.610 | 1.430 | 2.190 | 2.090 | 2.290 | 2.210 | 2.280 | 2.140 | 1.200 | 1.120 | 1.280 | 4.150 | 4.050 | 4.250 | 2.190 | 2.090 | 2.290 |
| **Organic acid (g/L)** | | | | | | | | | | | | | | | | | | | | |
| A.1 | Lactic acid | 50-21-5 | 2.104 | 2.771 | 2.630 | 3.900 | 5.372 | 4.577 | 4.183 | 3.439 | 3.419 | 9.806 | 11.419 | 11.446 | 2.514 | 2.954 | 2.805 | 19.904 | 16.674 | 17.555 |
| A.2 | Acetic acid | 64-19-7 | 0.842 | 0.893 | 0.875 | 0.972 | 0.949 | 0.988 | 0.933 | 0.907 | 0.922 | 1.330 | 1.393 | 1.371 | 0.875 | 0.860 | 0.917 | 1.358 | 1.390 | 1.443 |
| A.3 | Α-ketoglutaric acid | 328-50-7 | 0.016 | 0.013 | 0.011 | 0.009 | 0.010 | 0.009 | 0.019 | 0.019 | 0.017 | 0.027 | 0.038 | 0.019 | 0.015 | 0.007 | 0.018 | 0.050 | 0.078 | 0.071 |
| A.4 | Butyric acid | 107-92-6 | nd | nd | nd | 0.237 | 0.201 | 0.268 | nd | nd | nd | 0.767 | 0.843 | 0.849 | nd | nd | nd | 2.168 | 2.058 | 1.997 |
| A.5 | Oxalic acid | 144-62-7 | 4.060 | 5.007 | 3.461 | 3.839 | 4.830 | 4.733 | 5.117 | 4.773 | 4.532 | 7.598 | 8.002 | 6.880 | 4.265 | 4.278 | 4.925 | 9.708 | 10.210 | 10.466 |

nd: not detected.

Table S5 CLR abundance of OTU during the fermentation in different containers.

| Phylum | Class | Order | Family | Genus | Species | OTU | CLR  GL-6 d | CLR  GL-12 d | CLR  PL-6 d | CLR  PL-12 d | CLR  PO-6 d | CLR  PO-12 d |
| --- | --- | --- | --- | --- | --- | --- | --- | --- | --- | --- | --- | --- |
| p__Firmicutes | c__Clostridia | o__Clostridiales | f__Clostridiaceae_1 | g__Clostridium_sensu_stricto_4 | s__Clostridium_fallax | OTU174 | -0.17509 | -0.60521 | -0.72192 | -0.67315 | -0.6489 | -0.63307 |
| p__Firmicutes | c__Bacilli | o__Lactobacillales | f__Lactobacillaceae | g__Pediococcus | s__Pediococcus_ethanolidurans | OTU204 | 1.861594 | 2.497878 | 1.54 | 1.889799 | 1.412771 | 1.593593 |
| p__Proteobacteria | c__Gammaproteobacteria | o__Enterobacteriales | f__Enterobacteriaceae | g__unclassified_f__Enterobacteriaceae | s__unclassified_f__Enterobacteriaceae | OTU394 | -0.61458 | -0.60521 | -0.5222 | -0.3696 | -0.22156 | 3.07673 |
| p__Firmicutes | c__Bacilli | o__Lactobacillales | f__Lactobacillaceae | g__Lactobacillus | s__unclassified_g__Lactobacillus | OTU386 | -0.61458 | -0.60521 | -0.36728 | -0.26126 | -0.33397 | 1.148742 |
| p__Proteobacteria | c__Gammaproteobacteria | o__Enterobacteriales | f__Enterobacteriaceae | g__unclassified_f__Enterobacteriaceae | s__unclassified_f__Enterobacteriaceae | OTU34 | 0.405881 | -0.60521 | -0.36728 | -0.5022 | -0.22156 | -0.10383 |
| p__Firmicutes | c__Clostridia | o__Clostridiales | f__Lachnospiraceae | g__Lachnospiraceae_NK4A136_group | s__unclassified_g__Lachnospiraceae_NK4A136_group | OTU130 | -0.61458 | -0.60521 | -0.5222 | -0.3696 | -0.22156 | -0.63307 |
| p__Bacteroidetes | c__Bacteroidia | o__Bacteroidales | f__Marinifilaceae | g__Odoribacter | s__uncultured_bacterium_g__Odoribacter | OTU372 | -0.61458 | -0.60521 | -0.72192 | -0.67315 | -0.6489 | -0.20011 |
| p__Firmicutes | c__Bacilli | o__Lactobacillales | f__Lactobacillaceae | g__Lactobacillus | s__unclassified_g__Lactobacillus | OTU388 | -0.61458 | 3.02725 | 0.240509 | 3.398685 | 0.790907 | 4.635938 |
| p__Verrucomicrobia | c__Verrucomicrobiae | o__Verrucomicrobiales | f__Akkermansiaceae | g__Akkermansia | s__Akkermansia_muciniphila | OTU385 | -0.07735 | -0.60521 | -0.5222 | -0.3696 | -0.6489 | 0.864706 |
| p__Firmicutes | c__Bacilli | o__Bacillales | f__Bacillaceae | g__Bacillus | s__unclassified_g__Bacillus | OTU190 | 0.148796 | 0.25957 | -0.5222 | 0.098925 | -0.6489 | -0.45337 |
| p__Proteobacteria | c__Alphaproteobacteria | o__Sphingomonadales | f__Sphingomonadaceae | g__Sphingomonas | s__unclassified_g__Sphingomonas | OTU58 | -0.29069 | -0.60521 | -0.04098 | -0.67315 | -0.47154 | -0.20011 |
| p__Proteobacteria | c__Gammaproteobacteria | o__Enterobacteriales | f__Enterobacteriaceae | g__unclassified_f__Enterobacteriaceae | s__unclassified_f__Enterobacteriaceae | OTU194 | 1.482922 | 2.7862 | 0.482319 | -0.26126 | 0.948054 | 1.425577 |
| p__Proteobacteria | c__Gammaproteobacteria | o__Enterobacteriales | f__Enterobacteriaceae | g__unclassified_f__Enterobacteriaceae | s__unclassified_f__Enterobacteriaceae | OTU191 | -0.17509 | 2.277841 | 0.113933 | -0.16966 | -0.6489 | -0.63307 |
| p__Firmicutes | c__Clostridia | o__Clostridiales | f__Ruminococcaceae | g__Oscillibacter | s__Clostridium]_leptum_g__Oscillibacter | OTU391 | -0.43218 | -0.60521 | -0.36728 | -0.67315 | -0.6489 | -0.45337 |
| p__Firmicutes | c__Bacilli | o__Lactobacillales | f__Lactobacillaceae | g__Lactobacillus | s__unclassified_g__Lactobacillus | OTU165 | 3.008906 | -0.60521 | -0.72192 | -0.67315 | -0.6489 | -0.63307 |
| p__Firmicutes | c__Clostridia | o__Clostridiales | f__Ruminococcaceae | g__Ruminiclostridium | s__uncultured_bacterium_g__Ruminiclostridium | OTU81 | -0.17509 | -0.60521 | -0.13369 | -0.16966 | -0.33397 | -0.63307 |
| p__Firmicutes | c__Bacilli | o__Lactobacillales | f__Lactobacillaceae | g__Lactobacillus | s__unclassified_g__Lactobacillus | OTU44 | 3.264596 | 2.890967 | 3.055453 | 2.864468 | 0.152133 | 2.658411 |
| p__Firmicutes | c__Bacilli | o__Lactobacillales | f__Lactobacillaceae | g__Lactobacillus | s__unclassified_g__Lactobacillus | OTU42 | 1.490608 | 0.665411 | 0.595147 | 0.695122 | -0.6489 | 0.282837 |
| p__Proteobacteria | c__Alphaproteobacteria | o__Sphingomonadales | f__Sphingomonadaceae | g__Sphingomonas | s__unclassified_g__Sphingomonas | OTU274 | -0.43218 | -0.28655 | -0.24071 | 0.610086 | -0.6489 | -0.63307 |
| p__Proteobacteria | c__Gammaproteobacteria | o__Enterobacteriales | f__Enterobacteriaceae | g__Escherichia-Shigella | s__unclassified_g__Escherichia-Shigella | OTU357 | 0.886292 | -0.60521 | -0.13369 | 0.483173 | 0.633118 | 1.92086 |
| p__Proteobacteria | c__Gammaproteobacteria | o__Betaproteobacteriales | f__Burkholderiaceae | g__Burkholderia-Caballeronia-Paraburkholderia | s__unclassified_g__Burkholderia-Caballeronia-Paraburkholderia | OTU45 | -0.43218 | -0.60521 | -0.5222 | -0.67315 | -0.6489 | -0.63307 |
| p__Firmicutes | c__Bacilli | o__Lactobacillales | f__Leuconostocaceae | g__Weissella | s__unclassified_g__Weissella | OTU197 | 0.446802 | 1.084091 | -0.13369 | 0.098925 | 0.34335 | 0.691292 |
| p__Gemmatimonadetes | c__Gemmatimonadetes | o__Gemmatimonadales | f__Gemmatimonadaceae | g__Gemmatimonas | s__unclassified_g__Gemmatimonas | OTU19 | -0.61458 | -0.60521 | -0.72192 | -0.67315 | -0.6489 | -0.63307 |
| p__Firmicutes | c__Bacilli | o__Lactobacillales | f__Lactobacillaceae | g__Lactobacillus | s__unclassified_g__Lactobacillus | OTU23 | -0.07735 | -0.28655 | 0.482319 | 0.242232 | 0.152133 | 0.329126 |
| p__Proteobacteria | c__Gammaproteobacteria | o__Xanthomonadales | f__Rhodanobacteraceae | g__Chujaibacter | s__uncultured_gamma_proteobacterium_g__Chujaibacter | OTU341 | 0.007312 | -0.42575 | -0.24071 | -0.5022 | -0.12653 | -0.63307 |
| p__Bacteroidetes | c__Bacteroidia | o__Bacteroidales | f__Marinifilaceae | g__Odoribacter | s__uncultured_bacterium_g__Odoribacter | OTU77 | -0.61458 | -0.60521 | -0.5222 | -0.67315 | -0.6489 | -0.45337 |
| p__Firmicutes | c__Erysipelotrichia | o__Erysipelotrichales | f__Erysipelotrichaceae | g__unclassified_f__Erysipelotrichaceae | s__unclassified_f__Erysipelotrichaceae | OTU275 | -0.61458 | -0.60521 | -0.72192 | -0.5022 | -0.6489 | -0.63307 |
| p__Bacteroidetes | c__Bacteroidia | o__Bacteroidales | f__Muribaculaceae | g__norank_f__Muribaculaceae | s__uncultured_bacterium_g__norank_f__Muribaculaceae | OTU92 | -0.61458 | -0.60521 | -0.72192 | -0.5022 | -0.6489 | -0.45337 |
| p__Proteobacteria | c__Alphaproteobacteria | o__Sphingomonadales | f__Sphingomonadaceae | g__Sphingomonas | s__unclassified_g__Sphingomonas | OTU187 | -0.29069 | 0.54623 | -0.5222 | -0.67315 | -0.6489 | -0.63307 |
| p__Bacteroidetes | c__Bacteroidia | o__Bacteroidales | f__Muribaculaceae | g__norank_f__Muribaculaceae | s__unclassified_g__norank_f__Muribaculaceae | OTU304 | -0.61458 | -0.60521 | -0.24071 | -0.67315 | -0.6489 | -0.63307 |
| p__Firmicutes | c__Bacilli | o__Lactobacillales | f__Streptococcaceae | g__Streptococcus | s__Streptococcus_danieliae | OTU37 | -0.61458 | -0.42575 | -0.72192 | -0.67315 | -0.33397 | -0.31399 |
| p__Firmicutes | c__Bacilli | o__Lactobacillales | f__Leuconostocaceae | g__Leuconostoc | s__unclassified_g__Leuconostoc | OTU303 | -0.61458 | -0.07666 | 0.180101 | 0.866072 | -0.33397 | -0.45337 |
| p__Bacteroidetes | c__Bacteroidia | o__Bacteroidales | f__Muribaculaceae | g__norank_f__Muribaculaceae | s__uncultured_bacterium_g__norank_f__Muribaculaceae | OTU61 | -0.61458 | -0.60521 | -0.5222 | -0.67315 | -0.6489 | -0.63307 |
| p__Firmicutes | c__Clostridia | o__Clostridiales | f__Lachnospiraceae | g__Roseburia | s__unclassified_g__Roseburia | OTU98 | -0.61458 | -0.60521 | -0.5222 | -0.67315 | -0.6489 | -0.63307 |
| p__Bacteroidetes | c__Bacteroidia | o__Bacteroidales | f__Muribaculaceae | g__norank_f__Muribaculaceae | s__uncultured_bacterium_g__norank_f__Muribaculaceae | OTU66 | -0.61458 | -0.60521 | -0.72192 | -0.67315 | -0.6489 | -0.63307 |
| p__Firmicutes | c__Erysipelotrichia | o__Erysipelotrichales | f__Erysipelotrichaceae | g__Erysipelatoclostridium | s__unclassified_g__Erysipelatoclostridium | OTU336 | -0.17509 | -0.60521 | -0.36728 | -0.67315 | -0.12653 | -0.45337 |
| p__Proteobacteria | c__Gammaproteobacteria | o__Betaproteobacteriales | f__Burkholderiaceae | g__Burkholderia-Caballeronia-Paraburkholderia | s__unclassified_g__Burkholderia-Caballeronia-Paraburkholderia | OTU5 | 0.007312 | 0.35573 | -0.13369 | -0.26126 | 0.38314 | 0.178492 |
| p__Firmicutes | c__Erysipelotrichia | o__Erysipelotrichales | f__Erysipelotrichaceae | g__norank_f__Erysipelotrichaceae | s__uncultured_Allobaculum_sp._g__norank | OTU4 | -0.61458 | -0.17282 | -0.5222 | -0.5022 | -0.33397 | -0.31399 |
| p__Firmicutes | c__Bacilli | o__Lactobacillales | f__Lactobacillaceae | g__Lactobacillus | s__unclassified_g__Lactobacillus | OTU345 | -0.61458 | 3.231139 | 0.559537 | 3.055526 | 4.228607 | -0.45337 |
| p__Firmicutes | c__Bacilli | o__Lactobacillales | f__Lactobacillaceae | g__Lactobacillus | s__unclassified_g__Lactobacillus | OTU223 | 2.83159 | 1.465973 | 5.064197 | 3.259664 | 2.995236 | 2.316055 |
| p__Proteobacteria | c__Gammaproteobacteria | o__Enterobacteriales | f__Enterobacteriaceae | g__unclassified_f__Enterobacteriaceae | s__unclassified_f__Enterobacteriaceae | OTU70 | -0.61458 | -0.60521 | -0.13369 | -0.67315 | -0.47154 | -0.63307 |
| p__Firmicutes | c__Bacilli | o__Lactobacillales | f__Lactobacillaceae | g__Lactobacillus | s__unclassified_g__Lactobacillus | OTU111 | -0.61458 | -0.60521 | -0.72192 | -0.67315 | -0.6489 | -0.63307 |
| p__Firmicutes | c__Erysipelotrichia | o__Erysipelotrichales | f__Erysipelotrichaceae | g__Turicibacter | s__Turicibacter_sp._LA61 | OTU221 | -0.43218 | 0.205291 | -0.04098 | -0.26126 | -0.47154 | -0.45337 |
| p__Firmicutes | c__Bacilli | o__Lactobacillales | f__Lactobacillaceae | g__Lactobacillus | s__unclassified_g__Lactobacillus | OTU126 | -0.43218 | -0.17282 | -0.5222 | 0.510817 | -0.0442 | 0.232841 |
| p__Firmicutes | c__Bacilli | o__Lactobacillales | f__Lactobacillaceae | g__Lactobacillus | s__unclassified_g__Lactobacillus | OTU123 | -0.61458 | -0.60521 | -0.72192 | -0.67315 | -0.6489 | -0.63307 |
| p__Firmicutes | c__Bacilli | o__Lactobacillales | f__Lactobacillaceae | g__Lactobacillus | s__unclassified_g__Lactobacillus | OTU122 | -0.61458 | -0.60521 | -0.72192 | -0.67315 | -0.6489 | -0.63307 |
| p__Bacteroidetes | c__Bacteroidia | o__Bacteroidales | f__Muribaculaceae | g__norank_f__Muribaculaceae | s__unclassified_g__norank_f__Muribaculaceae | OTU91 | -0.43218 | -0.42575 | 0.040787 | -0.5022 | -0.22156 | -0.45337 |
| p__Verrucomicrobia | c__Verrucomicrobiae | o__Verrucomicrobiales | f__Akkermansiaceae | g__Akkermansia | s__Akkermansia_muciniphila | OTU180 | -0.29069 | 0.741892 | -0.72192 | -0.3696 | -0.6489 | -0.63307 |
| p__Firmicutes | c__Clostridia | o__Clostridiales | f__Lachnospiraceae | g__unclassified_f__Lachnospiraceae | s__unclassified_f__Lachnospiraceae | OTU311 | 0.209228 | -0.28655 | -0.13369 | -0.09031 | -0.12653 | 0.118959 |
| p__Firmicutes | c__Bacilli | o__Lactobacillales | f__Lactobacillaceae | g__Lactobacillus | s__unclassified_g__Lactobacillus | OTU263 | 0.521482 | 0.944893 | 1.309957 | 1.306957 | -0.0442 | 0.329126 |
| p__Proteobacteria | c__Gammaproteobacteria | o__Enterobacteriales | f__Enterobacteriaceae | g__unclassified_f__Enterobacteriaceae | s__unclassified_f__Enterobacteriaceae | OTU167 | 3.326634 | -0.17282 | 0.180101 | -0.67315 | 1.674722 | 0.37222 |
| p__Firmicutes | c__Clostridia | o__Clostridiales | f__Family_XI_o__Clostridiales | g__unclassified_f__Family_XI_o__Clostridiales | s__unclassified_f__Family_XI_o__Clostridiales | OTU277 | -0.61458 | -0.60521 | -0.72192 | -0.5022 | -0.6489 | -0.63307 |
| p__Bacteroidetes | c__Bacteroidia | o__Bacteroidales | f__Muribaculaceae | g__norank_f__Muribaculaceae | s__unclassified_g__norank_f__Muribaculaceae | OTU161 | -0.43218 | -0.60521 | -0.72192 | -0.67315 | -0.6489 | -0.63307 |
| p__Firmicutes | c__Clostridia | o__Clostridiales | f__Lachnospiraceae | g__unclassified_f__Lachnospiraceae | s__unclassified_f__Lachnospiraceae | OTU31 | 0.007312 | -0.17282 | -0.13369 | -0.5022 | -0.22156 | -0.02042 |
| p__Firmicutes | c__Bacilli | o__Lactobacillales | f__Leuconostocaceae | g__Leuconostoc | s__unclassified_g__Leuconostoc | OTU124 | -0.61458 | -0.60521 | -0.5222 | -0.67315 | -0.47154 | -0.63307 |
| p__Deferribacteres | c__Deferribacteres | o__Deferribacterales | f__Deferribacteraceae | g__Mucispirillum | s__Mucispirillum_schaedleri_ASF457 | OTU370 | -0.29069 | -0.42575 | -0.04098 | -0.67315 | -0.6489 | 0.450399 |
| p__Firmicutes | c__Bacilli | o__Lactobacillales | f__Leuconostocaceae | g__Leuconostoc | s__unclassified_g__Leuconostoc | OTU285 | -0.61458 | -0.07666 | 0.440231 | 0.695122 | -0.6489 | -0.63307 |
| p__Proteobacteria | c__Gammaproteobacteria | o__Betaproteobacteriales | f__Nitrosomonadaceae | g__Ellin6067 | s__unclassified_g__Ellin6067 | OTU195 | -0.61458 | -0.28655 | -0.72192 | -0.5022 | -0.6489 | -0.63307 |
| p__Proteobacteria | c__Gammaproteobacteria | o__Aeromonadales | f__Aeromonadaceae | g__Aeromonas | s__unclassified_g__Aeromonas | OTU53 | -0.61458 | -0.60521 | -0.24071 | -0.67315 | -0.6489 | -0.63307 |
| p__Firmicutes | c__Clostridia | o__Clostridiales | f__Ruminococcaceae | g__norank_f__Ruminococcaceae | s__unclassified_g__norank_f__Ruminococcaceae | OTU189 | -0.61458 | -0.42575 | -0.5222 | -0.67315 | -0.6489 | -0.63307 |
| p__Firmicutes | c__Clostridia | o__Clostridiales | f__Lachnospiraceae | g__unclassified_f__Lachnospiraceae | s__unclassified_f__Lachnospiraceae | OTU138 | -0.61458 | -0.60521 | -0.72192 | -0.67315 | -0.6489 | -0.63307 |
| p__Proteobacteria | c__Gammaproteobacteria | o__Enterobacteriales | f__Enterobacteriaceae | g__unclassified_f__Enterobacteriaceae | s__unclassified_f__Enterobacteriaceae | OTU244 | 1.027776 | 0.145836 | 3.466525 | -0.09031 | -0.47154 | -0.20011 |
| p__Firmicutes | c__Bacilli | o__Lactobacillales | f__Streptococcaceae | g__Lactococcus | s__unclassified_g__Lactococcus | OTU210 | 2.060796 | 2.039064 | 1.346186 | 1.215355 | 1.943384 | 1.369121 |
| p__Actinobacteria | c__Actinobacteria | o__Micrococcales | f__Micrococcaceae | g__Glutamicibacter | s__unclassified_g__Glutamicibacter | OTU360 | -0.43218 | -0.60521 | -0.5222 | -0.67315 | -0.47154 | 0.232841 |
| p__Proteobacteria | c__Gammaproteobacteria | o__Betaproteobacteriales | f__Burkholderiaceae | g__Paenalcaligenes | s__Paenalcaligenes_hominis | OTU247 | -0.61458 | -0.42575 | 0.296078 | -0.3696 | -0.6489 | -0.63307 |
| p__Firmicutes | c__Bacilli | o__Lactobacillales | f__Streptococcaceae | g__Lactococcus | s__unclassified_g__Lactococcus | OTU175 | 3.018586 | 0.608662 | 1.765923 | 0.15063 | 2.021091 | 0.739363 |
| p__Proteobacteria | c__Gammaproteobacteria | o__Enterobacteriales | f__Enterobacteriaceae | g__unclassified_f__Enterobacteriaceae | s__unclassified_f__Enterobacteriaceae | OTU211 | -0.29069 | 2.161237 | 3.212774 | 0.391572 | -0.0442 | 1.360623 |
| p__Firmicutes | c__Bacilli | o__Lactobacillales | f__Streptococcaceae | g__Lactococcus | s__unclassified_g__Lactococcus | OTU179 | 1.589437 | -0.42575 | -0.13369 | -0.67315 | -0.33397 | -0.45337 |
| p__Proteobacteria | c__Gammaproteobacteria | o__Betaproteobacteriales | f__Burkholderiaceae | g__Paenalcaligenes | s__unclassified_g__Paenalcaligenes | OTU112 | -0.61458 | -0.60521 | -0.72192 | -0.67315 | -0.6489 | -0.63307 |
| p__Firmicutes | c__Clostridia | o__Clostridiales | f__Clostridiaceae_1 | g__Clostridium_sensu_stricto_1 | s__unclassified_g__Clostridium_sensu_stricto_1 | OTU78 | -0.61458 | -0.60521 | -0.72192 | -0.5022 | -0.47154 | -0.45337 |
| p__Firmicutes | c__Clostridia | o__Clostridiales | f__Family_XI_o__Clostridiales | g__Anaerosalibacter | s__unclassified_g__Anaerosalibacter | OTU95 | -0.61458 | -0.60521 | -0.72192 | -0.67315 | -0.6489 | -0.63307 |
| p__Firmicutes | c__Clostridia | o__Clostridiales | f__Lachnospiraceae | g__Lachnospiraceae_NK4A136_group | s__Lachnospiraceae_bacterium_10-1 | OTU203 | 0.754638 | 1.010617 | 0.559537 | 0.58678 | 0.579474 | 0.665793 |
| p__Proteobacteria | c__Gammaproteobacteria | o__Enterobacteriales | f__Enterobacteriaceae | g__unclassified_f__Enterobacteriaceae | s__unclassified_f__Enterobacteriaceae | OTU300 | -0.29069 | 1.721405 | 1.772322 | 2.564338 | 0.093372 | 1.148742 |
| p__Firmicutes | c__Bacilli | o__Lactobacillales | f__Lactobacillaceae | g__Lactobacillus | s__unclassified_g__Lactobacillus | OTU129 | -0.61458 | -0.60521 | -0.72192 | -0.67315 | -0.6489 | -0.45337 |
| p__Firmicutes | c__Bacilli | o__Lactobacillales | f__Streptococcaceae | g__Lactococcus | s__unclassified_g__Lactococcus | OTU149 | 3.126825 | 2.065488 | 1.521966 | 2.274553 | 1.640597 | 1.660581 |
| p__Proteobacteria | c__Gammaproteobacteria | o__Betaproteobacteriales | f__Burkholderiaceae | g__Burkholderia-Caballeronia-Paraburkholderia | s__unclassified_g__Burkholderia-Caballeronia-Paraburkholderia | OTU262 | -0.29069 | -0.07666 | 0.522001 | 1.086868 | -0.22156 | -0.10383 |
| p__Proteobacteria | c__Alphaproteobacteria | o__Sphingomonadales | f__Sphingomonadaceae | g__Sphingomonas | s__unclassified_g__Sphingomonas | OTU296 | 0.007312 | -0.42575 | -0.36728 | 0.632513 | 0.205777 | -0.02042 |
| p__Firmicutes | c__Clostridia | o__Clostridiales | f__Ruminococcaceae | g__Ruminiclostridium | s__unclassified_g__Ruminiclostridium | OTU119 | -0.61458 | -0.60521 | -0.72192 | -0.5022 | -0.6489 | -0.31399 |
| p__Firmicutes | c__Clostridia | o__Clostridiales | f__Ruminococcaceae | g__GCA-900066225 | s__unclassified_g__GCA-900066225 | OTU99 | -0.61458 | -0.42575 | -0.72192 | -0.67315 | -0.6489 | -0.45337 |
| p__Firmicutes | c__Bacilli | o__Lactobacillales | f__Streptococcaceae | g__Lactococcus | s__unclassified_g__Lactococcus | OTU63 | -0.61458 | -0.60521 | -0.72192 | -0.67315 | -0.6489 | -0.31399 |
| p__Firmicutes | c__Bacilli | o__Lactobacillales | f__Lactobacillaceae | g__Lactobacillus | s__unclassified_g__Lactobacillus | OTU306 | -0.61458 | 3.121827 | 0.440231 | 4.146757 | 2.512577 | 2.9601 |
| p__Proteobacteria | c__Gammaproteobacteria | o__Betaproteobacteriales | f__unclassified_o__Betaproteobacteriales | g__unclassified_o__Betaproteobacteriales | s__unclassified_o__Betaproteobacteriales | OTU354 | -0.61458 | -0.60521 | -0.72192 | -0.67315 | -0.6489 | -0.63307 |
| p__Firmicutes | c__Bacilli | o__Lactobacillales | f__Leuconostocaceae | g__Leuconostoc | s__unclassified_g__Leuconostoc | OTU334 | -0.07735 | -0.60521 | -0.72192 | -0.67315 | 0.489089 | -0.63307 |
| p__Proteobacteria | c__Gammaproteobacteria | o__Enterobacteriales | f__Enterobacteriaceae | g__Escherichia-Shigella | s__unclassified_g__Escherichia-Shigella | OTU338 | -0.61458 | -0.60521 | -0.72192 | -0.67315 | -0.47154 | -0.63307 |
| p__Proteobacteria | c__Gammaproteobacteria | o__Pseudomonadales | f__Moraxellaceae | g__Acinetobacter | s__unclassified_g__Acinetobacter | OTU82 | -0.43218 | -0.60521 | -0.72192 | -0.67315 | -0.6489 | -0.63307 |
| p__Bacteroidetes | c__Bacteroidia | o__Bacteroidales | f__Muribaculaceae | g__norank_f__Muribaculaceae | s__uncultured_organism_g__norank_f__Muribaculaceae | OTU7 | -0.61458 | -0.60521 | -0.72192 | -0.67315 | -0.6489 | -0.63307 |
| p__Bacteroidetes | c__Bacteroidia | o__Bacteroidales | f__Rikenellaceae | g__Alistipes | s__Alistipes_finegoldii | OTU186 | -0.43218 | 0.080111 | -0.72192 | -0.67315 | -0.47154 | -0.63307 |
| p__Proteobacteria | c__Gammaproteobacteria | o__Enterobacteriales | f__Enterobacteriaceae | g__Escherichia-Shigella | s__unclassified_g__Escherichia-Shigella | OTU349 | 1.45929 | -0.07666 | 1.705516 | 0.391572 | 1.771112 | 0.053149 |
| p__Firmicutes | c__Bacilli | o__Lactobacillales | f__Lactobacillaceae | g__Lactobacillus | s__unclassified_g__Lactobacillus | OTU270 | -0.61458 | -0.60521 | -0.72192 | -0.5022 | -0.6489 | -0.63307 |
| p__Proteobacteria | c__Gammaproteobacteria | o__Betaproteobacteriales | f__Burkholderiaceae | g__Variovorax | s__unclassified_g__Variovorax | OTU350 | -0.43218 | -0.60521 | -0.72192 | -0.67315 | -0.47154 | -0.63307 |
| p__Proteobacteria | c__Gammaproteobacteria | o__Enterobacteriales | f__Enterobacteriaceae | g__unclassified_f__Enterobacteriaceae | s__unclassified_f__Enterobacteriaceae | OTU297 | 0.555764 | -0.60521 | 0.296078 | 2.432165 | 0.34335 | 0.232841 |
| p__Firmicutes | c__Bacilli | o__Lactobacillales | f__Lactobacillaceae | g__Lactobacillus | s__unclassified_g__Lactobacillus | OTU379 | -0.43218 | 0.309501 | 0.180101 | -0.16966 | -0.12653 | 3.922433 |
| p__Proteobacteria | c__Gammaproteobacteria | o__Enterobacteriales | f__Enterobacteriaceae | g__unclassified_f__Enterobacteriaceae | s__unclassified_f__Enterobacteriaceae | OTU240 | 0.362136 | 0.006637 | 1.639347 | 0.242232 | 0.093372 | 0.118959 |
| p__Proteobacteria | c__Gammaproteobacteria | o__Betaproteobacteriales | f__Burkholderiaceae | g__Burkholderia-Caballeronia-Paraburkholderia | s__unclassified_g__Burkholderia-Caballeronia-Paraburkholderia | OTU59 | -0.61458 | -0.42575 | 0.113933 | -0.3696 | -0.47154 | 0.329126 |
| p__Firmicutes | c__Bacilli | o__Lactobacillales | f__Lactobacillaceae | g__Lactobacillus | s__unclassified_g__Lactobacillus | OTU371 | 0.081992 | -0.60521 | 0.296078 | 0.562522 | -0.22156 | 1.854662 |
| p__Proteobacteria | c__Gammaproteobacteria | o__Enterobacteriales | f__Enterobacteriaceae | g__unclassified_f__Enterobacteriaceae | s__unclassified_f__Enterobacteriaceae | OTU110 | 0.485241 | 0.205291 | -0.5222 | 0.198194 | 1.218248 | 1.55092 |
| p__Proteobacteria | c__Gammaproteobacteria | o__Enterobacteriales | f__Enterobacteriaceae | g__unclassified_f__Enterobacteriaceae | s__unclassified_f__Enterobacteriaceae | OTU206 | 0.555764 | 1.839284 | -0.36728 | -0.09031 | 0.255125 | -0.10383 |
| p__Proteobacteria | c__Gammaproteobacteria | o__Enterobacteriales | f__Enterobacteriaceae | g__unclassified_f__Enterobacteriaceae | s__unclassified_f__Enterobacteriaceae | OTU207 | 0.886292 | 0.637682 | 0.240509 | 0.510817 | 0.705734 | -0.02042 |
| p__Firmicutes | c__Clostridia | o__Clostridiales | f__Ruminococcaceae | g__Oscillibacter | s__Clostridium]_leptum_g__Oscillibacter | OTU135 | -0.43218 | -0.28655 | -0.5222 | -0.16966 | -0.6489 | -0.45337 |
| p__Proteobacteria | c__Gammaproteobacteria | o__Betaproteobacteriales | f__Burkholderiaceae | g__Paenalcaligenes | s__Paenalcaligenes_hominis | OTU330 | -0.29069 | -0.28655 | -0.72192 | -0.26126 | 0.420516 | -0.45337 |
| p__Firmicutes | c__Bacilli | o__Lactobacillales | f__Lactobacillaceae | g__Lactobacillus | s__unclassified_g__Lactobacillus | OTU153 | -0.17509 | -0.60521 | -0.5222 | -0.67315 | -0.6489 | -0.45337 |
| p__Bacteroidetes | c__Bacteroidia | o__Bacteroidales | f__Muribaculaceae | g__norank_f__Muribaculaceae | s__uncultured_bacterium_g__norank_f__Muribaculaceae | OTU12 | -0.61458 | -0.60521 | -0.72192 | -0.67315 | -0.6489 | -0.63307 |
| p__Firmicutes | c__Bacilli | o__Lactobacillales | f__Lactobacillaceae | g__Lactobacillus | s__unclassified_g__Lactobacillus | OTU154 | 2.907907 | -0.60521 | -0.72192 | -0.67315 | -0.6489 | -0.63307 |
| p__Firmicutes | c__Bacilli | o__Lactobacillales | f__Leuconostocaceae | g__Leuconostoc | s__unclassified_g__Leuconostoc | OTU163 | 1.305651 | -0.28655 | 0.440231 | 0.042289 | 0.455756 | 0.282837 |
| p__Firmicutes | c__Bacilli | o__Lactobacillales | f__Lactobacillaceae | g__Pediococcus | s__unclassified_g__Pediococcus | OTU177 | 2.371494 | -0.17282 | 0.595147 | -0.5022 | 0.705734 | 0.282837 |
| p__Firmicutes | c__Bacilli | o__Lactobacillales | f__Lactobacillaceae | g__Lactobacillus | s__unclassified_g__Lactobacillus | OTU55 | 2.553899 | 1.385888 | -0.04098 | 2.365757 | 2.214468 | 3.151091 |
| p__Firmicutes | c__Bacilli | o__Lactobacillales | f__Leuconostocaceae | g__Weissella | s__unclassified_g__Weissella | OTU184 | 0.081992 | 1.026021 | 0.113933 | 0.098925 | -0.6489 | -0.63307 |
| p__Bacteroidetes | c__Bacteroidia | o__Bacteroidales | f__Bacteroidaceae | g__Bacteroides | s__Bacteroides_acidifaciens | OTU272 | -0.61458 | -0.42575 | -0.04098 | -0.09031 | -0.6489 | -0.63307 |
| p__Firmicutes | c__Clostridia | o__Clostridiales | f__Ruminococcaceae | g__Ruminococcaceae_UCG-013 | s__unclassified_g__Ruminococcaceae_UCG-013 | OTU317 | -0.61458 | -0.60521 | -0.72192 | -0.67315 | -0.6489 | -0.45337 |
| p__Firmicutes | c__Bacilli | o__Bacillales | f__Bacillaceae | g__Cerasibacillus | s__unclassified_g__Cerasibacillus | OTU280 | -0.43218 | 0.006637 | 0.296078 | -0.02032 | -0.6489 | -0.10383 |
| p__Proteobacteria | c__Gammaproteobacteria | o__Enterobacteriales | f__Enterobacteriaceae | g__unclassified_f__Enterobacteriaceae | s__unclassified_f__Enterobacteriaceae | OTU343 | 0.845371 | 0.25957 | -0.13369 | -0.67315 | 3.300064 | 0.053149 |
| p__Proteobacteria | c__Gammaproteobacteria | o__Enterobacteriales | f__Enterobacteriaceae | g__unclassified_f__Enterobacteriaceae | s__unclassified_f__Enterobacteriaceae | OTU340 | -0.61458 | -0.60521 | -0.72192 | -0.67315 | -0.47154 | -0.63307 |
| p__Firmicutes | c__Erysipelotrichia | o__Erysipelotrichales | f__Erysipelotrichaceae | g__Faecalibaculum | s__Faecalibaculum_rodentium | OTU305 | -0.61458 | 0.512502 | 0.853104 | 0.674976 | -0.33397 | -0.20011 |
| p__Proteobacteria | c__Gammaproteobacteria | o__Betaproteobacteriales | f__Burkholderiaceae | g__Paenalcaligenes | s__Paenalcaligenes_hominis | OTU255 | -0.43218 | -0.42575 | -0.72192 | 0.321581 | -0.6489 | -0.63307 |
| p__Proteobacteria | c__Gammaproteobacteria | o__Xanthomonadales | f__Rhodanobacteraceae | g__Rhodanobacter | s__unclassified_g__Rhodanobacter | OTU302 | -0.61458 | -0.42575 | -0.5222 | -0.67315 | -0.6489 | -0.63307 |
| p__Firmicutes | c__Bacilli | o__Lactobacillales | f__Lactobacillaceae | g__Lactobacillus | s__unclassified_g__Lactobacillus | OTU62 | 3.166624 | 3.301946 | 1.110234 | 2.414877 | 2.158664 | 3.231386 |
| p__Firmicutes | c__Bacilli | o__Lactobacillales | f__Lactobacillaceae | g__Lactobacillus | s__unclassified_g__Lactobacillus | OTU65 | -0.61458 | -0.60521 | -0.72192 | -0.67315 | -0.6489 | -0.31399 |
| p__Proteobacteria | c__Gammaproteobacteria | o__Betaproteobacteriales | f__Burkholderiaceae | g__Alcaligenes | s__unclassified_g__Alcaligenes | OTU250 | -0.61458 | -0.42575 | -0.36728 | -0.67315 | -0.6489 | -0.45337 |
| p__Firmicutes | c__Bacilli | o__Lactobacillales | f__Lactobacillaceae | g__Lactobacillus | s__unclassified_g__Lactobacillus | OTU68 | 0.007312 | -0.60521 | 0.395425 | -0.3696 | -0.33397 | 0.519873 |
| p__Firmicutes | c__Bacilli | o__Bacillales | f__Bacillaceae | g__norank_f__Bacillaceae | s__uncultured_bacterium_g__norank_f__Bacillaceae | OTU188 | -0.61458 | -0.28655 | -0.5222 | -0.5022 | -0.6489 | -0.63307 |
| p__Firmicutes | c__Clostridia | o__Clostridiales | f__Ruminococcaceae | g__Ruminococcaceae_UCG-013 | s__unclassified_g__Ruminococcaceae_UCG-013 | OTU286 | -0.61458 | -0.60521 | -0.5222 | -0.67315 | -0.6489 | -0.63307 |
| p__Proteobacteria | c__Alphaproteobacteria | o__Sphingomonadales | f__Sphingomonadaceae | g__Sphingomonas | s__unclassified_g__Sphingomonas | OTU315 | 0.405881 | -0.28655 | 0.395425 | -0.67315 | 0.489089 | -0.20011 |
| p__Firmicutes | c__Bacilli | o__Lactobacillales | f__Leuconostocaceae | g__Leuconostoc | s__unclassified_g__Leuconostoc | OTU376 | 0.148796 | 0.810011 | 0.828742 | -0.02032 | 0.550793 | 0.984865 |
| p__Proteobacteria | c__Deltaproteobacteria | o__Desulfovibrionales | f__Desulfovibrionaceae | g__Desulfovibrio | s__unclassified_g__Desulfovibrio | OTU265 | -0.61458 | 0.080111 | 0.347527 | 0.391572 | -0.47154 | -0.63307 |
| p__Proteobacteria | c__Gammaproteobacteria | o__Betaproteobacteriales | f__Burkholderiaceae | g__Burkholderia-Caballeronia-Paraburkholderia | s__unclassified_g__Burkholderia-Caballeronia-Paraburkholderia | OTU196 | -0.17509 | 0.810011 | -0.72192 | -0.3696 | -0.22156 | -0.10383 |
| p__Bacteroidetes | c__Bacteroidia | o__Bacteroidales | f__Muribaculaceae | g__norank_f__Muribaculaceae | s__unclassified_g__norank_f__Muribaculaceae | OTU69 | -0.61458 | -0.60521 | -0.72192 | -0.67315 | -0.6489 | -0.63307 |
| p__Proteobacteria | c__Gammaproteobacteria | o__Enterobacteriales | f__Enterobacteriaceae | g__unclassified_f__Enterobacteriaceae | s__unclassified_f__Enterobacteriaceae | OTU155 | 2.28383 | 0.476846 | -0.36728 | 0.733473 | 0.865729 | -0.02042 |
| p__Acidobacteria | c__Holophagae | o__Subgroup_7 | f__norank_o__Subgroup_7 | g__norank_o__Subgroup_7 | s__uncultured_Desulfovirga_sp._g__norank | OTU264 | -0.61458 | -0.60521 | -0.72192 | -0.5022 | -0.6489 | -0.63307 |
| p__Verrucomicrobia | c__Verrucomicrobiae | o__Verrucomicrobiales | f__Akkermansiaceae | g__Akkermansia | s__Akkermansia_muciniphila | OTU344 | -0.07735 | -0.28655 | 0.113933 | -0.67315 | 0.633118 | -0.31399 |
| p__Proteobacteria | c__Gammaproteobacteria | o__Enterobacteriales | f__Enterobacteriaceae | g__Morganella | s__unclassified_g__Morganella | OTU365 | -0.61458 | -0.60521 | -0.72192 | -0.67315 | -0.47154 | -0.10383 |
| p__Firmicutes | c__Clostridia | o__Clostridiales | f__unclassified_o__Clostridiales | g__unclassified_o__Clostridiales | s__unclassified_o__Clostridiales | OTU359 | -0.61458 | -0.60521 | -0.72192 | -0.5022 | -0.6489 | -0.02042 |
| p__Firmicutes | c__Bacilli | o__Lactobacillales | f__Lactobacillaceae | g__Lactobacillus | s__unclassified_g__Lactobacillus | OTU236 | -0.61458 | -0.60521 | -0.5222 | -0.5022 | -0.6489 | -0.63307 |
| p__Firmicutes | c__Bacilli | o__Lactobacillales | f__Lactobacillaceae | g__Lactobacillus | s__unclassified_g__Lactobacillus | OTU231 | 1.16926 | 1.890802 | 2.843587 | 1.973773 | 1.256793 | 1.971125 |
| p__Firmicutes | c__Bacilli | o__Lactobacillales | f__Lactobacillaceae | g__Lactobacillus | s__unclassified_g__Lactobacillus | OTU230 | -0.29069 | 1.847492 | 5.049995 | 2.56519 | -0.33397 | 2.58818 |
| p__Bacteroidetes | c__Bacteroidia | o__Bacteroidales | f__Muribaculaceae | g__norank_f__Muribaculaceae | s__uncultured_bacterium_g__norank_f__Muribaculaceae | OTU41 | -0.61458 | -0.60521 | -0.72192 | -0.67315 | -0.6489 | -0.63307 |
| p__Firmicutes | c__Bacilli | o__Lactobacillales | f__Lactobacillaceae | g__Lactobacillus | s__unclassified_g__Lactobacillus | OTU284 | -0.61458 | -0.60521 | -0.72192 | 0.042289 | -0.6489 | -0.63307 |
| p__Firmicutes | c__Bacilli | o__Lactobacillales | f__Enterococcaceae | g__Enterococcus | s__unclassified_g__Enterococcus | OTU79 | 0.315148 | -0.60521 | 0.522001 | 0.632513 | 0.829452 | 0.329126 |
| p__Firmicutes | c__Bacilli | o__Lactobacillales | f__Lactobacillaceae | g__Lactobacillus | s__unclassified_g__Lactobacillus | OTU282 | -0.61458 | 0.145836 | -0.72192 | 1.610507 | -0.6489 | -0.63307 |
| p__Firmicutes | c__Erysipelotrichia | o__Erysipelotrichales | f__Erysipelotrichaceae | g__Faecalibaculum | s__Faecalibaculum_rodentium | OTU226 | -0.29069 | -0.60521 | 0.661316 | -0.67315 | -0.6489 | -0.63307 |
| p__Firmicutes | c__Bacilli | o__Lactobacillales | f__Lactobacillaceae | g__Lactobacillus | s__unclassified_g__Lactobacillus | OTU281 | -0.61458 | 0.909237 | -0.36728 | 2.067345 | -0.6489 | -0.45337 |
| p__Firmicutes | c__Bacilli | o__Lactobacillales | f__Leuconostocaceae | g__Leuconostoc | s__unclassified_g__Leuconostoc | OTU72 | -0.61458 | -0.60521 | -0.13369 | -0.67315 | -0.47154 | -0.02042 |
| p__Proteobacteria | c__Gammaproteobacteria | o__Pseudomonadales | f__Moraxellaceae | g__Acinetobacter | s__unclassified_g__Acinetobacter | OTU209 | -0.61458 | -0.42575 | -0.72192 | -0.67315 | -0.6489 | -0.63307 |
| p__Proteobacteria | c__Gammaproteobacteria | o__Betaproteobacteriales | f__Burkholderiaceae | g__unclassified_f__Burkholderiaceae | s__unclassified_f__Burkholderiaceae | OTU18 | -0.61458 | -0.60521 | -0.72192 | -0.67315 | -0.6489 | -0.63307 |
| p__Firmicutes | c__Clostridia | o__Clostridiales | f__Ruminococcaceae | g__Ruminiclostridium_9 | s__Firmicutes_bacterium_ASF500 | OTU116 | -0.43218 | -0.60521 | -0.36728 | -0.02032 | -0.22156 | -0.63307 |
| p__Actinobacteria | c__Actinobacteria | o__Micrococcales | f__Micrococcaceae | g__Sinomonas | s__Sinomonas_atrocyanea | OTU271 | -0.61458 | -0.42575 | -0.72192 | -0.67315 | -0.6489 | -0.63307 |
| p__Bacteroidetes | c__Bacteroidia | o__Bacteroidales | f__Bacteroidaceae | g__Bacteroides | s__unclassified_g__Bacteroides | OTU142 | -0.17509 | -0.60521 | -0.36728 | -0.5022 | -0.33397 | -0.63307 |
| p__Bacteroidetes | c__Bacteroidia | o__Bacteroidales | f__Muribaculaceae | g__norank_f__Muribaculaceae | s__uncultured_bacterium_g__norank_f__Muribaculaceae | OTU145 | -0.61458 | -0.60521 | -0.72192 | -0.67315 | -0.6489 | -0.63307 |
| p__Firmicutes | c__Bacilli | o__Lactobacillales | f__Enterococcaceae | g__Enterococcus | s__unclassified_g__Enterococcus | OTU85 | 0.209228 | -0.60521 | -0.04098 | 0.357606 | 0.34335 | -0.20011 |
| p__Firmicutes | c__Clostridia | o__Clostridiales | f__Lachnospiraceae | g__unclassified_f__Lachnospiraceae | s__unclassified_f__Lachnospiraceae | OTU220 | -0.29069 | 0.439028 | -0.13369 | -0.16966 | -0.6489 | -0.10383 |
| p__Firmicutes | c__Bacilli | o__Lactobacillales | f__Lactobacillaceae | g__Lactobacillus | s__unclassified_g__Lactobacillus | OTU80 | -0.43218 | -0.60521 | -0.72192 | 0.610086 | -0.47154 | -0.63307 |
| p__Proteobacteria | c__Gammaproteobacteria | o__Aeromonadales | f__Succinivibrionaceae | g__Ruminobacter | s__Ruminobacter_amylophilus | OTU52 | -0.61458 | -0.60521 | -0.72192 | -0.67315 | -0.6489 | -0.63307 |
| p__Firmicutes | c__Bacilli | o__Lactobacillales | f__Lactobacillaceae | g__Lactobacillus | s__unclassified_g__Lactobacillus | OTU15 | -0.61458 | -0.60521 | -0.24071 | -0.67315 | -0.6489 | -0.20011 |
| p__Proteobacteria | c__Gammaproteobacteria | o__Betaproteobacteriales | f__Methylophilaceae | g__Candidatus_Methylopumilus | s__unclassified_g__Candidatus_Methylopumilus | OTU104 | -0.61458 | -0.42575 | -0.72192 | -0.67315 | -0.6489 | -0.63307 |
| p__Verrucomicrobia | c__Verrucomicrobiae | o__Verrucomicrobiales | f__Akkermansiaceae | g__Akkermansia | s__Akkermansia_muciniphila | OTU164 | 0.555764 | -0.28655 | 0.113933 | -0.3696 | -0.47154 | 0.053149 |
| p__Proteobacteria | c__Gammaproteobacteria | o__Enterobacteriales | f__Enterobacteriaceae | g__unclassified_f__Enterobacteriaceae | s__unclassified_f__Enterobacteriaceae | OTU384 | -0.29069 | -0.28655 | 0.180101 | -0.67315 | -0.33397 | 1.711391 |
| p__Firmicutes | c__Bacilli | o__Bacillales | f__Planococcaceae | g__Savagea | s__uncultured_bacterium_g__Savagea | OTU133 | -0.07735 | 0.145836 | 0.113933 | -0.26126 | 0.255125 | -0.45337 |
| p__Firmicutes | c__Bacilli | o__Lactobacillales | f__Lactobacillaceae | g__Lactobacillus | s__unclassified_g__Lactobacillus | OTU106 | 0.405881 | 0.476846 | 0.240509 | 0.751758 | 0.658286 | 0.118959 |
| p__Firmicutes | c__Clostridia | o__Clostridiales | f__Ruminococcaceae | g__norank_f__Ruminococcaceae | s__unclassified_g__norank_f__Ruminococcaceae | OTU199 | -0.43218 | -0.28655 | -0.72192 | -0.5022 | -0.47154 | -0.63307 |
| p__Firmicutes | c__Clostridia | o__Clostridiales | f__Lachnospiraceae | g__unclassified_f__Lachnospiraceae | s__unclassified_f__Lachnospiraceae | OTU125 | -0.61458 | -0.60521 | -0.72192 | -0.67315 | -0.6489 | -0.63307 |
| p__Proteobacteria | c__Gammaproteobacteria | o__Enterobacteriales | f__Enterobacteriaceae | g__unclassified_f__Enterobacteriaceae | s__unclassified_f__Enterobacteriaceae | OTU40 | 0.72977 | -0.42575 | 0.395425 | -0.16966 | 0.255125 | -0.20011 |
| p__Proteobacteria | c__Gammaproteobacteria | o__Enterobacteriales | f__Enterobacteriaceae | g__unclassified_f__Enterobacteriaceae | s__unclassified_f__Enterobacteriaceae | OTU47 | 0.886292 | -0.60521 | 0.240509 | 0.15063 | 0.728156 | 0.282837 |
| p__Proteobacteria | c__Gammaproteobacteria | o__Enterobacteriales | f__Enterobacteriaceae | g__Escherichia-Shigella | s__unclassified_g__Escherichia-Shigella | OTU49 | 0.148796 | -0.07666 | 0.040787 | -0.26126 | -0.47154 | -0.31399 |
| p__Firmicutes | c__Clostridia | o__Clostridiales | f__Lachnospiraceae | g__unclassified_f__Lachnospiraceae | s__unclassified_f__Lachnospiraceae | OTU166 | -0.43218 | -0.60521 | -0.5222 | -0.67315 | -0.6489 | -0.45337 |
| p__Firmicutes | c__Bacilli | o__Lactobacillales | f__Leuconostocaceae | g__Weissella | s__unclassified_g__Weissella | OTU90 | 0.362136 | -0.42575 | 1.076362 | 1.154578 | 0.847857 | -0.02042 |
| p__Actinobacteria | c__Actinobacteria | o__Bifidobacteriales | f__Bifidobacteriaceae | g__Bifidobacterium | s__unclassified_g__Bifidobacterium | OTU346 | -0.43218 | -0.42575 | -0.36728 | -0.67315 | -0.22156 | -0.63307 |
| p__Proteobacteria | c__Gammaproteobacteria | o__Betaproteobacteriales | f__Nitrosomonadaceae | g__Ellin6067 | s__unclassified_g__Ellin6067 | OTU239 | -0.43218 | -0.42575 | -0.5222 | -0.67315 | -0.6489 | -0.63307 |
| p__Firmicutes | c__Bacilli | o__Lactobacillales | f__Lactobacillaceae | g__Lactobacillus | s__unclassified_g__Lactobacillus | OTU27 | -0.61458 | -0.60521 | -0.5222 | -0.3696 | -0.47154 | -0.45337 |
| p__Proteobacteria | c__Gammaproteobacteria | o__Pseudomonadales | f__Pseudomonadaceae | g__Pseudomonas | s__unclassified_g__Pseudomonas | OTU120 | -0.61458 | -0.60521 | -0.72192 | -0.67315 | -0.6489 | -0.63307 |
| p__Firmicutes | c__Bacilli | o__Lactobacillales | f__Lactobacillaceae | g__Lactobacillus | s__unclassified_g__Lactobacillus | OTU21 | 1.305651 | 1.701117 | 2.495721 | 1.851448 | 0.810481 | 2.348557 |
| p__Deferribacteres | c__Deferribacteres | o__Deferribacterales | f__Deferribacteraceae | g__Mucispirillum | s__Mucispirillum_schaedleri_ASF457 | OTU215 | -0.61458 | -0.07666 | -0.13369 | -0.67315 | -0.6489 | -0.63307 |
| p__Deferribacteres | c__Deferribacteres | o__Deferribacterales | f__Deferribacteraceae | g__Mucispirillum | s__Mucispirillum_schaedleri_ASF457 | OTU352 | 0.315148 | -0.42575 | 0.347527 | -0.5022 | 0.152133 | -0.10383 |
| p__Proteobacteria | c__Gammaproteobacteria | o__Pseudomonadales | f__Pseudomonadaceae | g__Pseudomonas | s__unclassified_g__Pseudomonas | OTU159 | -0.43218 | -0.60521 | -0.72192 | -0.67315 | -0.6489 | -0.63307 |
| p__Proteobacteria | c__Alphaproteobacteria | o__Sphingomonadales | f__Sphingomonadaceae | g__Sphingomonas | s__unclassified_g__Sphingomonas | OTU353 | -0.29069 | -0.28655 | -0.13369 | -0.5022 | 0.550793 | -0.63307 |
| p__Proteobacteria | c__Gammaproteobacteria | o__Vibrionales | f__Vibrionaceae | g__Vibrio | s__unclassified_g__Vibrio | OTU331 | -0.43218 | -0.60521 | -0.72192 | -0.5022 | 1.802736 | -0.20011 |
| p__Proteobacteria | c__Deltaproteobacteria | o__Desulfovibrionales | f__Desulfovibrionaceae | g__norank_f__Desulfovibrionaceae | s__unclassified_g__norank_f__Desulfovibrionaceae | OTU268 | -0.61458 | -0.28655 | -0.24071 | -0.16966 | -0.6489 | -0.63307 |
| p__Firmicutes | c__Clostridia | o__Clostridiales | f__Ruminococcaceae | g__Ruminiclostridium_5 | s__Ruminiclostridium_sp._KB18 | OTU241 | -0.61458 | -0.42575 | -0.5222 | -0.67315 | -0.6489 | -0.63307 |
| p__Firmicutes | c__Clostridia | o__Clostridiales | f__Ruminococcaceae | g__unclassified_f__Ruminococcaceae | s__unclassified_f__Ruminococcaceae | OTU243 | -0.61458 | -0.60521 | -0.5222 | -0.67315 | -0.6489 | -0.63307 |
| p__Firmicutes | c__Clostridia | o__Clostridiales | f__Ruminococcaceae | g__unclassified_f__Ruminococcaceae | s__unclassified_f__Ruminococcaceae | OTU362 | -0.61458 | -0.60521 | -0.72192 | -0.67315 | -0.6489 | -0.63307 |
| p__Firmicutes | c__Erysipelotrichia | o__Erysipelotrichales | f__Erysipelotrichaceae | g__Faecalibaculum | s__Faecalibaculum_rodentium | OTU16 | -0.29069 | -0.42575 | -0.72192 | -0.67315 | -0.12653 | -0.31399 |
| p__Actinobacteria | c__Actinobacteria | o__Coriobacteriales | f__Eggerthellaceae | g__Enterorhabdus | s__unclassified_g__Enterorhabdus | OTU38 | -0.61458 | -0.28655 | -0.5222 | -0.3696 | -0.22156 | -0.20011 |
| p__Proteobacteria | c__Deltaproteobacteria | o__Desulfovibrionales | f__Desulfovibrionaceae | g__Desulfovibrio | s__unclassified_g__Desulfovibrio | OTU358 | -0.29069 | -0.60521 | -0.72192 | -0.5022 | -0.47154 | 0.053149 |
| p__Firmicutes | c__Bacilli | o__Lactobacillales | f__Aerococcaceae | g__Aerococcus | s__unclassified_g__Aerococcus | OTU26 | -0.61458 | -0.60521 | -0.72192 | -0.67315 | -0.6489 | -0.63307 |
| p__Verrucomicrobia | c__Verrucomicrobiae | o__Verrucomicrobiales | f__Akkermansiaceae | g__Akkermansia | s__Akkermansia_muciniphila | OTU261 | 0.007312 | -0.42575 | -0.24071 | 0.610086 | -0.47154 | -0.02042 |
| p__Firmicutes | c__Bacilli | o__Bacillales | f__Bacillaceae | g__Bacillus | s__unclassified_g__Bacillus | OTU314 | -0.43218 | -0.60521 | -0.04098 | -0.16966 | -0.0442 | -0.02042 |
| p__Firmicutes | c__Clostridia | o__Clostridiales | f__Family_XI_o__Clostridiales | g__unclassified_f__Family_XI_o__Clostridiales | s__unclassified_f__Family_XI_o__Clostridiales | OTU332 | -0.07735 | -0.17282 | -0.36728 | -0.67315 | -0.12653 | -0.20011 |
| p__Firmicutes | c__Clostridia | o__Clostridiales | f__Family_XI_o__Clostridiales | g__Tissierella | s__unclassified_g__Tissierella | OTU132 | -0.61458 | -0.60521 | -0.72192 | -0.67315 | -0.6489 | -0.63307 |
| p__Bacteroidetes | c__Bacteroidia | o__Bacteroidales | f__Muribaculaceae | g__norank_f__Muribaculaceae | s__unclassified_g__norank_f__Muribaculaceae | OTU89 | -0.61458 | -0.60521 | -0.5222 | -0.67315 | -0.6489 | -0.63307 |
| p__Verrucomicrobia | c__Verrucomicrobiae | o__Verrucomicrobiales | f__Akkermansiaceae | g__Akkermansia | s__Akkermansia_muciniphila | OTU75 | -0.61458 | -0.60521 | -0.13369 | -0.09031 | -0.12653 | -0.63307 |
| p__Firmicutes | c__Bacilli | o__Lactobacillales | f__Lactobacillaceae | g__Lactobacillus | s__unclassified_g__Lactobacillus | OTU246 | -0.61458 | -0.60521 | -0.13369 | -0.67315 | -0.6489 | -0.63307 |
| p__Firmicutes | c__Bacilli | o__Lactobacillales | f__Lactobacillaceae | g__Lactobacillus | s__unclassified_g__Lactobacillus | OTU245 | 0.588286 | 1.582207 | 4.77707 | 1.223552 | -0.47154 | 0.919055 |
| p__Proteobacteria | c__Gammaproteobacteria | o__Cardiobacteriales | f__Wohlfahrtiimonadaceae | g__Ignatzschineria | s__unclassified_g__Ignatzschineria | OTU238 | -0.61458 | -0.42575 | -0.36728 | -0.26126 | -0.6489 | -0.63307 |
| p__Firmicutes | c__Bacilli | o__Bacillales | f__Planococcaceae | g__unclassified_f__Planococcaceae | s__unclassified_f__Planococcaceae | OTU157 | 0.405881 | -0.28655 | 0.180101 | -0.09031 | 0.34335 | -0.20011 |
| p__Firmicutes | c__Clostridia | o__Clostridiales | f__Ruminococcaceae | g__Ruminiclostridium | s__unclassified_g__Ruminiclostridium | OTU351 | -0.43218 | -0.60521 | -0.5222 | -0.67315 | -0.6489 | -0.63307 |
| p__Firmicutes | c__Bacilli | o__Lactobacillales | f__Leuconostocaceae | g__Leuconostoc | s__unclassified_g__Leuconostoc | OTU2 | 0.405881 | 0.205291 | 0.828742 | -0.02032 | 0.705734 | 0.053149 |
| p__Firmicutes | c__Bacilli | o__Lactobacillales | f__Lactobacillaceae | g__Lactobacillus | s__unclassified_g__Lactobacillus | OTU147 | 4.538035 | 3.758993 | -0.13369 | 3.449621 | 2.348871 | 3.028878 |
| p__Proteobacteria | c__Gammaproteobacteria | o__Enterobacteriales | f__Enterobacteriaceae | g__Escherichia-Shigella | s__unclassified_g__Escherichia-Shigella | OTU205 | 0.209228 | 1.443008 | 0.040787 | 0.610086 | 0.300815 | -0.45337 |
| p__Firmicutes | c__Erysipelotrichia | o__Erysipelotrichales | f__Erysipelotrichaceae | g__norank_f__Erysipelotrichaceae | s__uncultured_Allobaculum_sp._g__norank | OTU291 | -0.07735 | -0.60521 | -0.5222 | -0.02032 | -0.6489 | -0.31399 |
| p__Actinobacteria | c__Actinobacteria | o__Bifidobacteriales | f__Bifidobacteriaceae | g__Bifidobacterium | s__unclassified_g__Bifidobacterium | OTU266 | -0.61458 | -0.17282 | 0.040787 | -0.16966 | -0.6489 | -0.63307 |
| p__Proteobacteria | c__Gammaproteobacteria | o__Enterobacteriales | f__Enterobacteriaceae | g__unclassified_f__Enterobacteriaceae | s__unclassified_f__Enterobacteriaceae | OTU228 | 1.088207 | -0.42575 | 3.827792 | -0.16966 | 1.645589 | -0.63307 |
| p__Firmicutes | c__Bacilli | o__Bacillales | f__Family_XII_o__Bacillales | g__Exiguobacterium | s__unclassified_g__Exiguobacterium | OTU355 | 0.007312 | -0.60521 | 0.180101 | 0.198194 | 0.093372 | 1.433241 |
| p__Firmicutes | c__Bacilli | o__Lactobacillales | f__Lactobacillaceae | g__unclassified_f__Lactobacillaceae | s__unclassified_f__Lactobacillaceae | OTU10 | -0.61458 | -0.60521 | -0.72192 | -0.67315 | -0.6489 | -0.63307 |
| p__Firmicutes | c__Bacilli | o__Bacillales | f__Planococcaceae | g__Lysinibacillus | s__unclassified_g__Lysinibacillus | OTU88 | -0.61458 | -0.60521 | -0.5222 | -0.5022 | -0.6489 | -0.63307 |
| p__Deferribacteres | c__Deferribacteres | o__Deferribacterales | f__Deferribacteraceae | g__Mucispirillum | s__Mucispirillum_schaedleri_ASF457 | OTU256 | -0.61458 | -0.17282 | -0.13369 | 0.28323 | -0.6489 | -0.63307 |
| p__Proteobacteria | c__Gammaproteobacteria | o__Betaproteobacteriales | f__Burkholderiaceae | g__Janthinobacterium | s__unclassified_g__Janthinobacterium | OTU367 | -0.61458 | -0.60521 | -0.72192 | -0.67315 | -0.6489 | -0.45337 |
| p__Firmicutes | c__Clostridia | o__Clostridiales | f__Peptostreptococcaceae | g__Paeniclostridium | s__unclassified_g__Paeniclostridium | OTU222 | -0.61458 | -0.17282 | -0.72192 | -0.67315 | -0.6489 | -0.63307 |
| p__Bacteroidetes | c__Bacteroidia | o__Bacteroidales | f__Bacteroidaceae | g__Bacteroides | s__unclassified_g__Bacteroides | OTU192 | -0.17509 | 0.006637 | -0.36728 | -0.5022 | -0.6489 | -0.63307 |
| p__Proteobacteria | c__Gammaproteobacteria | o__Betaproteobacteriales | f__Burkholderiaceae | g__Paenalcaligenes | s__Paenalcaligenes_hominis | OTU383 | -0.43218 | 0.006637 | 0.040787 | -0.3696 | -0.22156 | 0.329126 |
| p__Firmicutes | c__Bacilli | o__Lactobacillales | f__Lactobacillaceae | g__Lactobacillus | s__unclassified_g__Lactobacillus | OTU319 | 0.703887 | 0.080111 | 1.022238 | 0.632513 | 2.4736 | 0.519873 |
| p__Firmicutes | c__Clostridia | o__Clostridiales | f__Lachnospiraceae | g__Lachnospiraceae_NK4A136_group | s__Lachnospiraceae_bacterium_10-1 | OTU295 | -0.61458 | -0.60521 | -0.72192 | 0.4237 | -0.6489 | -0.63307 |
| p__Proteobacteria | c__Gammaproteobacteria | o__Betaproteobacteriales | f__Burkholderiaceae | g__Paenalcaligenes | s__Paenalcaligenes_hominis | OTU146 | -0.07735 | -0.17282 | -0.5222 | -0.5022 | -0.12653 | -0.63307 |
| p__Deferribacteres | c__Deferribacteres | o__Deferribacterales | f__Deferribacteraceae | g__Mucispirillum | s__Mucispirillum_schaedleri_ASF457 | OTU327 | -0.17509 | -0.42575 | -0.72192 | 0.098925 | 0.489089 | -0.20011 |
| p__Firmicutes | c__Erysipelotrichia | o__Erysipelotrichales | f__Erysipelotrichaceae | g__Faecalibaculum | s__Faecalibaculum_rodentium | OTU151 | 0.754638 | 0.309501 | -0.5222 | 0.45418 | -0.33397 | -0.02042 |
| p__Firmicutes | c__Erysipelotrichia | o__Erysipelotrichales | f__Erysipelotrichaceae | g__Faecalibaculum | s__Faecalibaculum_rodentium | OTU156 | 0.778567 | -0.28655 | 0.661316 | -0.26126 | 0.152133 | -0.20011 |
| p__Actinobacteria | c__Actinobacteria | o__Micrococcales | f__Micrococcaceae | g__Glutamicibacter | s__unclassified_g__Glutamicibacter | OTU308 | -0.61458 | -0.60521 | -0.24071 | -0.26126 | -0.33397 | -0.63307 |
| p__Proteobacteria | c__Deltaproteobacteria | o__Desulfovibrionales | f__Desulfovibrionaceae | g__Desulfovibrio | s__unclassified_g__Desulfovibrio | OTU259 | -0.43218 | -0.28655 | -0.36728 | -0.26126 | -0.6489 | -0.45337 |
| p__Bacteroidetes | c__Bacteroidia | o__Bacteroidales | f__Rikenellaceae | g__Rikenellaceae_RC9_gut_group | s__unclassified_g__Rikenellaceae_RC9_gut_group | OTU93 | -0.61458 | -0.60521 | -0.72192 | -0.5022 | -0.47154 | -0.63307 |
| p__Proteobacteria | c__Gammaproteobacteria | o__Enterobacteriales | f__Enterobacteriaceae | g__unclassified_f__Enterobacteriaceae | s__unclassified_f__Enterobacteriaceae | OTU326 | 0.995253 | 0.309501 | 0.482319 | 0.242232 | 3.095265 | -0.02042 |
| p__Bacteroidetes | c__Bacteroidia | o__Bacteroidales | f__Muribaculaceae | g__norank_f__Muribaculaceae | s__unclassified_g__norank_f__Muribaculaceae | OTU56 | -0.61458 | -0.60521 | -0.72192 | -0.67315 | -0.6489 | -0.63307 |
| p__Bacteroidetes | c__Bacteroidia | o__Bacteroidales | f__Muribaculaceae | g__norank_f__Muribaculaceae | s__unclassified_g__norank_f__Muribaculaceae | OTU50 | -0.61458 | -0.60521 | -0.72192 | -0.67315 | -0.6489 | -0.63307 |
| p__Firmicutes | c__Erysipelotrichia | o__Erysipelotrichales | f__Erysipelotrichaceae | g__Faecalibaculum | s__Faecalibaculum_rodentium | OTU181 | -0.07735 | 0.944893 | -0.24071 | -0.26126 | -0.6489 | -0.63307 |
| p__Firmicutes | c__Clostridia | o__Clostridiales | f__Ruminococcaceae | g__Negativibacillus | s__unclassified_g__Negativibacillus | OTU28 | -0.61458 | -0.60521 | -0.5222 | -0.67315 | -0.47154 | -0.45337 |
| p__Bacteroidetes | c__Bacteroidia | o__Bacteroidales | f__Muribaculaceae | g__norank_f__Muribaculaceae | s__unclassified_g__norank_f__Muribaculaceae | OTU378 | -0.61458 | -0.60521 | -0.72192 | -0.67315 | -0.6489 | -0.63307 |
| p__Firmicutes | c__Bacilli | o__Lactobacillales | f__Lactobacillaceae | g__Lactobacillus | s__unclassified_g__Lactobacillus | OTU200 | 3.786478 | 4.556238 | 3.18002 | 3.397007 | 2.895727 | 3.338524 |
| p__Firmicutes | c__Bacilli | o__Lactobacillales | f__Lactobacillaceae | g__Lactobacillus | s__unclassified_g__Lactobacillus | OTU201 | 0.94311 | 2.370779 | -0.13369 | 0.632513 | 0.093372 | 0.582387 |
| p__Verrucomicrobia | c__Verrucomicrobiae | o__Verrucomicrobiales | f__Akkermansiaceae | g__Akkermansia | s__Akkermansia_muciniphila | OTU312 | -0.07735 | 0.145836 | 0.040787 | 0.042289 | 0.883097 | -0.31399 |
| p__Firmicutes | c__Clostridia | o__Clostridiales | f__Clostridiaceae_1 | g__Clostridium_sensu_stricto_4 | s__Clostridium_fallax | OTU67 | -0.61458 | -0.60521 | -0.36728 | -0.5022 | -0.6489 | -0.45337 |
| p__Bacteroidetes | c__Bacteroidia | o__Bacteroidales | f__Muribaculaceae | g__norank_f__Muribaculaceae | s__unclassified_g__norank_f__Muribaculaceae | OTU36 | -0.61458 | -0.60521 | -0.72192 | -0.67315 | -0.6489 | -0.63307 |
| p__Firmicutes | c__Bacilli | o__Lactobacillales | f__Lactobacillaceae | g__Lactobacillus | s__unclassified_g__Lactobacillus | OTU208 | 1.073631 | 2.820547 | 0.180101 | 1.021978 | 0.658286 | 1.058434 |
| p__Firmicutes | c__Bacilli | o__Lactobacillales | f__Leuconostocaceae | g__Weissella | s__unclassified_g__Weissella | OTU323 | -0.61458 | -0.60521 | -0.72192 | -0.67315 | -0.47154 | -0.63307 |
| p__Firmicutes | c__Bacilli | o__Lactobacillales | f__Lactobacillaceae | g__Lactobacillus | s__unclassified_g__Lactobacillus | OTU363 | 0.081992 | -0.60521 | -0.36728 | -0.02032 | 0.658286 | 4.141996 |
| p__Firmicutes | c__Bacilli | o__Lactobacillales | f__Lactobacillaceae | g__Lactobacillus | s__unclassified_g__Lactobacillus | OTU366 | -0.61458 | -0.60521 | -0.72192 | -0.67315 | -0.6489 | -0.45337 |
| p__Firmicutes | c__Clostridia | o__Clostridiales | f__Lachnospiraceae | g__unclassified_f__Lachnospiraceae | s__unclassified_f__Lachnospiraceae | OTU348 | -0.61458 | -0.60521 | -0.72192 | -0.67315 | -0.47154 | -0.63307 |
| p__Firmicutes | c__Bacilli | o__Lactobacillales | f__Leuconostocaceae | g__Weissella | s__unclassified_g__Weissella | OTU229 | 0.405881 | 0.006637 | 1.752946 | 0.45418 | 0.899989 | -0.20011 |
| p__Firmicutes | c__Clostridia | o__Clostridiales | f__Lachnospiraceae | g__unclassified_f__Lachnospiraceae | s__unclassified_f__Lachnospiraceae | OTU347 | 0.007312 | -0.17282 | -0.5222 | -0.5022 | -0.0442 | -0.20011 |
| p__Firmicutes | c__Bacilli | o__Lactobacillales | f__Lactobacillaceae | g__Lactobacillus | s__unclassified_g__Lactobacillus | OTU290 | 0.362136 | 0.080111 | 0.482319 | 0.949124 | 0.606879 | 0.282837 |
| p__Firmicutes | c__Erysipelotrichia | o__Erysipelotrichales | f__Erysipelotrichaceae | g__Faecalibaculum | s__Faecalibaculum_rodentium | OTU333 | 0.148796 | -0.60521 | -0.04098 | -0.3696 | 0.455756 | -0.31399 |
| p__Firmicutes | c__Bacilli | o__Lactobacillales | f__Lactobacillaceae | g__Lactobacillus | s__unclassified_g__Lactobacillus | OTU292 | 2.375291 | 1.624413 | 2.373178 | 2.505224 | 2.272869 | 2.2719 |
| p__Proteobacteria | c__Gammaproteobacteria | o__Betaproteobacteriales | f__Burkholderiaceae | g__Burkholderia-Caballeronia-Paraburkholderia | s__unclassified_g__Burkholderia-Caballeronia-Paraburkholderia | OTU160 | 1.043432 | 0.006637 | 0.240509 | -0.09031 | 0.74979 | -0.20011 |
| p__Firmicutes | c__Bacilli | o__Lactobacillales | f__Lactobacillaceae | g__Lactobacillus | s__unclassified_g__Lactobacillus | OTU193 | 3.406266 | 3.074621 | 2.68183 | 3.285803 | 2.669816 | 2.885042 |
| p__Proteobacteria | c__Gammaproteobacteria | o__Pseudomonadales | f__Pseudomonadaceae | g__Pseudomonas | s__unclassified_g__Pseudomonas | OTU83 | -0.61458 | -0.60521 | -0.13369 | -0.5022 | -0.6489 | -0.63307 |
| p__Firmicutes | c__Clostridia | o__Clostridiales | f__Ruminococcaceae | g__Oscillibacter | s__Oscillibacter_sp._1-3 | OTU22 | -0.43218 | -0.60521 | -0.24071 | -0.26126 | -0.33397 | -0.45337 |
| p__Firmicutes | c__Erysipelotrichia | o__Erysipelotrichales | f__Erysipelotrichaceae | g__norank_f__Erysipelotrichaceae | s__uncultured_Allobaculum_sp._g__norank | OTU257 | -0.61458 | -0.28655 | 0.040787 | 0.15063 | -0.6489 | -0.63307 |
| p__Firmicutes | c__Bacilli | o__Lactobacillales | f__Lactobacillaceae | g__Lactobacillus | s__unclassified_g__Lactobacillus | OTU105 | -0.61458 | -0.60521 | -0.72192 | -0.67315 | -0.6489 | -0.63307 |
| p__Firmicutes | c__Bacilli | o__Lactobacillales | f__Leuconostocaceae | g__Leuconostoc | s__unclassified_g__Leuconostoc | OTU219 | -0.61458 | 1.197825 | -0.24071 | -0.26126 | 0.255125 | -0.63307 |
| p__Firmicutes | c__Bacilli | o__Lactobacillales | f__Lactobacillaceae | g__Lactobacillus | s__unclassified_g__Lactobacillus | OTU103 | -0.61458 | 3.555801 | -0.72192 | 3.053283 | 0.300815 | 0.864706 |
| p__Proteobacteria | c__Gammaproteobacteria | o__Betaproteobacteriales | f__Burkholderiaceae | g__Burkholderia-Caballeronia-Paraburkholderia | s__unclassified_g__Burkholderia-Caballeronia-Paraburkholderia | OTU144 | -0.17509 | -0.42575 | 0.440231 | 0.28323 | 0.420516 | -0.45337 |
| p__Firmicutes | c__Bacilli | o__Lactobacillales | f__Lactobacillaceae | g__Lactobacillus | s__unclassified_g__Lactobacillus | OTU6 | 1.724351 | 2.117847 | 2.025651 | 1.107014 | 1.525177 | 1.538172 |
| p__Firmicutes | c__Bacilli | o__Lactobacillales | f__Lactobacillaceae | g__Lactobacillus | s__unclassified_g__Lactobacillus | OTU8 | -0.61458 | -0.60521 | -0.72192 | -0.67315 | -0.6489 | -0.63307 |
| p__Firmicutes | c__Bacilli | o__Lactobacillales | f__Lactobacillaceae | g__Lactobacillus | s__unclassified_g__Lactobacillus | OTU9 | -0.29069 | -0.42575 | 0.113933 | -0.3696 | -0.47154 | 0.118959 |
| p__Firmicutes | c__Bacilli | o__Lactobacillales | f__Lactobacillaceae | g__Lactobacillus | s__unclassified_g__Lactobacillus | OTU176 | 4.580442 | 1.905834 | 0.240509 | 1.439557 | 3.119865 | 1.622382 |
| p__Firmicutes | c__Bacilli | o__Lactobacillales | f__Lactobacillaceae | g__Lactobacillus | s__unclassified_g__Lactobacillus | OTU170 | 4.327898 | 2.107183 | -0.36728 | 2.481202 | 2.023789 | 2.279807 |
| p__Proteobacteria | c__Gammaproteobacteria | o__Enterobacteriales | f__Enterobacteriaceae | g__unclassified_f__Enterobacteriaceae | s__unclassified_f__Enterobacteriaceae | OTU316 | 0.866162 | 0.080111 | 0.87664 | -0.3696 | 2.946584 | 2.066489 |
| p__Firmicutes | c__Bacilli | o__Lactobacillales | f__Lactobacillaceae | g__Lactobacillus | s__unclassified_g__Lactobacillus | OTU173 | -0.43218 | -0.60521 | -0.72192 | -0.67315 | -0.6489 | -0.63307 |
| p__Firmicutes | c__Clostridia | o__Clostridiales | f__Ruminococcaceae | g__Ruminiclostridium | s__Clostridium]_leptum_g__Ruminiclostridium | OTU293 | -0.61458 | -0.60521 | -0.36728 | -0.26126 | -0.33397 | -0.20011 |
| p__Firmicutes | c__Clostridia | o__Clostridiales | f__Ruminococcaceae | g__Ruminiclostridium_9 | s__unclassified_g__Ruminiclostridium_9 | OTU234 | -0.17509 | -0.60521 | 0.180101 | 0.098925 | -0.47154 | -0.10383 |
| p__Firmicutes | c__Bacilli | o__Bacillales | f__Staphylococcaceae | g__Staphylococcus | s__unclassified_g__Staphylococcus | OTU32 | 0.264397 | -0.28655 | -0.5222 | -0.3696 | -0.22156 | -0.10383 |
| p__Proteobacteria | c__Gammaproteobacteria | o__Enterobacteriales | f__Enterobacteriaceae | g__unclassified_f__Enterobacteriaceae | s__unclassified_f__Enterobacteriaceae | OTU361 | 0.315148 | -0.07666 | 0.440231 | 0.15063 | 0.205777 | 1.538172 |
| p__Proteobacteria | c__Deltaproteobacteria | o__Desulfovibrionales | f__Desulfovibrionaceae | g__Bilophila | s__uncultured_bacterium_g__Bilophila | OTU375 | -0.43218 | -0.28655 | 0.113933 | -0.5022 | -0.0442 | 0.053149 |
| p__Firmicutes | c__Bacilli | o__Lactobacillales | f__Lactobacillaceae | g__Lactobacillus | s__unclassified_g__Lactobacillus | OTU35 | 1.400462 | 1.385888 | 1.058785 | 0.483173 | 1.256793 | 1.563412 |
| p__Firmicutes | c__Bacilli | o__Lactobacillales | f__Lactobacillaceae | g__Lactobacillus | s__unclassified_g__Lactobacillus | OTU33 | 0.866162 | 0.741892 | 0.522001 | 0.242232 | 0.633118 | 0.762079 |
| p__Firmicutes | c__Bacilli | o__Lactobacillales | f__Lactobacillaceae | g__Lactobacillus | s__unclassified_g__Lactobacillus | OTU30 | 1.102456 | 0.145836 | 0.113933 | -0.26126 | -0.12653 | 0.582387 |
| p__Firmicutes | c__Bacilli | o__Lactobacillales | f__Leuconostocaceae | g__Leuconostoc | s__unclassified_g__Leuconostoc | OTU136 | 0.007312 | 0.080111 | -0.04098 | 0.483173 | -0.33397 | -0.45337 |
| p__Bacteroidetes | c__Bacteroidia | o__Bacteroidales | f__Muribaculaceae | g__norank_f__Muribaculaceae | s__unclassified_g__norank_f__Muribaculaceae | OTU294 | -0.61458 | -0.60521 | -0.72192 | -0.16966 | -0.33397 | -0.63307 |
| p__Proteobacteria | c__Gammaproteobacteria | o__Pseudomonadales | f__Pseudomonadaceae | g__Pseudomonas | s__unclassified_g__Pseudomonas | OTU143 | -0.61458 | -0.60521 | -0.5222 | -0.67315 | -0.6489 | -0.63307 |
| p__Firmicutes | c__Clostridia | o__Clostridiales | f__Lachnospiraceae | g__unclassified_f__Lachnospiraceae | s__unclassified_f__Lachnospiraceae | OTU107 | -0.61458 | -0.28655 | -0.72192 | -0.16966 | -0.47154 | -0.31399 |
| p__Proteobacteria | c__Gammaproteobacteria | o__Enterobacteriales | f__Enterobacteriaceae | g__Escherichia-Shigella | s__unclassified_g__Escherichia-Shigella | OTU139 | 1.252339 | 0.25957 | 1.623745 | 0.835592 | 0.810481 | -0.31399 |
| p__Bacteroidetes | c__Bacteroidia | o__Bacteroidales | f__Bacteroidaceae | g__Bacteroides | s__unclassified_g__Bacteroides | OTU393 | -0.61458 | -0.60521 | -0.72192 | -0.5022 | -0.6489 | -0.20011 |
| p__Firmicutes | c__Clostridia | o__Clostridiales | f__Lachnospiraceae | g__unclassified_f__Lachnospiraceae | s__unclassified_f__Lachnospiraceae | OTU108 | -0.61458 | -0.60521 | -0.72192 | -0.67315 | -0.47154 | -0.63307 |
| p__Firmicutes | c__Bacilli | o__Lactobacillales | f__Streptococcaceae | g__Streptococcus | s__unclassified_g__Streptococcus | OTU17 | -0.43218 | -0.60521 | -0.24071 | -0.5022 | -0.33397 | -0.20011 |
| p__Proteobacteria | c__Gammaproteobacteria | o__Betaproteobacteriales | f__Burkholderiaceae | g__Burkholderia-Caballeronia-Paraburkholderia | s__unclassified_g__Burkholderia-Caballeronia-Paraburkholderia | OTU249 | -0.61458 | 0.006637 | 0.803494 | -0.3696 | -0.6489 | -0.31399 |
| p__Firmicutes | c__Bacilli | o__Lactobacillales | f__Lactobacillaceae | g__Lactobacillus | s__unclassified_g__Lactobacillus | OTU324 | 2.824174 | 2.641089 | -0.5222 | 3.529355 | 4.159821 | 2.465364 |
| p__Firmicutes | c__Bacilli | o__Lactobacillales | f__Lactobacillaceae | g__Lactobacillus | s__unclassified_g__Lactobacillus | OTU321 | 0.148796 | 0.309501 | -0.72192 | 1.785687 | 3.997326 | 0.118959 |
| p__Firmicutes | c__Bacilli | o__Lactobacillales | f__Lactobacillaceae | g__Lactobacillus | s__unclassified_g__Lactobacillus | OTU320 | 2.589982 | 0.994824 | -0.13369 | 2.289632 | 4.39755 | 2.814076 |
| p__Proteobacteria | c__Gammaproteobacteria | o__Cardiobacteriales | f__Wohlfahrtiimonadaceae | g__Ignatzschineria | s__swine_effluent_bacterium_CHNDP41 | OTU254 | -0.43218 | 0.006637 | 0.113933 | 0.098925 | -0.6489 | -0.63307 |
| p__Verrucomicrobia | c__Verrucomicrobiae | o__Verrucomicrobiales | f__Akkermansiaceae | g__Akkermansia | s__Akkermansia_muciniphila | OTU216 | -0.29069 | 0.788121 | 0.440231 | 0.242232 | -0.47154 | 0.053149 |
| p__Firmicutes | c__Bacilli | o__Lactobacillales | f__Lactobacillaceae | g__Lactobacillus | s__unclassified_g__Lactobacillus | OTU329 | -0.61458 | -0.60521 | -0.72192 | -0.67315 | 2.885165 | -0.63307 |
| p__Proteobacteria | c__Gammaproteobacteria | o__Alteromonadales | f__Shewanellaceae | g__Shewanella | s__unclassified_g__Shewanella | OTU232 | -0.61458 | -0.60521 | 0.395425 | -0.5022 | -0.47154 | -0.63307 |
| p__Firmicutes | c__Erysipelotrichia | o__Erysipelotrichales | f__Erysipelotrichaceae | g__Faecalibaculum | s__Faecalibaculum_rodentium | OTU373 | -0.61458 | -0.28655 | -0.04098 | -0.67315 | -0.12653 | 0.519873 |
| p__Proteobacteria | c__Gammaproteobacteria | o__Enterobacteriales | f__Enterobacteriaceae | g__Escherichia-Shigella | s__unclassified_g__Escherichia-Shigella | OTU11 | -0.43218 | -0.60521 | 0.482319 | 0.042289 | 0.152133 | 0.118959 |
| p__Firmicutes | c__Clostridia | o__Clostridiales | f__Ruminococcaceae | g__Ruminiclostridium_9 | s__unclassified_g__Ruminiclostridium_9 | OTU51 | -0.61458 | -0.60521 | -0.5222 | -0.67315 | -0.6489 | -0.63307 |
| p__Bacteroidetes | c__Bacteroidia | o__Bacteroidales | f__Muribaculaceae | g__norank_f__Muribaculaceae | s__unclassified_g__norank_f__Muribaculaceae | OTU148 | -0.07735 | -0.17282 | -0.72192 | -0.67315 | -0.47154 | -0.63307 |
| p__Firmicutes | c__Bacilli | o__Lactobacillales | f__Lactobacillaceae | g__Lactobacillus | s__unclassified_g__Lactobacillus | OTU374 | 0.081992 | 3.285085 | 1.484431 | 3.078215 | 2.394787 | 4.281276 |
| p__Firmicutes | c__Bacilli | o__Lactobacillales | f__Lactobacillaceae | g__Lactobacillus | s__unclassified_g__Lactobacillus | OTU252 | 0.081992 | 0.476846 | 4.389273 | 0.098925 | -0.6489 | -0.10383 |
| p__Proteobacteria | c__Gammaproteobacteria | o__Betaproteobacteriales | f__SC-I-84 | g__norank_f__SC-I-84 | s__unclassified_g__norank_f__SC-I-84 | OTU387 | -0.61458 | -0.60521 | -0.72192 | -0.67315 | -0.6489 | -0.45337 |
| p__Firmicutes | c__Clostridia | o__Clostridiales | f__Lachnospiraceae | g__Lachnoclostridium | s__Lachnospiraceae_bacterium_DW22 | OTU260 | -0.61458 | -0.60521 | -0.72192 | -0.5022 | -0.6489 | -0.45337 |
| p__Proteobacteria | c__Alphaproteobacteria | o__Sphingomonadales | f__Sphingomonadaceae | g__Sphingomonas | s__unclassified_g__Sphingomonas | OTU158 | -0.43218 | -0.60521 | -0.72192 | -0.67315 | -0.6489 | -0.63307 |
| p__Proteobacteria | c__Alphaproteobacteria | o__Azospirillales | f__Azospirillaceae | g__Azospirillum | s__unclassified_g__Azospirillum | OTU141 | -0.61458 | -0.60521 | -0.72192 | -0.67315 | -0.6489 | -0.63307 |
| p__Firmicutes | c__Bacilli | o__Lactobacillales | f__Lactobacillaceae | g__Lactobacillus | s__unclassified_g__Lactobacillus | OTU185 | -0.61458 | 1.509512 | -0.72192 | -0.67315 | -0.6489 | -0.63307 |
| p__Bacteroidetes | c__Bacteroidia | o__Bacteroidales | f__Muribaculaceae | g__norank_f__Muribaculaceae | s__unclassified_g__norank_f__Muribaculaceae | OTU114 | -0.61458 | -0.28655 | -0.13369 | -0.16966 | -0.12653 | -0.45337 |
| p__Actinobacteria | c__Actinobacteria | o__Coriobacteriales | f__Eggerthellaceae | g__Parvibacter | s__uncultured_bacterium_g__Parvibacter | OTU382 | -0.61458 | -0.60521 | -0.72192 | -0.67315 | -0.6489 | -0.31399 |
| p__Firmicutes | c__Clostridia | o__Clostridiales | f__Family_XI_o__Clostridiales | g__unclassified_f__Family_XI_o__Clostridiales | s__unclassified_f__Family_XI_o__Clostridiales | OTU318 | -0.43218 | -0.60521 | -0.72192 | -0.67315 | -0.33397 | -0.63307 |
| p__Proteobacteria | c__Gammaproteobacteria | o__Enterobacteriales | f__Enterobacteriaceae | g__unclassified_f__Enterobacteriaceae | s__unclassified_f__Enterobacteriaceae | OTU87 | 0.362136 | -0.60521 | 0.595147 | 0.654124 | 0.420516 | -0.63307 |
| p__Firmicutes | c__Clostridia | o__Clostridiales | f__Lachnospiraceae | g__Blautia | s__unclassified_g__Blautia | OTU182 | -0.29069 | 0.080111 | -0.24071 | -0.5022 | -0.47154 | -0.63307 |
| p__Firmicutes | c__Bacilli | o__Lactobacillales | f__Lactobacillaceae | g__Lactobacillus | s__unclassified_g__Lactobacillus | OTU131 | 3.020642 | 3.032152 | 2.477511 | 3.156171 | 2.904177 | 2.235027 |
| p__Firmicutes | c__Bacilli | o__Lactobacillales | f__Lactobacillaceae | g__Lactobacillus | s__unclassified_g__Lactobacillus | OTU134 | -0.43218 | -0.60521 | -0.72192 | -0.26126 | -0.6489 | -0.63307 |
| p__Verrucomicrobia | c__Verrucomicrobiae | o__Verrucomicrobiales | f__Akkermansiaceae | g__Akkermansia | s__Akkermansia_muciniphila | OTU57 | -0.17509 | -0.60521 | 0.559537 | -0.5022 | -0.33397 | -0.20011 |
| p__Bacteroidetes | c__Bacteroidia | o__Bacteroidales | f__Bacteroidaceae | g__Bacteroides | s__unclassified_g__Bacteroides | OTU162 | -0.07735 | -0.60521 | -0.5222 | -0.5022 | -0.22156 | -0.63307 |
| p__Firmicutes | c__Clostridia | o__Clostridiales | f__Lachnospiraceae | g__unclassified_f__Lachnospiraceae | s__unclassified_f__Lachnospiraceae | OTU322 | -0.61458 | 0.006637 | -0.72192 | -0.02032 | 0.152133 | -0.31399 |
| p__Firmicutes | c__Clostridia | o__Clostridiales | f__Ruminococcaceae | g__Ruminiclostridium | s__Clostridium]_leptum_g__Ruminiclostridium | OTU258 | -0.61458 | 0.006637 | -0.36728 | -0.3696 | -0.6489 | -0.20011 |
| p__Firmicutes | c__Clostridia | o__Clostridiales | f__Ruminococcaceae | g__norank_f__Ruminococcaceae | s__unclassified_g__norank_f__Ruminococcaceae | OTU127 | -0.61458 | -0.42575 | -0.72192 | -0.26126 | -0.6489 | -0.45337 |
| p__Proteobacteria | c__Gammaproteobacteria | o__Betaproteobacteriales | f__Burkholderiaceae | g__Paenalcaligenes | s__Paenalcaligenes_hominis | OTU73 | -0.61458 | -0.60521 | -0.72192 | -0.67315 | -0.6489 | -0.10383 |
| p__Firmicutes | c__Clostridia | o__Clostridiales | f__Clostridiaceae_1 | g__Hathewaya | s__Hathewaya_limosa | OTU1 | -0.43218 | -0.60521 | -0.72192 | -0.67315 | -0.6489 | -0.63307 |
| p__Firmicutes | c__Clostridia | o__Clostridiales | f__Ruminococcaceae | g__Oscillibacter | s__Clostridium]_leptum_g__Oscillibacter | OTU214 | -0.61458 | -0.17282 | -0.5222 | -0.67315 | -0.6489 | -0.45337 |
| p__Proteobacteria | c__Alphaproteobacteria | o__Sphingomonadales | f__Sphingomonadaceae | g__Sphingomonas | s__unclassified_g__Sphingomonas | OTU94 | -0.61458 | -0.42575 | -0.72192 | -0.16966 | -0.33397 | -0.63307 |
| p__Firmicutes | c__Bacilli | o__Lactobacillales | f__Streptococcaceae | g__Lactococcus | s__unclassified_g__Lactococcus | OTU227 | 0.315148 | -0.07666 | 2.685259 | 0.632513 | 1.927854 | -0.31399 |
| p__Proteobacteria | c__Gammaproteobacteria | o__Enterobacteriales | f__Enterobacteriaceae | g__Proteus | s__unclassified_g__Proteus | OTU60 | -0.61458 | -0.60521 | -0.72192 | -0.67315 | -0.6489 | -0.63307 |
| p__Firmicutes | c__Bacilli | o__Bacillales | f__Bacillaceae | g__Bacillus | s__unclassified_g__Bacillus | OTU242 | 0.007312 | 0.006637 | 0.750064 | 0.357606 | -0.0442 | 0.450399 |
| p__Firmicutes | c__Clostridia | o__Clostridiales | f__Ruminococcaceae | g__Ruminococcaceae_UCG-014 | s__unclassified_g__Ruminococcaceae_UCG-014 | OTU213 | -0.61458 | -0.42575 | -0.72192 | -0.67315 | -0.6489 | -0.63307 |
| p__Firmicutes | c__Clostridia | o__Clostridiales | f__Lachnospiraceae | g__Lachnospiraceae_NK4A136_group | s__Lachnospiraceae_bacterium_10-1 | OTU267 | -0.29069 | 0.512502 | 1.258507 | 0.895065 | -0.33397 | -0.20011 |
| p__Firmicutes | c__Clostridia | o__Clostridiales | f__Ruminococcaceae | g__unclassified_f__Ruminococcaceae | s__unclassified_f__Ruminococcaceae | OTU273 | -0.61458 | -0.42575 | -0.36728 | -0.5022 | -0.6489 | -0.63307 |
| p__Firmicutes | c__Erysipelotrichia | o__Erysipelotrichales | f__Erysipelotrichaceae | g__Faecalibaculum | s__Faecalibaculum_rodentium | OTU64 | -0.29069 | -0.28655 | 0.113933 | -0.5022 | 0.34335 | 0.329126 |
| p__Proteobacteria | c__Alphaproteobacteria | o__Rhizobiales | f__Beijerinckiaceae | g__Methylobacterium | s__unclassified_g__Methylobacterium | OTU278 | -0.61458 | -0.42575 | -0.72192 | -0.26126 | -0.6489 | -0.63307 |
| p__Firmicutes | c__Bacilli | o__Lactobacillales | f__Lactobacillaceae | g__Pediococcus | s__unclassified_g__Pediococcus | OTU100 | 0.823876 | 2.008328 | 1.217253 | 2.290984 | 1.857984 | 1.749258 |
| p__Proteobacteria | c__Alphaproteobacteria | o__Sphingomonadales | f__Sphingomonadaceae | g__Sphingomonas | s__unclassified_g__Sphingomonas | OTU381 | -0.61458 | 0.080111 | 0.040787 | -0.5022 | -0.6489 | 0.739363 |
| p__Bacteroidetes | c__Bacteroidia | o__Bacteroidales | f__Muribaculaceae | g__norank_f__Muribaculaceae | s__unclassified_g__norank_f__Muribaculaceae | OTU46 | -0.61458 | -0.60521 | -0.5222 | -0.67315 | -0.6489 | -0.63307 |
| p__Proteobacteria | c__Gammaproteobacteria | o__Enterobacteriales | f__Enterobacteriaceae | g__unclassified_f__Enterobacteriaceae | s__unclassified_f__Enterobacteriaceae | OTU178 | 3.258045 | -0.60521 | 0.347527 | -0.67315 | 0.420516 | 0.329126 |
| p__Proteobacteria | c__Gammaproteobacteria | o__Betaproteobacteriales | f__Burkholderiaceae | g__Paenalcaligenes | s__Paenalcaligenes_hominis | OTU225 | -0.07735 | -0.60521 | 0.721723 | -0.3696 | -0.22156 | -0.63307 |
| p__Bacteroidetes | c__Bacteroidia | o__Bacteroidales | f__Muribaculaceae | g__norank_f__Muribaculaceae | s__uncultured_Bacteroidales_bacterium_g__norank_f__Muribaculaceae | OTU313 | -0.61458 | -0.60521 | -0.72192 | -0.5022 | -0.6489 | -0.45337 |
| p__Chloroflexi | c__KD4-96 | o__norank_c__KD4-96 | f__norank_c__KD4-96 | g__norank_c__KD4-96 | s__unclassified_g__norank_c__KD4-96 | OTU43 | -0.61458 | -0.60521 | -0.72192 | -0.67315 | -0.6489 | -0.63307 |
| p__Firmicutes | c__Clostridia | o__Clostridiales | f__Lachnospiraceae | g__Ruminococcus]_torques_group | s__unclassified_g__Ruminococcus]_torques_group | OTU150 | -0.43218 | -0.60521 | -0.72192 | -0.5022 | -0.6489 | -0.63307 |
| p__Firmicutes | c__Bacilli | o__Lactobacillales | f__Lactobacillaceae | g__Lactobacillus | s__unclassified_g__Lactobacillus | OTU217 | 0.405881 | 4.696338 | -0.72192 | 2.588562 | 2.179926 | 2.764566 |
| p__Firmicutes | c__Bacilli | o__Lactobacillales | f__Leuconostocaceae | g__Weissella | s__unclassified_g__Weissella | OTU356 | 0.007312 | -0.42575 | 0.180101 | 0.242232 | 0.152133 | 2.447601 |
| p__Proteobacteria | c__Gammaproteobacteria | o__Enterobacteriales | f__Enterobacteriaceae | g__unclassified_f__Enterobacteriaceae | s__unclassified_f__Enterobacteriaceae | OTU279 | 0.007312 | 0.080111 | 2.259877 | 3.101797 | 1.375395 | 0.37222 |
| p__Firmicutes | c__Bacilli | o__Lactobacillales | f__Lactobacillaceae | g__Lactobacillus | s__unclassified_g__Lactobacillus | OTU76 | 2.663762 | 1.883148 | 2.673185 | 3.442662 | 2.206513 | 1.440812 |
